# Insights into Nd^III^ to Yb^III^ Energy Transfer and Its Implications in Luminescence Thermometry

**DOI:** 10.1021/acs.chemmater.4c00362

**Published:** 2024-03-28

**Authors:** Mariangela Oggianu, Valentina Mameli, Miguel A. Hernández-Rodríguez, Noemi Monni, Manuel Souto, Carlos D.S. Brites, Carla Cannas, Fabio Manna, Francesco Quochi, Enzo Cadoni, Norberto Masciocchi, Albano N. Carneiro Neto, Luís D. Carlos, Maria Laura Mercuri

**Affiliations:** † Dipartimento di Scienze Chimiche e Geologiche, Università degli Studi di Cagliari, Monserrato I-09042, Italy; ‡ INSTM, Via Giuseppe Giusti, 9, Firenze 50121, Italy; § Phantom-g, Department of Physics, CICECO-Aveiro Institute of Materials, University of Aveiro, Aveiro 3810-193, Portugal; ∥ Department of Chemistry, CICECO-Aveiro Institute of Materials, University of Aveiro, Aveiro 3810-193, Portugal; ⊥ Dipartimento di Fisica, Università degli Studi di Cagliari, Complesso Universitario di Monserrato, Monserrato I-09042, Italy; # Dipartimento di Scienza e Alta Tecnologia & To.Sca.Lab., Università degli Studi dell, via Valleggio 11, Como 22100, Italy

## Abstract

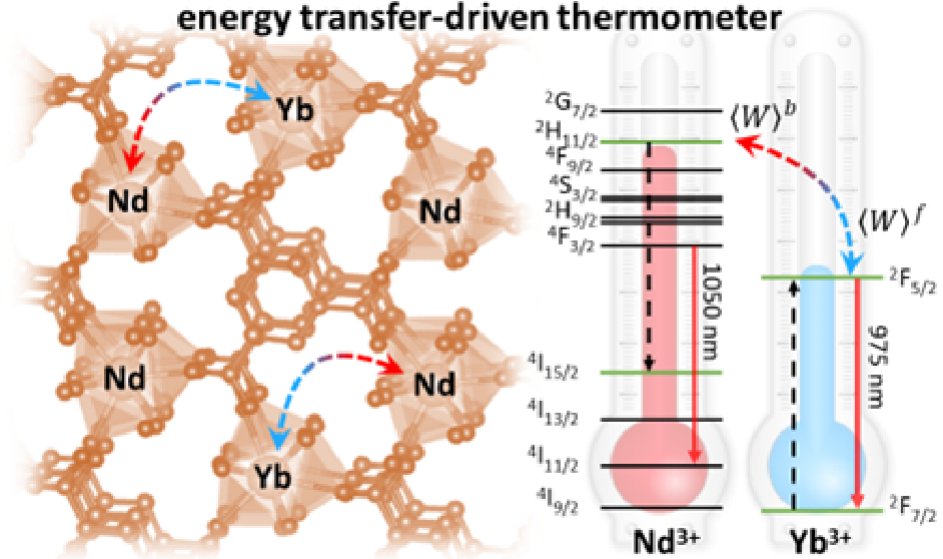

This work challenges the conventional approach of using Nd^III 4^F_3/2_ lifetime changes for evaluating the experimental Nd^III^ → Yb^III^ energy transfer rate and efficiency. Using near-infrared (NIR) emitting Nd:Yb mixed-metal coordination polymers (CPs), synthesized via solvent-free thermal grinding, we demonstrate that the Nd^III^ [^2^H_11/2_ → ^4^I_15/2_] → Yb^III^ [^2^F_7/2_ → ^2^F_5/2_] pathway, previously overlooked, dominates energy transfer due to superior energy resonance and *J*-level selection rule compatibility. This finding upends the conventional focus on the Nd^III^ [^4^F_3/2_ → ^4^I_11/2_] → Yb^III^ [^2^F_7/2_ → ^2^F_5/2_] transition pathway. We characterized Nd_0.890_Yb_0.110_(BTC)(H_2_O)_6_ as a promising cryogenic NIR thermometry system and employed our novel energy transfer understanding to perform simulations, yielding theoretical thermometric parameters and sensitivities for diverse Nd:Yb ratios. Strikingly, experimental thermometric data closely matched the theoretical predictions, validating our revised model. This novel perspective on Nd^III^ → Yb^III^ energy transfer holds general applicability for the Nd^III^/Yb^III^ pair, unveiling an important spectroscopic feature with broad implications for energy transfer-driven materials design.

## Introduction

Temperature is a critical physical parameter and its accurate detection is of paramount importance in many research fields ranging from climate, metrology, aerospace, nanomedicine, production plants, and food storage.^1,2^ During the past decade, novel temperature sensors have emerged that have the potential to substitute resistance thermometry.^3^ All examples are based on temperature-induced changes in the material’s chemical and physical properties, such as volume, electrical conductivity, or photoluminescence.

Among these is luminescence thermometry, which was developed as a remote temperature sensing technique that relies on the temperature dependency of luminescence features (e.g., band shape, peak energy or intensity, and excited state lifetimes and risetimes) of a phosphor to measure temperature.^4−8^ This technique provides precise thermal readouts with superior spatial resolution in short acquisition times. Noticeably, luminescent thermometers can operate in distinct temperature regions, from cryogenic temperatures (<100 K), of interest in cryobiology, aerospace, nuclear fusion, and the development of superconducting magnets,^9−13^ to high temperatures (>400 K) with potential applications in heavy industry,^14^ covering also the so-called physiological temperature range (298–323 K), of interest in biomedicine.^15^

Up to now, a plethora of luminescent materials, such as quantum dots, organic dyes, lanthanide-doped nanoparticles, and lanthanide complexes have been largely investigated for luminescence thermometry.^5^ Among them, trivalent lanthanide (Ln^III^) ions, including chelate complexes,^16,17^ polymers,^18,19^ metal–organic frameworks (MOFs),^20,21^ and organic–inorganic hybrids molecular probes,^22,23^ are promising materials for thermal sensing, given their typical long lifetimes (10^–3^ s range), characteristic sharp emission, and high emission quantum yields, emitting in the ultraviolet, visible, and near-infrared spectral regions.^17,24,25^

It is well-known that Ln^III^ centers cannot efficiently absorb light due to forbidden 4f–4f transitions.^26^ To overcome this, a challenging strategy for the fabrication of highly performant Ln-based materials, including luminescent thermometers, lies in the incorporation of luminescent linkers as a suitable *antenna*, thereby being able to absorb and transfer energy to the Ln^III^ centers.^27^ Lanthanide-based coordination polymers (CPs) and MOFs are excellent candidates for optical sensors due to their ability to show both ligand-centered and metal-centered luminescence. The proper choice of the luminescent building blocks, both Ln^III^ ions (metallic nodes) and functional organic linkers, is crucial to designing new CPs for thermal sensing applications showing different pathways of energy exchange, including intensity-based and ratiometric thermometers.^28^

Recently, mixed Ln’Ln”-MOFs thermometers have been developed where the intensity ratio of two emissions from different lanthanide ions, commonly Tb^III^ and Eu^III^, is used as the thermometric parameter,^20,21,29,30^ based on emissions in the visible spectral range. Cui et al. reported on the first example of a luminescent thermometer, based on mixed-Eu^III^/Tb^III^ MOF and Eu_0.0069_Tb_0.9931_-DMBDC (DMBDC = 2,5-dimethoxy-1,4-benzene-dicarboxylate), showing a significant temperature-dependent photoluminescence in the 10–300 K range.^20^ Besides the Eu^III^/Tb^III^ pair, the Nd^III^ and Yb^III^ ions have been receiving growing interest given their harmless emission wavelength and deep penetration length in biological tissues.^31−38^ However, all of the works reported so far rely on an unpredictable serendipitous approach, lacking a comprehensive elucidation of the thermometric performance rooted in donor-to-acceptor energy transfer mechanisms between Ln^III^ ions. This absence of understanding hinders the rational optimization of these materials.

Therefore, in this study, we employ a novel class of NIR-emitting Yb/Nd CPs based on the 1,3,5-benzentricarboxylic acid (H_3_BTC) organic linker to deeply investigate the Nd–Yb energy transfer process and quantitatively illustrate its influence on the thermometric properties of the materials. Then, the objective of the manuscript is to fully understand the underlying energy transfer mechanisms, and their crucial implications for optimizing energy transfer-driven ratiometric luminescent thermometers, rather than looking for a higher thermometric performance. Two different classes have been synthesized through a solvent-free thermal grinding method, formulated as Nd_*x*_Yb_(1–*x*)_(BTC)(H_2_O)_6_ (*x* = 1 (**1**); *x* = 0.943 (**2**); *x* = 0.953 (**3**); *x* = 0.890 (**4**)) and Nd_*x*_Yb_(1–*x*)_(BTC) (*x* = 0.017 (**5**), *x* = 0 (**6**)). Single lanthanide Nd-CPs and Yb-CPs have also been prepared as reference samples. The obtained materials have been morphologically, structurally, and thermally characterized and their photophysical processes (10–300 K) have been studied to determine the temperature dependence of the Nd^III^-to-Yb^III^ energy transfer processes in a representative sample, Nd_0.890_Yb_0.110_(BTC)(H_2_O)_6_**(4**) using the Nd-BTC (**1**) as a reference.

We demonstrate, both from experimental measurements and theoretical calculations, that the experimental Ln-to-Ln energy transfer rate ()^39^
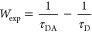
1and the energy transfer efficiency ()^40,41^
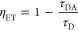
2

( and  are the lifetimes of the emitting level of the donor in the absence and presence of acceptors, respectively) are not valid for the specific case of Nd^III^–Yb^III^ energy transfer when Nd^III 4^F_3/2_ → ^4^I_11/2_ emission lifetimes are monitored for Nd(BTC)(H_2_O)_6_ () and Nd_*x*_Yb_(1–*x*)_(BTC)(H_2_O)_6_ () CPs. We can anticipate that the reason behind this is that the ^4^F_3/2_ → ^4^I_11/2_ is not an effective pathway in the Nd^III^–Yb^III^ energy transfer process as other Nd^III^ transitions (e.g., ^2^H_11/2_ → ^4^I_15/2_) are responsible for the energy transfer process. Thus, a new point of view regarding the Nd^III^–Yb^III^ energy transfer for excitation energies not being resonant with the ^4^F_3/2_ energy level is herein pointed out, contrary to what is reported in the literature.^40,42−47^

### Experimental Section

#### Synthesis

**Scheme 1:**
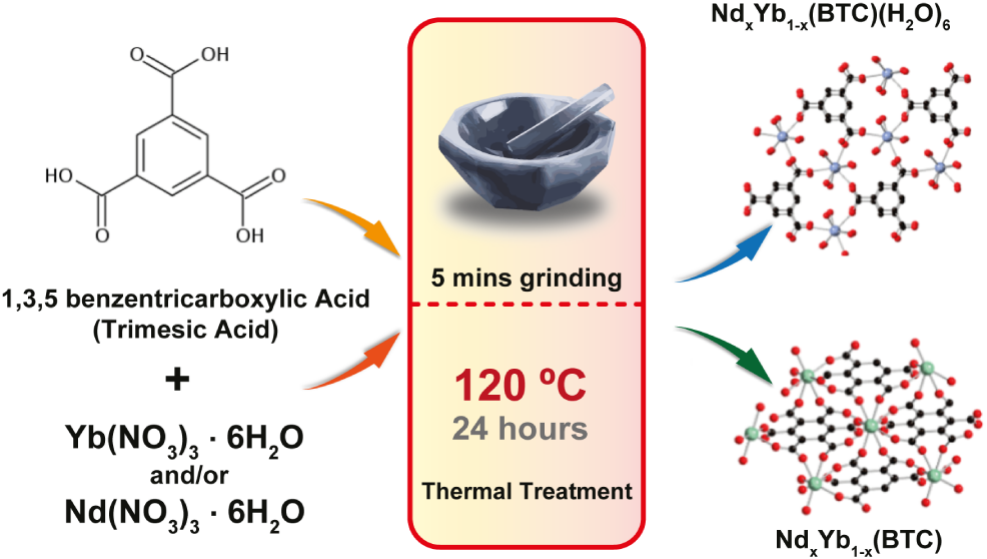
Schematic Representation of the Thermal Grinding Process

NIR emitter-based Ln′Ln″-CPs-CPs were synthesized through a solvent-free grinding method. Ln(NO_3_)_3_·6(H_2_O) (Ln^III^ = Yb, Nd) and 1,3,5-benzenetricarboxylic acid (H_3_BTC, trimesic acid) were mixed in a 1:1 ratio and ground for 5 min, and then thermally treated at 120 °C for 24 h, exploiting both mechanical and thermal energies (Scheme 1). This method, as reported by Liu et al.,^48^ offers a valid alternative for rapid, eco-friendly, and large-scale preparation of luminescent Ln-CPs/MOFs, avoiding the production of a large amount of solvent waste.

By adding to the precursor mixture a second Ln^III^ ion, with molar ratio in the 5 to 20%, compounds formulated as Nd_*x*_Yb_(1–*x*)_(BTC)(H_2_O)_6_ (*x* = 1 (**1**); *x* = 0.943 (**2**); *x* = 0.953 (**3**); *x* = 0.890 (**4**)) and Nd_*x*_Yb_(1–*x*)_BTC (*x* = 0.017 (**5**), *x* = 0 (**6**)) are obtained in the form of microcrystalline powder.

The materials have been fully characterized by powder X-ray diffraction (PXRD) and Fourier transform infrared spectroscopy (FT-IR), induced coupled plasma mass spectrometry (ICP-MS), scanning electron microscopy–energy dispersive X-ray (SEM-EDX) and thermal gravimetric analysis (TGA).

The synthetic process was monitored by PXRD, as shown in Figure S1 for the formation of Yb(BTC). When the precursors of **6** [H_3_BTC acid and Yb(NO_3_)_3_·6(H_2_O)] are mixed and milled for 30 s in an agate mortar, no formation of new compounds was detected. However, just after 1 min of milling, most of the precursor’s diffraction peaks disappeared, and a few barely visible peaks, attributed to residual trimesic acid, remained. For longer milling times (3 to 5 min), a sort of amorphization process occurred, confirming the hypothesis originally provided by Liu et al.;^48^ diffraction peaks progressively disappear, and the background level increases and becomes nonmonotonic. At this stage, the milled powders turned into a slurry. Treating this slurry at 120 °C led, after water elimination, to the formation of the desired Yb(BTC) CP. To verify if the grinding step had a direct role in the formation of Yb(BTC), the pristine mixture was also thermally treated, skipping the milling step. The PXRD analysis (see Figure S1) showed the obtainment of unknown crystal phases together with some residual reactants. Thus, the residual water present in the slurry could favor the reaction, as liquid-assisted grinding is used to facilitate mechanochemical reactions in disparate fields.^49−51^ Finally, the addition of a washing step by water and then ethanol allowed for purification of the product in the case of residues of the precursor mixture.

#### Crystal Structures

**Figure 1:**
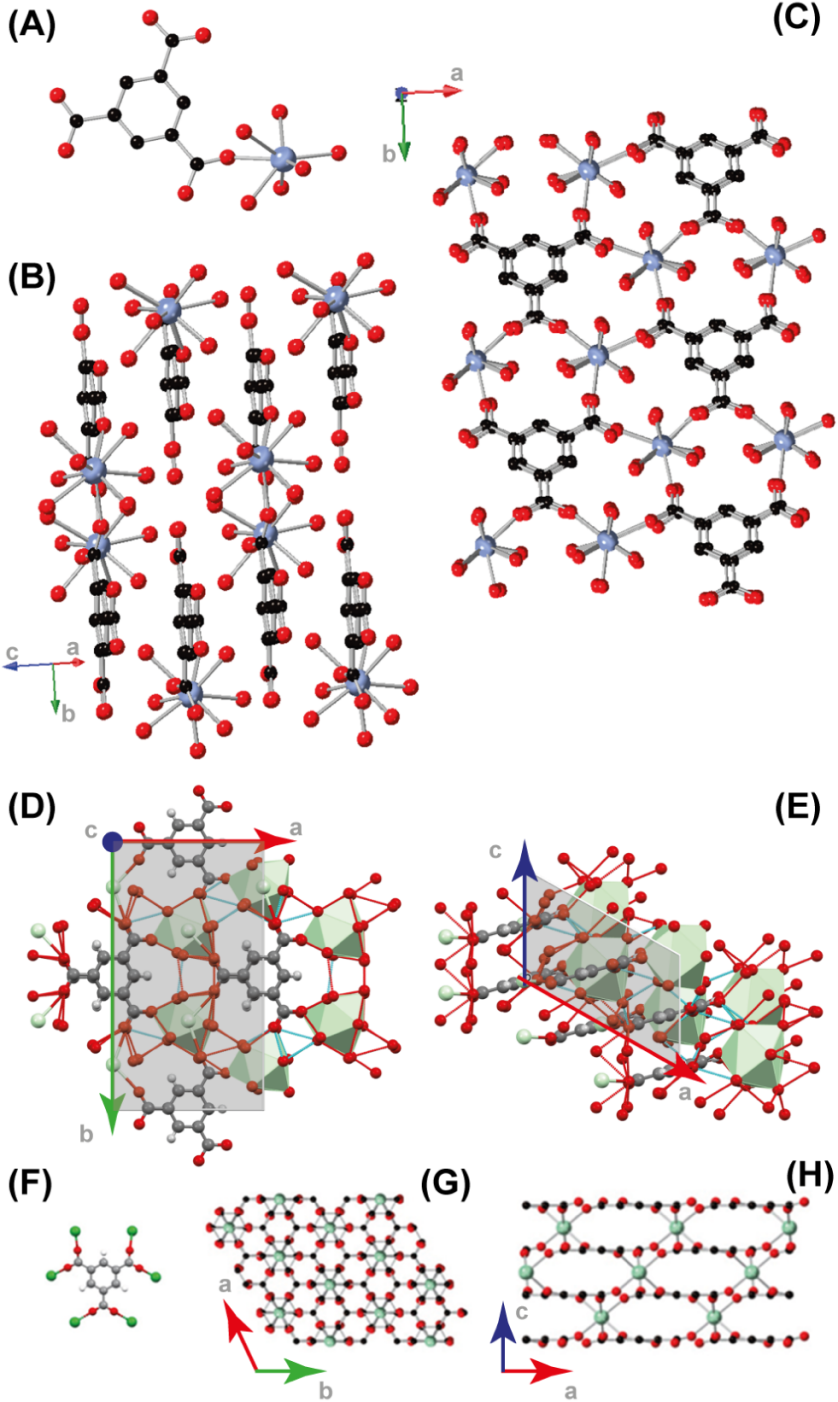
View of (A) the asymmetric unit of **1**, (B) 1D ribbons running parallel to the *b*-axis, and (C) stacked ribbons in the *ab* plane [001 view]. Sketch of the rich 3D hydrogen-bond framework (dashed red contacts) viewed along the (D) *c*- and (E) *b*-axis. View of (F) one BTC linker coordinating six Yb^III^ ions, (G) 3D CP in the *ab* plane, showing Yb^III^ ions stacked along the same *c* axis of aromatic BTC ring, (H) 3D CP in the *ac* plane, evidencing Yb^III^ and BTC lying in different planes. The black, red, and pastel (green) spheres represent the C, O, and Nd (Yb) atoms, respectively, while H atoms are omitted for clarity.

Compounds **1**–**6** are obtained as microcrystalline powders only. Hence, to determine their crystal structure, we resorted to PXRD (performed on **1** and **6** samples only), due to the isomorphous character of species **1** to **4** and, separately, **5** and **6**. Compound **1** crystallizes in the monoclinic space group *Cc*, as a neutral polymeric framework, isostructural with a previously reported Gd^III^-based species.^52^ The asymmetric unit of **1**, shown in Figure 1A, consists of one Nd^III^ ion, one fully deprotonated BTC unit, and six water molecules, all bound to the rare earth cation. Each metal ion is *ennea*-coordinated, surrounded by nine oxygen atoms, three from (three different) BTC linkers and six from water molecules, leading to the Nd(BTC)(H_2_O)_6_ formulation. The Nd^III^ ions, linked through BTC ligands, form parallel 1D ribbons running along the *b* ´́axis, as reported in Figure 1B. The stacking of these ribbons interconnected through a bevy of hydrogen bonds involving the H atoms of the coordinating water molecules, leads to a dense 3D framework (Figure 1C).

Figure 1D,E depicts the *ennea*-coordinated Nd atoms as green polyhedra and dashed red contacts indicate the rich 3D framework of hydrogen bonding, interlinking the different coordination spheres: the Nd–O bond distances are reported in Table S1. Note that, as restraints were introduced in the final Rietveld refinement cycles to stabilize the otherwise untreatable refinement diverging to an unphysical model, the obtained values mostly mirrored the numerical limits imposed of the Nd–O distances rather than their true similarity, or their dispersion.

Thermogravimetric analysis showed that all six coordinated water molecules can be completely removed by heating **1**, the hexahydrate Nd(BTC)(H_2_O)_6_ phase, at 130 °C (*vide infra*), leading to an amorphous material. The anhydrous Yb(BTC) (**6**) crystallizes as a complex 3D framework in the trigonal *R3̅c* space group. Its asymmetric unit consists of (a fraction of) one Yb^III^ ion and 1/6 of the fully deprotonated BTC ligand (both lying on special positions of *-3* point symmetry). The coordination sphere of each metal ion is composed of six oxygen atoms belonging to six distinct BTC linkers with a Yb–O bond distance of 2.259(3) Å, which, in turn, coordinate six Yb^III^ ions in the μ_6_ bridging coordination mode (Figure 1F).

This generates a 3D CP, where BTC aromatic rings, orderly stacked along the *c*-axis (Figure 1G), show intercalated Yb^III^ ions located midway at a distance of 1.56 Å (*c*/12) from the virtual plane containing the neighboring (and coordinating) BTC moieties. Indeed, when **6** is observed in the *ac* plane, as shown in Figure 1H, it is evident how all the Yb^III^ ions lie on a different, but parallel, plane than BTC linkers, forming a dense structure with no accessible cavities, with nonbonding Yb^III^⋯Yb^III^ distances of 6.00 (out of plane) and 8.88 Å (in plane), heavily minimizing Coulombic repulsion.

**Figure 2:**
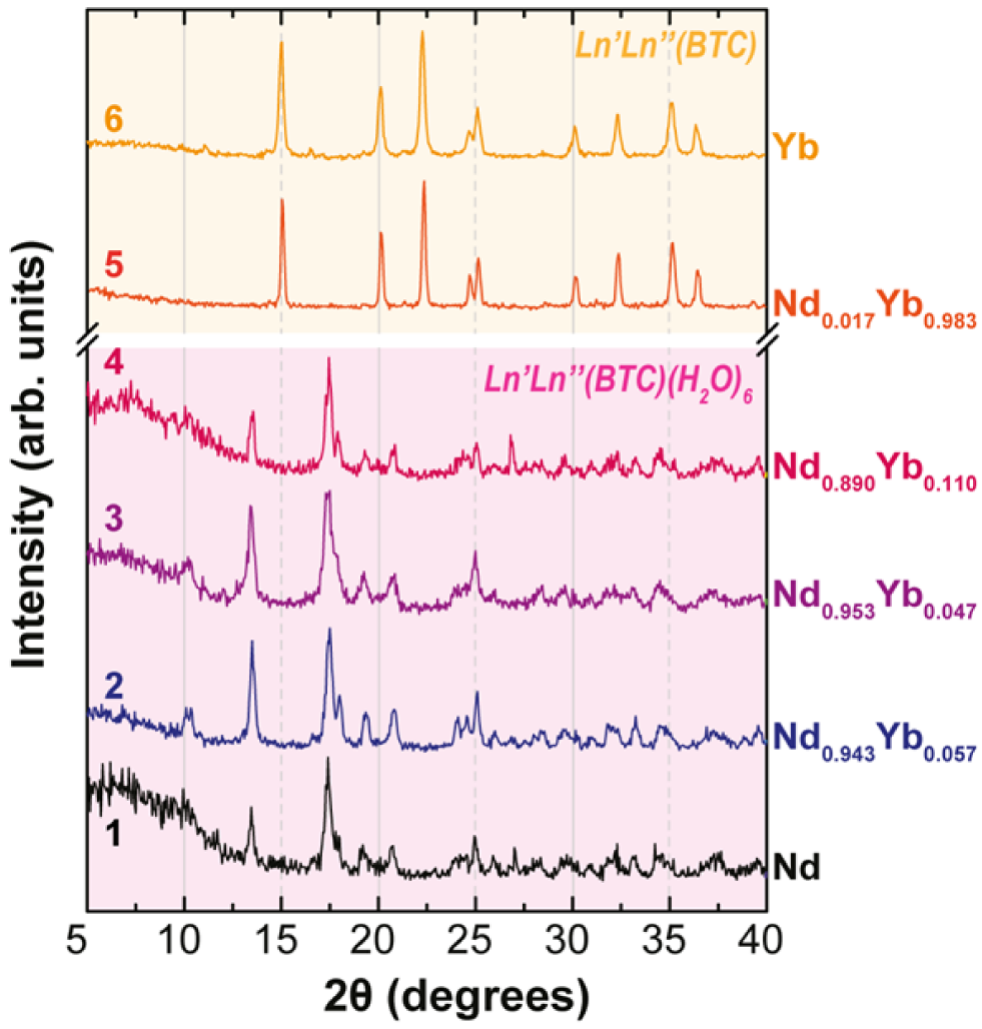
Powder X-ray diffraction patterns in the 5–40° 2θ range.

In line with the structural model presented here, thermogravimetric analysis confirmed the anhydrous character of **6** (*vide infra*). The PXRD analysis (Figure 2) confirmed the obtainment of two different structures (*vide supra*), depending on the x content: the Nd-rich samples (**2**–**4**) feature the structure of the hydrated Nd(BTC)(H_2_O)_6_–CP (**1**), whereas the Yb-rich samples **5** and **6** are anhydrous and isostructural. Other intermediate substitution ratios (Nd:Yb 50:50 and 30:70) were also tested (Figure S2), but PXRD revealed the formation of polyphasic mixtures where hydrated and anhydrous phases coexist. Thus, it appears that Nd^III^ and Yb^III^ act as structure-directing agents for the hydrated and anhydrous phases, respectively, if they dominate in the precursor’s mixture.

The results from our complete PXRD analysis also include an estimate of the specific surface areas (SSA) of our materials. As derived by the numerical analysis described in Text S1, SSA values fall well below 100 m^2^·g^–1^ (in line with the experimental BET values reported in the Experimental section). Note that porous materials possess much larger SSA values (in m^2^·g^–1^, 300–5000 for activated carbons, 1000 for zeolites, and up to 10000 for MOFs). Thus, as anticipated, our materials are nonporous and, accordingly, cannot be classified as MOFs.^53,54^

FT-IR spectra of **1**–**6** CPs show the typical bands assigned to the symmetric and antisymmetric stretching vibrations of carboxylic groups of trimesic acid^55^ (near 1700 cm^–1^ and in the 1650–1550 and 1450–1350 cm^–1^ ranges, Figure S4). Since carboxylates can coordinate in different modes (monodentate, bidentate, bridging, etc.), the frequency separation between the carboxylate antisymmetric and symmetric stretching vibrations (Δ*v*_a-s_) can be related to the different coordination modes.^56^ In fact, in **1**–**6**, Δ*v*_a-s_ (falling in the 170–180 cm^–1^ range) can be assigned, in agreement with the aforementioned crystal structures, to the bridging coordination mode. All **1**–**6** CPs show Ln–O stretching vibrational bands in the 600–400 cm^–1^ region.^57^

#### Thermogravimetric Analysis

TGA thermograms of **1** and **6** were measured to study the thermal stability and confirm the water content of the two representative structures. **1** shows four weight losses: a first one (below 70 °C) of about 1.5%, related to residual moisture; a second one of about 20% at 106 °C, and a third one of 4% at 298 °C, consistent with the elimination of the six water molecules. At higher temperatures, 500–700 °C, probably the three carboxylate groups are lost, as previously reported for metal-BTC MOFs,^58,59^ and a 40% weight loss is attributed to the decomposition to Nd_2_O_3_ (Figure S5a). The thermogram of **6** confirms the absence of water in the structure and its thermal stability up to 400 °C. A 6% sharp loss at about 420 °C is due to a small amount of trimesic acid impurity (see Figure 2, PXRD peak at about 11°). A further increase in the temperature led to a >40% weight loss, with the formation of Yb_2_O_3_ above 500 °C (Figure S5b). Variable temperature X-ray diffraction analyses, described in detail in Text S2, enabled structural variations and quantitative measurement of anisotropic thermal expansion effects, demonstrating, *inter alia*, the stability range of these materials when heated in air.

#### Photophysical Properties

To further probe the presence of both metals in the same CPs, diffuse reflectance (DR) spectra were performed in the 200–2000 nm range. DR spectra of **1**–**6** show absorption bands at ∼1970, 1660, and 290 nm, due to the BTC linker. In **1**–**4** compounds (reported in Figure S7a), the absorption bands of Nd^III^ ions are observed at 872 nm (^4^I_9/2_ → ^4^F_3/2_), 798 nm (^4^I_9/2_ → ^4^F_5/2_), 740 nm (^4^I_9/2_ → ^4^F_7/2_), 680 nm (^4^I_9/2_ → ^4^F_9/2_), 578 nm (^4^I_9/2_ → ^2^G_7/2_), 524 nm (^4^I_9/2_ → ^4^G_7/2_ + ^4^G_9/2_), 513 nm (^4^I_9/2_ → ^4^G_9/2_), and 355 nm (^4^I_9/2_ → ^4^D_5/2_ + ^4^D_3/2_). In **2**–**4**, a further band at 980 nm is observed, confirming the presence of Yb^III^ (^2^F_7/2_ → ^2^F_5/2_) in the CPs.^60−62^ Compound **5** shows the absorption bands of both Nd^III^ and Yb^III^ ions when compared to **6**, which exhibits only the Yb^III^ absorption band, as shown in Figure S7.

**Figure 3:**
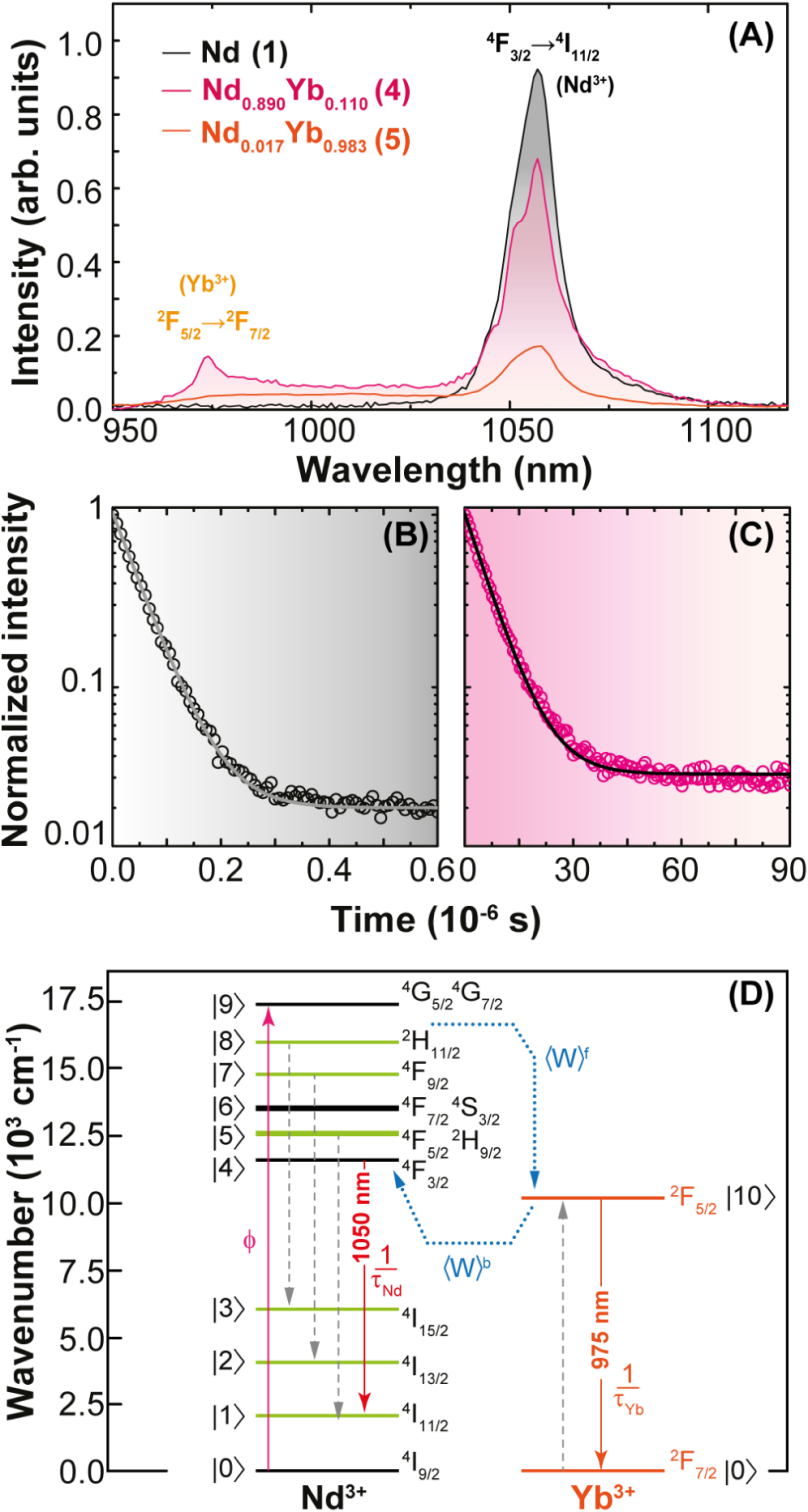
(A) Room temperature emission spectra of **1**, **4**, and **5** upon 580 nm excitation. Temporal dynamics decay traces of **4** upon 801 nm laser excitation monitoring the Nd^III^ and Yb^III^ emitting levels at (B) 1058 nm (^4^F_3/2_) and (C) 975 nm (^2^F_5/2_). (D) Jablonski-type energy level diagram depicting the Nd–Yb energy transfer process. **ϕ** is the pumping rate ^4^I_9/2_ → [^4^G_5/2_,^2^G_7/2_] when Nd^III^ is excited at 580 nm. ***τ*_*Nd*_** and ***τ*_*Yb*_** are the decay lifetimes of the Nd^III^ and Yb^III^ emitting levels, respectively.  and  are the average Nd^III^-to-Yb^III^ forward and backward energy transfer rates, respectively. The dashed straight lines on the Nd^III^ side (involving the green levels) are the main energy transfer pathways that have more contributions to . These rates consider the amount of each ion and their distribution in the compound.

Figure 3A shows the emission spectra of **1**, **4**, and **5**. Compounds **1** and **4** show the typical Nd^III^ emission bands related to the ^4^F_3/2_ → ^4^I_11/2_ transition.^46,62^ In the case of **4**, Yb^III^ is present and the Yb^III 2^F_5/2_ → ^2^F_7/2_ emission appears as a faint band at 980 nm.^46^ However, this band is not present in compound **5** due to (i) the low concentration of Nd^III^ ions once the excitation is at 580 nm and (ii) the presence of a center of inversion at the Ln^III^ site (O_h_ point group symmetry) in the Nd_*x*_Yb_(1–*x*)_(BTC) structure (Figure S13b). The presence of a center of inversion does not affect the magnetic dipole transitions but affects the electric dipole interaction.^63^ Thus, from selection rules on *J* quantum number (|*J* – *J*′| = 0 or 1), it is expected that the Nd^III^^4^F_3/2_ → ^4^I_*J*_ (*J* = 13/2, 11/2, 9/2) emissions present very weak intensities with vibronic and quadrupole^64^ as main interactions. This spectroscopic feature could also be observed from the poor emission intensity and emission quantum yield (Table S5) of **5** regarding the second-order emission peak at 1160 nm (Figure S12) while Figure S10 shows that the second-order peaks are weaker for noncentrosymmetric CPs. The emission quantum yields for direct excitation in Nd^III^ (808 nm) and Yb^III^ (980 nm) are 0.022 ± 0.002% and 0.0030 ± 0.0003% (Table S5).

Among the compounds containing mostly Nd^III^ ions (**2**–**4**), **4** is the most promising system for ratiometric temperature measurement because of its higher emission intensity and emission quantum yield (Table S5), when compared to those of the other samples. Moreover, it presents a larger variation of relative intensities with temperature. The temperature-dependent photoluminescence intensity was studied to establish its potential as a new luminescent thermometer. The thermometric properties of the Nd_*x*_Yb_(1–*x*)_(BTC) compounds were not investigated due to their low emission intensities, as discussed before.

**Figure 4:**
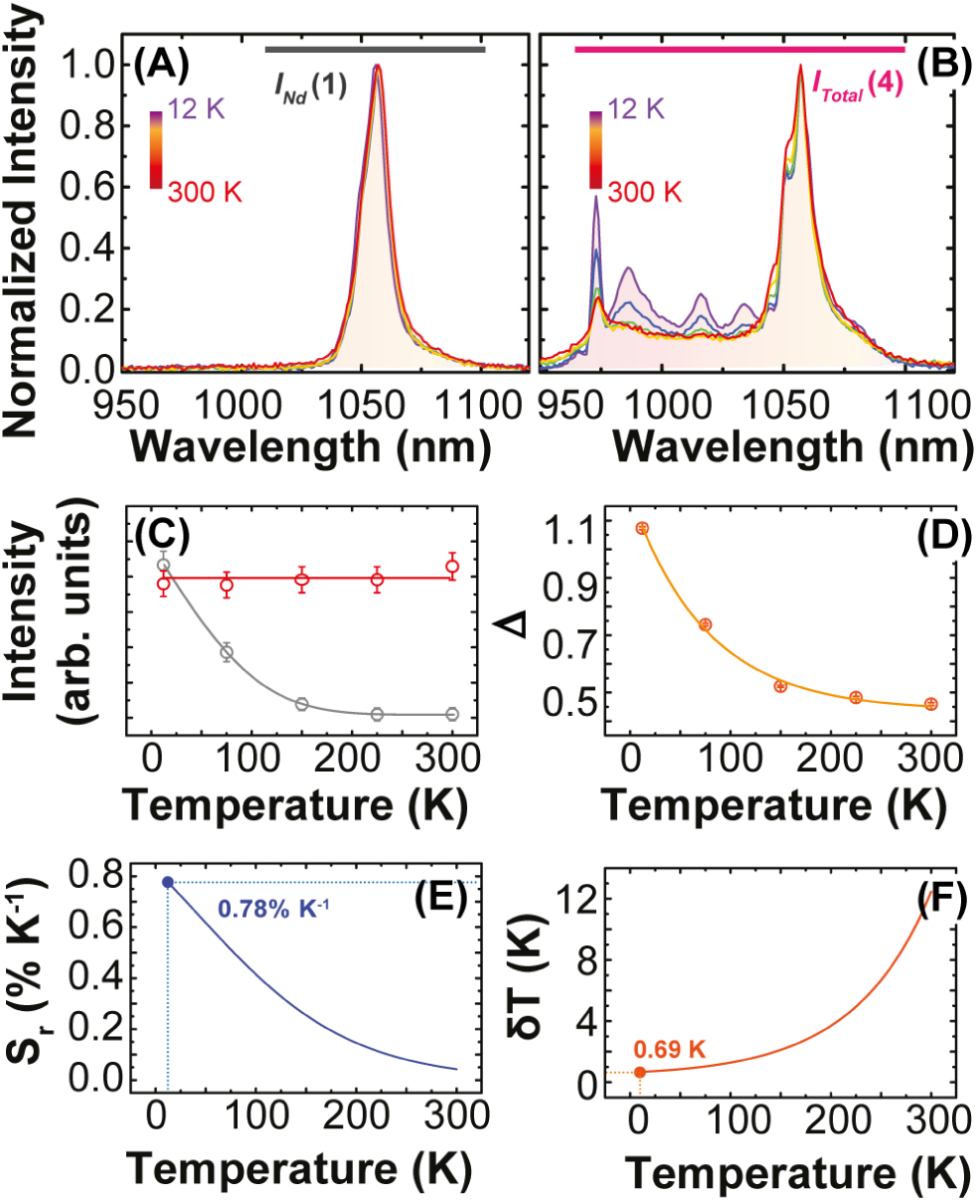
Emission spectra of (A) **1** and (B) **4** recorded in the 12–300 K range under 580 nm excitation. (C) Temperature dependence of *I*_Yb_ (in gray) and *I*_Nd_ (in red) integrate intensities. (D) Thermometric parameter Δ = *I*_Yb_/*I*_Nd_. (E) Relative sensitivity  and (F) temperature uncertainty  for **4**.

Compound **4** shows two bands at 980 and 1058 nm (Figure 3A), attributed to the Yb^III 2^F_5/2_ → ^2^F_7/2_ and Nd^III 4^F_3/2_ → ^4^I_11/2_ transitions, respectively. The relative intensity of Yb^III^ increases when decreasing the temperature and slight changes in the relative intensities between Yb^III^ and Nd^III^ emission bands at 1000 and 1057 nm, respectively, are observed at different temperatures (Figure 4B).

Additionally, at 300 K, the Yb^III^ and Nd^III^ emission lifetimes in **4** are  7.0 ± 0.3 μs and  51 ± 1 ns, respectively, as reported in Figure 3B,C. The short lifetime of Nd^III 4^F_3/2_ can be attributed to a strong electron–phonon interaction^65−67^ and, once it depends on the material, it was also detected in **1** ( 50 ± 1 ns, Figure S11).

This reflects a misapprehension of the usage of eqs 1 and 2 for estimations of experimental energy transfer and efficiencies. Furthermore, with the help of theoretical Nd^III^ → Yb^III^ energy transfer calculations, we will show in the next section that eqs 1 and 2 using the Nd^III 4^F_3/2_ decay lifetimes are no longer valid because the energy transfer pathways involving this level represent less than 1% of the whole Nd^III^ → Yb^III^ energy transfer process. Consequently, this aspect enables the extraction of Yb^III 2^F_5/2_ → ^2^F_7/2_ integrated intensity () by simple subtraction of the whole integrated area from 950 to 1100 nm by Nd^III 4^F_3/2_ → ^4^I_11/2_ in **1** (Figure 4A), as it will be presented in the Luminescence thermometry subsection.

### Nd^III^-to-Yb^II^ Energy Transfer

The energy transfer (ET) rates between a pair of lanthanide ions were calculated considering the Kushida–Malta model,^68,69^ which considers dipole–dipole (), dipole–quadrupole (), quadrupole–quadrupole (), exchange (, and magnetic dipole–magnetic dipole () mechanisms, as defined by eqs S9–S13, respectively.^39,69^ The ET pathways were selected according to energy mismatch conditions (donor–acceptor energy difference, δ, in Table S7) and selection rules on the *J* quantum numbers:

- Electric dipole interactions follows the traditional Judd–Ofelt^70,71^ selection rule:  (with  = 2, 4, and 6);
- Electric quadrupole interactions: ;
- Magnetic dipole interaction:  (except the case of ).

There are no defined selection rules for the isotropic contribution of the exchange mechanism ().^69^

For an illustration of how the above selection rules work, consider pathway 17 in Tables S8–S11, which involves the donor transition ^4^F_3/2_ → ^4^I_11/2_ (Nd^III^) transferring energy to the acceptor transition ^2^F_7/2_ → ^2^F_5/2_ (Yb^III^) (Figure 3D). We can expect contributions from the  mechanism because both transitions obey the electric dipole selection rule. The squared reduced matrix elements ⟨^4^*I*_11/2_∥*U*^(λ)^∥^4^*F*_3/2_⟩^2^ are nonzero for  = 4 and 6 (see Table S12) since  (for this Nd^III^ transition). Similarly, the Yb^III^ transition has contributions from all ⟨^2^*F*_5/2_∥*U*^(λ)^∥^2^*F*_7/2_⟩^2^ because .

However, the dipole–quadrupole mechanism for the same pathway only has the contribution of the first term of eq S10 (donor by electric dipole and acceptor by electric quadrupole) because ⟨^4^*I*_11/2_∥*U*^(2)^∥^4^*F*_3/2_⟩^2^ = 0, annulling the second term (donor by quadrupole and acceptor by dipole). Similarly, the ET rate for the quadrupole–quadrupole mechanism (, eq S11) for this pathway is zero since the selection rule on *J* for the donor transition is not fulfilled ( does not satisfy the condition ).

The sum of all mechanisms for a given pathway  (e.g., Nd^III^ [^4^F_3/2_ → ^4^I_9/2_]  Yb^III^ [^2^F_7/2_ → ^2^F_5/2_]) is expressed as , while the sum of all pathways is defined as the total pairwise energy transfer rate . Tables S8–S11 show the pairwise energy transfer rates for the Nd–Yb distances of 5.85, 7.03, 7.26, and 8.66 Å, respectively. In these tables, each pathway *″p″* (i.e., a calculated ET rate consisting of one donor transition and one acceptor transition) is labeled as , where the superscript letter *″l″* represents the energy transfer direction ( and  stand for forward and backward) and the subscript *″i″* represents the Nd^III^–Yb^III^ distances order from the crystallographic structure ( 1, 2, 3, and 4 for respective of  5.85, 7.03, 7.26, and 8.66 Å).  is the sum of all 64 ET pathways. Thus, as an example,  is the forward Nd^III^ → Yb^III^ energy transfer when Nd^III^–Yb^III^ distance is 5.85 Å.

Concerning the ET pathways with contributions from the Nd^III 4^F_3/2_ level (i.e., ^4^F_3/2_ → ^4^I_J_, pathways  1, 17, 33, and 49 in Tables S8–S11), the sum of them together for each Nd–Yb distance are 410, 95, 75, and 19 s^–1^ which represents 0.7%, 1.0%, 1.0%, and 1.4% of the total ET rate, respectively. This result implies that transitions from Nd^III 4^F_3/2_ level are not important for the case of the Nd^III^–Yb^III^ ET process and, as a result, the usage of eqs 1 and 2, considering the lifetime of this level, is not enough to estimate the experimental rate and efficiency. On the other hand, the most relevant ET pathways are from [^2^H_11/2_ → ^4^I_15/2_], [^4^F_9/2_ → ^4^I_13/2_], and [^2^H_9/2_/^4^F_5/2_ → ^4^I_11/2_] transitions (see pathways 55, 38, and 18/19 in Tables S8–S11), representing respectively around 85%, 7%, and 7% of the total Nd–Yb ET rate. It is worth mentioning that eqs 1, 2 are still valid for other lanthanide pairs in which the main donor level is the emitting one, such as the case of Tb–Eu, where the Tb^III 5^D_4_ level has an important contribution to the energy transfer process.^39,41^ The dominant mechanism governing the energy transfer process is the , with pairwise forward (Nd^III^-to-Yb^III^) ET rates for pathway 55 (Nd^III^ [^2^H_11/2_ → ^4^I_15/2_]  Yb^III^ [^2^F_7/2_ → ^2^F_5/2_] in Tables S8–S11) of 1.8 × 10^5^, 2.8 × 10^4^, 2.0 × 10^4^, and 3.5 × 10^3^ s^–1^ corresponding to Nd^III^–Yb^III^ distances of 5.85, 7.03, 7.26, and 8.66 Å, respectively. The  mechanism is also responsible for the backward ET (Yb^III^-to-Nd^III^) process.

Once the pairwise Nd^III^–Yb^III^ ET rates are calculated (Tables S8–S11), we can simulate a Monte Carlo type distribution of the coordinates of Nd^III^ and Yb^III^ ions in the host matrix by a homemade program written in C language (it can be provided upon request). From the crystallographic data of **1** (Nd(BTC)(H_2_O)_6_ sample), the unit cell (1 × 1 × 1) was expanded to a large one (20 × 20 × 20, Figure S14) with a volume of ≈10378 nm^3^ containing 32000 Nd^III^ host sites, which can be replaced randomly by Yb^III^ until reaching the dopant amount desired (in %). Consequently, the occurrence of Nd–Yb pair as a function of distance  and concentration  of Yb^III^ throughout the matrix can be obtained and, consequently, the average forward  and backward  energy transfer rates:^41,72^
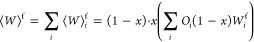
3
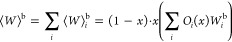
4where, as mentioned before,  stands for the forward and  for the backward energy transfer for the ^th^ Nd–Yb distance (Tables S8–S11). The acceptor Yb^III^ and the donor/host Nd^III^ stoichiometric fractions are represented by  and , respectively. The occurrence coefficients  are related to the formation of a Nd–Yb pair at distance , regarding the acceptor (for forward energy transfer ) or donor (for backward energy transfer ) amount obtained from hundreds of Monte Carlo simulations for each Yb^III^ amount:^41,72^
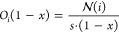

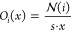
5where  is the counting of Nd–Yb pairs at distance  and  is the number of host sites in the undoped matrix (equals 32000 host sites in the 20 × 20 × 20 expanded cell, Figure S14a). Once the backward energy transfer is related to the energy coming from the Yb^III^ to the Nd^III^ ions, the ‘acceptor’ in this case is Nd^III^ and it justifies the use of the coefficients  instead of  to calculate the . All values of  obtained from simulations as well as  and  are presented in Table S13.

Figure S15a shows the total calculated  with the changing of the Yb^III^ and Nd^III^ stoichiometric fractions while Figure S15b shows only the contribution of the backward Yb^III^ [^2^F_5/2_ → ^2^F_7/2_]  Nd^III^ [^4^I_15/2_ → ^2^H_11/2_] pathway, which is responsible for the quenching of the Yb^III^ emission when temperature increases, as observed in Figure 4B. The backward  is very sensitive to the temperature changes due to the contribution of about 89% of pathway 55 (Nd^III^ [^2^H_11/2_ → ^4^I_15/2_]  Yb^III^ [^2^F_7/2_ → ^2^F_5/2_], Tables S8–S11), which has a close resonant energy mismatch δ (Table S7). Our simulations varied the Yb^III^ amount as given by the synthesized samples (1 – *x* = 0.047; 0.057; 0.110; representing, respectively, the (**3**); (**2**); (**4**); samples) and we also extrapolated until 1 – *x* = 0.200 to see the trend of the ET rates. For values of Yb^III^ doped above this limit, the Nd_*x*_Yb_(1–*x*)_(BTC) structure starts to appear (Figure S2), and once this phase has a Ln^III^ placed at a centrosymmetric site (Figure S13b), the emission of the Ln^III^ is quenched.^64^ This is the reason we limited the simulations up to 1 – *x* = 0.200.

Estimation of the emitting level populations (e.g., Nd^III 4^F_3/2_ and Yb^III 2^F_5/2_) requires an effort to build and solve a set of coupled ordinary differential equations (ODEs) where the main rates are included, such as Ln–Ln energy transfer, radiative rates, and multiphonon relaxations. The transient of one level *P*_*k*_ is represented by solving an 11-level system of rate equations (eqs S19–S29). There are many numerical methods for solving a set of coupled ordinary differential equations;^73^ however, we have been using the Radau method^74^ because it provided fast and accurate results that were in excellent agreement with other Ln-based luminescence processes.^75−81^

### Experimental and Theoretical Luminescence Thermometry

Considering the thermal dependence of this energy transfer from Nd^III^ to Yb^III^, such systems can be exploited as ratiometric thermometers. First, emission of **1** was studied at different temperatures, as reported in Figure S10, showing a relatively weak signal at 1058 nm, assigned to the Nd^III 4^F_9/2_ → ^4^I_11/2_ transition, upon 580 nm excitation. The intensity of the signal is weak considering the excitation of the second order around 1160 nm as a reference and, in this case, no significant changes were observed when decreasing the temperature.

Figure 4A, B displays the temperature dependence from 12 K up to 300 K of **1** and **5** emission spectra upon 580 nm lamp excitation. For both samples, it can be observed that the Nd^III 2^F_3/2_ → ^4^I_11/2_ intensity has little temperature dependence, remaining almost constant (*I*_*Nd*_ in Figure 4C). Thus, the intensity of the Yb^III 2^F_5/2_ → ^2^F_7/2_ (*I*_*Yb*_) transition in sample **4** can be obtained by the subtraction of the integrated intensity *I*_*Nd*_ (highlighted area in Figure 4A) by the integrated intensity *I*_*Total*_ (highlighted area in Figure 4B). This can be assumed because the Nd^III 2^F_3/2_ → ^4^I_11/2_ transition is not predominant in the Nd^III→^ Yb^III^ ET process, as indicated by theoretical calculations.

The distinct temperature dependence of *I*_*Yb*_ and *I*_*Nd*_ indicates that the intensity ratio *I*_*Yb*_/*I*_*Nd*_ is temperature-sensitive and can be used as the thermometric parameter (Δ) for **4**.^6^Figure 4D shows the temperature evolution of Δ. As the temperature increases Δ decreases in the 12 to 220 K range, remaining constant at this temperature until 300 K, meaning that **4** is temperature-sensitive in the cryogenic range up to 220 K, and mostly insensitive beyond 220 K. The thermal performance of **4** was evaluated through the relative thermal sensitivity (), and the temperature uncertainty (), which are the figures of merit to fully characterize the performance of an optical temperature sensor.^5,6,24^

Figure 4E displays *S*_r_ decreasing with temperature from a maximum value of 0.8%·K^–1^ at 12 K, reaching less than 0.1%·K^–1^ from 250 K and beyond, suggesting the temperature operative range of **4** as optical sensors ranges from 12 K up to 220 K, with a minimum  of 0.7 at 12 K (Figure 4F). Despite the scarcity of reports on Nd^III^–Yb^III^ systems for luminescence thermometry in the cryogenic temperature range, the *S*_r_ values presented in this study are comparable to those previously reported (Table S4). Likewise, the main advantage of our approach is the possibility to rationalize this thermometric performance in terms of the underneath energy transfer processes, as discussed in the following section.

The theoretical thermometric parameters of Nd^III^/Yb^III^ mixed CPs were modeled through (i) energy transfer rates between Ln ions,^39,41,69,72^ (ii) Judd–Ofelt intensity parameters,^82,83^ (iii) Miyakawa–Dexter approach for multiphonon decay rates,^84^ and (iv) rate equations modeling.^78,85−88^ These building blocks allow us to estimate the relative emission intensity of Yb^III^ (*I*_Yb_) and Nd^III^ (*I*_Nd_), resulting in the theoretical thermometric parameter Δ = *I*_Yb_/*I*_Nd_.

Figure S17a is presented for illustrative purposes and shows a comparison between the experimental and theoretical Δ values for sample **4**, assuming  ns and  μs (as measured at room temperature, Figure S11) for all temperatures in our simulations. The discrepancies observed at lower temperatures, indicated by red arrows (Figure S17a), suggest that the lifetimes, particularly , should be longer. This observation aligns with the temperature dependence of the electron–phonon coupling. To address these discrepancies, Figure S17b demonstrates the mitigation achieved in the simulations by considering longer lifetimes for both τ parameters in the low-temperature range below 150 K.

Furthermore, utilizing these theoretical curves, we can deduce that for temperatures below 150 K, the behavior of Δ is primarily governed by the Nd^III^–Yb^III^ energy transfer, specifically the backward rates involving the Nd^III 2^H_11/2_ level. On the other hand, at high temperatures, the dominant factor influencing the thermometric parameter is the shortening of τ. This unusual shortening of τ_Nd_ to the order of 50 ns may potentially be attributed to thermally activated phonons coupling with Nd^III 4^F_3/2_, resulting in a fast depopulation of this level.

**Figure 5:**
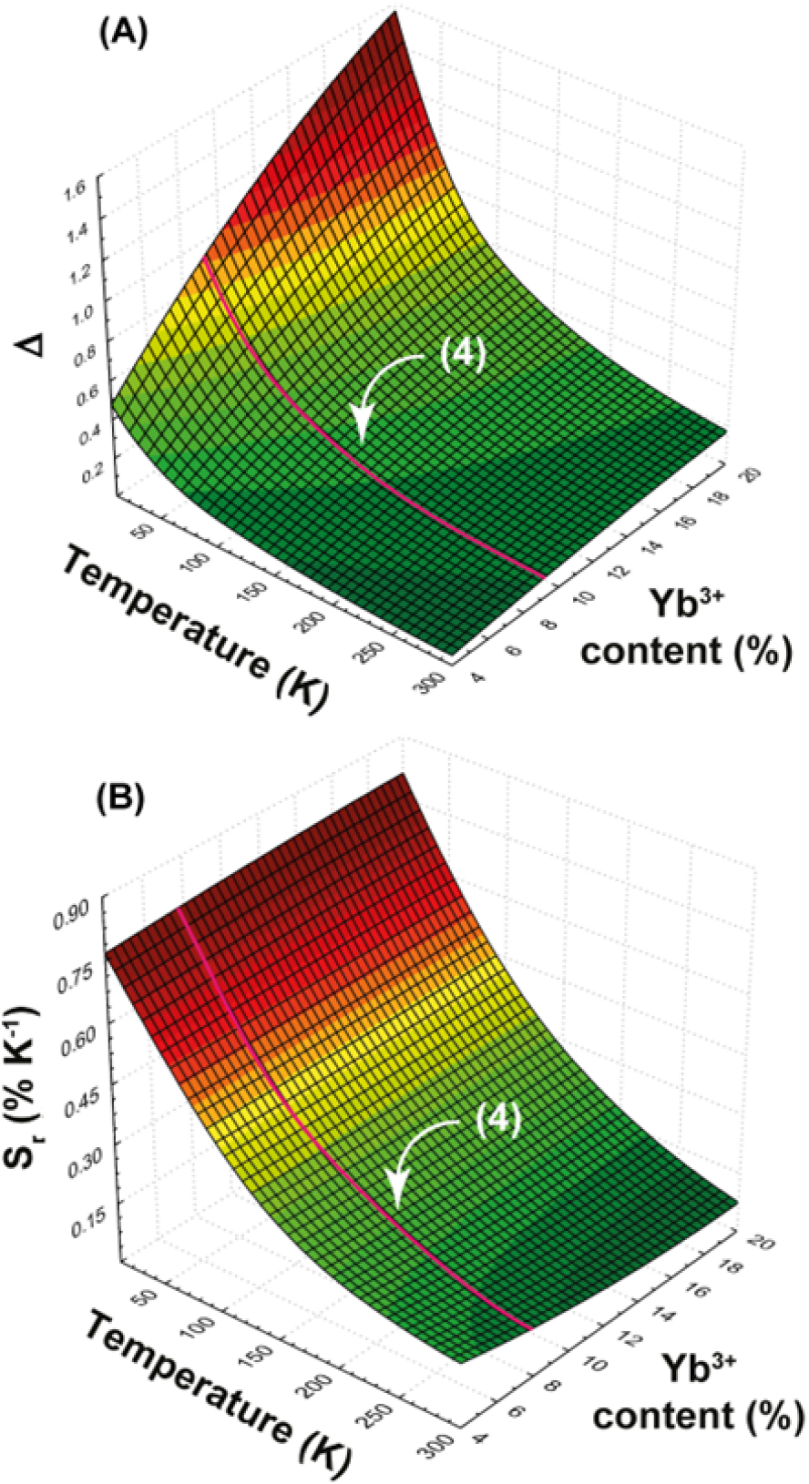
Theoretical (A) thermometric parameter Δ and (B) relative thermal sensitivity *S*_r_ as a function of temperature and Yb^III^ amount. The magenta lines indicate the simulated curves for **4**.

Figure 5 shows the simulated surfaces of Δ and *S*_r_ as a function of Yb^3+^ content and temperature. Comparing the highlighted curve in Figure 5A,B (representing the simulation for sample **4**) with Figure 4D,E, there is a significant agreement between the theoretical and experimental data. This finding supports the earlier discussed point in this article, namely, the limited involvement of the Nd^III 4^F_3/2_ level in the energy transfer process. Consequently, eqs 1 and 2 , which typically describe energy transfer rates and efficiencies between Ln ions, do not apply to Nd–Yb interactions unless the excitation occurs directly in the Nd^III 4^F_3/2_ level, not involving the Nd^III^ levels above.

## Conclusions

Two different classes of CPs, formulated, respectively, as Nd_*x*_Yb_(1–*x*)_(BTC)(H_2_O)_6_ (*x* = 1 (**1**); *x* = 0.943 (**2**); *x* = 0.953 (**3**); *x* = 0.890 (**4**)) and Nd_*x*_Yb_(1–*x*)_(BTC) (*x* = 0.017 (**5**), *x* = 0 (**6**)), were fully characterized by using a multitechnique approach to study their structure, morphology, composition, thermal stability, and optical properties. Particularly, **4** and **5** were selected to perform variable temperature photoluminescence studies in the 10–300 K range, which revealed a decrease of intensity ratio Nd^III^/Yb^III^-related emission upon increasing the temperature. This trend is more evident in **4**, which is therefore the most promising system within the entire series to be employed as a thermometer. The operative range of the **4** luminescent thermometer ranges from 12 up to 220 K, with a minimum  of 0.69 at 12 K. However, rather than emphasizing the thermometric performance of **4**, the objective of the work is to fully understand the underlying energy transfer mechanisms and their crucial implications for optimizing energy transfer-driven ratiometric luminescent thermometers.

Then, theoretical calculations suggested that the Nd–Yb nonradiative energy transfer comes from a different and unexpected pathway for both forward (Nd^III^ [^2^H_11/2_ → ^4^I_15/2_] → Yb^III^ [^2^F_7/2_ → ^2^F_5/2_]) and backward (Yb^III^ [^2^F_5/2_ → ^2^F_7/2_] → Nd^III^ [^4^I_15/2_ → ^2^H_11/2_]) energy transfer. The immediate outcome of these calculations is that eqs 1 and 2 are not always valid to estimate the energy transfer rates and efficiency for Nd–Yb-based materials. Furthermore, the temperature dependency of Nd_*x*_Yb_(1–*x*)_(BTC)(H_2_O)_6_ CPs is strongly influenced by the backward pathway and this corroborates with the observed quenching of the Yb^III^ emission when temperature increases. This is the first time that a complete ET analysis on the Nd–Yb pair was done where simulations of Ln’-to-Ln″ ET-driven thermometers were in good agreement with the experimental data. Remarkably the present joint experimental/theoretical work has the potential to pave the way to a rationalization of NIR luminescent thermometers based on Nd–Yb energy transfer.

## Experimental Section

### Materials

Lanthanide nitrates and the 1,3,5-benzenetricarboxylic acid were purchased from Alfa Aesar and Sigma-Aldrich, and then used without further purification.

### Synthesis

All Nd_*x*_Yb_(1–*x*)_BTC(H_2_O)_6_ (*x* = 1 (**1**); *x* = 0.943 (**2**); *x* = 0.953 (**3**); *x* = 0.890 (**4**)) and Nd_*x*_Yb_(1–*x*)_BTC (*x* = 0.017 (**5**), *x* = 0 (**6**)) compounds were prepared according to the previously reported method^48^ as follows: 0.5 mmol of Ln^III^ precursor (Nd(NO_3_)_3_·6H_2_O for **1** and Yb(NO_3_)_3_·6H_2_O for **6**) was mixed with 0.5 mmol of H_3_BTC (1,3,5-benzenetricarboxylic acid) and ground in an agate mortar for 5 min. Then the mixture was transferred into a 25 mL boron-silicate vial and heated at 130 °C for 24 h. After cooling to room temperature, the powder was collected and washed with distilled water and ethanol two times each and then dried at 60 °C for 3 h. For the synthesis of compounds **2–5**, the procedure was the same but the two Ln^III^ nitrates were mixed in different Nd/Yb stoichiometric ratios: from 95/5 to 80/20 (**2–4**) and 5/95 (**5**). The syntheses here proposed typically provide the final products in 100–300 mg amounts and are highly reproducible. Therefore, using batches from parallel preparations can easily lead to the accumulation of gram-sized samples. The conventional pathway to obtain these materials involves the use of hydrothermal methods. Although ancillary to this work, these recipes are here presented: compounds **1** and **6** were synthesized via a hydrothermal approach. A mixture of Ln(NO_3_)_3_·6(H_2_O) (Ln^III^ = Nd Yb, (0.05 mmol), H_3_BTC (0.15 mmol), NaOH (1,5 mmol), and water (25 mL) was heated at 120 °C for 24 h in a 50 mL Teflon-lined stainless-steel autoclave reactor. After cooling, a white powder, suitable for further characterizations, was obtained.

### ICP-MS

Inductively coupled plasma spectroscopy (ICP) was performed on an Agilent Technologies ICP-MS 7900 spectrometer. The samples were prepared by using microwave digestion in an acid solution (5 mg of sample in 500 μL of concentrated HNO_3_) followed by dilution with water (5 mL final volume).

### Infrared Spectroscopy

FT-IR spectra were collected using a Bruker Equinox 55 spectrometer, with the samples prepared as KBr pellets (Figure S4).

### Brunauer–Emmett–Teller (BET) Analyses

The textural properties were studied by nitrogen adsorption–desorption isotherms at −196 °C, measured on a Micromeritics ASAP 2020 system. The samples were preheated under vacuum at 50 °C (heating rate, 1 °C·min^–1^) for 12 h. BET SSA values found: 37 m^2^·g^–1^ for Nd(BTC)(H_2_O)_6_ and 20 m^2^·g^–1^ for Yb(BTC) (Text S3).

### EDX Microanalysis

SEM images and EDX Microanalysis were performed both on a Hitachi S-4800 and ESEM:FEI Quanta 200 field emission scanning electron microscopes (Tables S2–S3 and Figure S6).

### Thermogravimetric Analysis

Thermogravimetric analysis was performed in alumina crucibles with the instrument STA-6000 under nitrogen flux (40 mL/min) in the 25–800 °C temperature range at 10 °C/min.

### Powder X-Ray Diffraction

PXRD patterns for fingerprinting purposes were collected by using a θ–θ Bragg–Brentano geometry Seifert X 3000 diffractometer equipped with a Cu Kα source (λ = 1.5418 Å), a graphite monochromator on the diffracted beam, and a scintillation counter. Step size 0.05°, acquisition time 2 s/step. Structural PXRD studies required a more careful sample preparation, data collection strategy, and several computational steps (indexing, structure solution, and Rietveld refinement), collectively presented in the SI file. CSD Codes: 2212923–2212924.

### Dynamic Light Scattering (DLS)

The suspensions of Nd/Yb CPs in water/DMSO were prepared by suspending 2 mg of microcrystalline powder in 2 mL of solvent and ultrasonicating it for 15 min. Then they were diluted (200 μL of suspension and 800 μL of solvent) to allow DLS performed with the Malvern ZETASIZER NANO instrument.

### Photophysical Measurements

Continuous-wave diffuse reflectance of crystals of coordination compounds was performed with a dual-beam spectrophotometer equipped with an integrating sphere accessory (Agilent Cary 5000 UV–vis–NIR). Emission and excitation spectra were recorded on a modular double grating excitation spectrofluorimeter with a TRIAX 320 emission monochromator (Fluorolog-3, Horiba Scientific) coupled to an NIR H9170 Hamamatsu photomultiplier, using the front face acquisition mode. The excitation source was a 450 W xenon arc lamp. The excitation spectra were corrected for the spectral distribution of the lamp intensity by using a photodiode reference detector. Time-resolved measurements were carried out with pulsed Xe–Hg lamp excitation in front face acquisition mode. The low-temperature measurements (12 K) were performed using a helium-closed cycle cryostat with a vacuum system measuring ca. 5 × 10^–6^ mbar and a Lakeshore 330 autotuning temperature controller with a resistance heater.

## Theoretical Section

### Nd–Yb Energy Transfer

The pairwise energy transfer rates for Nd–Yb are calculated from eqs S9–S13 according to the procedures described in refs.^39,69^ (see Supporting Information for more details). The distribution of Nd^III^ and Yb^III^ and the average energy transfer rates (from the pairwise Nd–Yb rates) were calculated using the method described in ref.^41^.

### Multiphonon Decay Rates

The nonradiative decay rates between adjacent levels of Nd^III^ ion were calculated using the Miyakawa–Dexter model as described in Supporting Information and ref (84).

### Rate Equations

A general differential rate equation has the general form:^78,85−88^
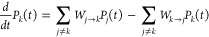
6where the summations run the levels of the system.  and  are the populations of the levels  and ,  and  are rates between these states (Nd^III^–Yb^III^ energy transfer or decay processes). The first summation represents all rates that enter in , while the second represents those that depart from . The complete set of the rate equation model is given by the coupled equations (eqs S19–S29).

### Intensity Parameters and Radiative Rates

The Judd–Ofelt intensity parameters were calculated using the Simple Overlap Model (SOM)^83^ for the odd component of the ligand field and the Bond Overlap Model (BOM)^82^ for the polarizability-dependent term. The radiative rates are calculated using eqs S31–S33 (see Supporting Information for further details).

### Thermometric Parameter and Relative Thermal Sensitivity

The experimental Δ was defined as , where  and  are the integrated intensity of the ^2^F_5/2_ → ^2^F_7/2_ and ^4^F_3/2_ → ^4^I_11/2_ emissions, respectively. Thus, the theoretical Δ is given by
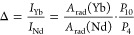
7

where  is the radiative component of the ^2^F_5/2_ → ^2^F_7/2_ and ^4^F_3/2_ → ^4^I_11/2_ transitions.  and  represent the calculated populations of the Yb^III 2^F_5/2_ and Nd^III 4^F_3/2_ emitting levels in the steady-state regime, respectively. The thermal sensitivity is given by
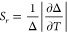
8as defined for the experimental one.

## Supporting Information Available

Powder X-ray diffraction; FT-IR spectra; thermogravimetric analysis; EDX microanalysis, ICP-MS data, and SEM images; photophysical characterization; materials stability by DLS and PXRD measurements; performance of Nd^III^/Yb^III^-based luminescent thermometers; photoluminescent properties; theoretical modeling methodology; supplementary text 1: an estimate of specific surface areas; supplementary text 2: thermal stability in air; supplementary text 3: textural properties (PDF)
crystallographic data of **1** (Nd(BTC)(H_2_O)_6_) (CIF)
crystallographic data of Nd_*x*_Yb_(1–*x*)_(BTC)(H_2_O)_6_ (CIF)

## Supplementary Material

cm4c00362_si_001.pdf

cm4c00362_si_002.cif

cm4c00362_si_003.cif

## Abbreviations

CP: coordination polymer
MOF: metal–organic framework
H_3_BTC: 1,3,5-benzentricarboxylic acid
NIR: near-infrared
ET: energy transfer
PXRD: powder X-ray diffractometry
ICP-MS: induced coupled plasm mass spectrometry
SEM-EDX: scanning electron microscopy—energy dispersive X-ray
TGA: thermal gravimetric analysis
FT-IR: Fourier transformed—infrared spectroscopy
DR: diffuse Reflectance spectra
