# Supplementary material for: Insights into NdIII to YbIII Energy Transfer and Its Implications in Luminescence Thermometry
Source: Chem Mater. 2024 Mar 28;36(7):3452–63. doi: 10.1021/acs.chemmater.4c00362 (PMC11008107; doi:10.1021/acs.chemmater.4c00362)
Supplement: Supplementary file 1 — cm4c00362_si_001.pdf [file cm4c00362_si_001.pdf]

Supporting Information

Insights into Nd^{III} to Yb^{III} Energy Transfer and Its Implications in the Luminescence Thermometry

Mariangela Oggianu,[†,¥,‡] Valentina Mameli,[†,¥,‡] Miguel A. Hernández-Rodríguez,[ξ,‡] Noemi Monni,[†,¥] Manuel Souto,[δ] Carlos D.S. Brites,[ξ] Carla Cannas,[†,¥] Fabio Manna,[†] Francesco Quochi,[ǁ,¥] Enzo Cadoni,[†] Norberto Masciocchi,[§] Albano N. Carneiro Neto,[ξ,*] Luís D. Carlos,[ξ,*] and Maria Laura Mercuri[†,¥,*]

† Dipartimento di Scienze Chimiche e Geologiche, Università degli Studi di Cagliari, I-09042 Monserrato, Italy.

¥ INSTM, Via Giuseppe Giusti, 9, 50121 Firenze, Italy.

ξ Phantom-g, Department of Physics, CICECO-Aveiro Institute of Materials, University of Aveiro, Aveiro 3810-393, Portugal.

δ Department of Chemistry, CICECO-Aveiro Institute of Materials, University of Aveiro, Aveiro 3810-393, Portugal.

ǁ Dipartimento di Fisica, Università degli Studi di Cagliari, Complesso Universitario di Monserrato, I-09042 Monserrato , Italy.

§ Dipartimento di Scienza e Alta Tecnologia & To.Sca.Lab., Universita dell'Insubria, via Valleggio 11, 22100 Como, Italy.

Contents

1. Powder X-Ray Diffraction (PXRD) 2
   1.1. Fingerprinting the CP synthesis 2
      Figure S1 2
   1.2. Fingerprinting the compositional aspects .. 3
      Figure S2 3
   1.3. Structural Powder Diffraction Analysis 3
      Figure S3 4
      Table S1 4
2. FT-IR Spectra 5
      Figure S4 5
3. Thermogravimetric Analysis (TGA) 6
      Figure S5 6
4. EDX Microanalysis, ICP-MS Data, and SEM images 7
      Table S2 7
      Table S3 7
      Figure S6 8
5. Photophysical Characterization 9
      Figure S7 9
6. Materials stability by DLS and PXRD Measurements 10
      Figure S8 10
      Figure S9 11
7. Performance of Nd^{III}/Yb^{III}-based luminescent thermometers 12
      Table S4 12
8. Photoluminescent properties 13
      Figure S10 13
      Figure S11 13
      Figure S12 13
   8.1. Emission quantum yield 14
      Table S5 14
9. Theoretical modeling methodology 15
   9.1. Theoretical intensity parameters 15
      Figure S13 16
      Table S6 16
   9.2. Multiphonon decay rates 17
   9.3. Pairwise Nd–Yb energy transfer rates 18
      Table S7 20
      Table S8 21
      Table S9 23
      Table S10 25
      Table S11 27
   9.4. Matrix elements calculations 29
      Table S12 31
   9.5. Simulations of the Nd–Yb distribution and the average energy transfer rates 33
      Figure S14 33
      Table S13 34
      Figure S15 34
   9.6. Rate equations modeling 35
      Figure S16 36
   9.7. Radiative rates, intensities, and thermometric parameter 37
      Figure S17 38
10. Supplementary Text 1: An Estimate of Specific Surface Areas 39
      Table S14 39
11. Supplementary Text 2: Thermal Stability in Air. 40
      Figure S18 40
      Figure S19 41
      Table S15 41
12. Supplementary Text 3: Textural Properties. 42
      Figure S20 42
13. References 43

1. Powder X-Ray Diffraction (PXRD)

1.1. Fingerprinting the CP synthesis

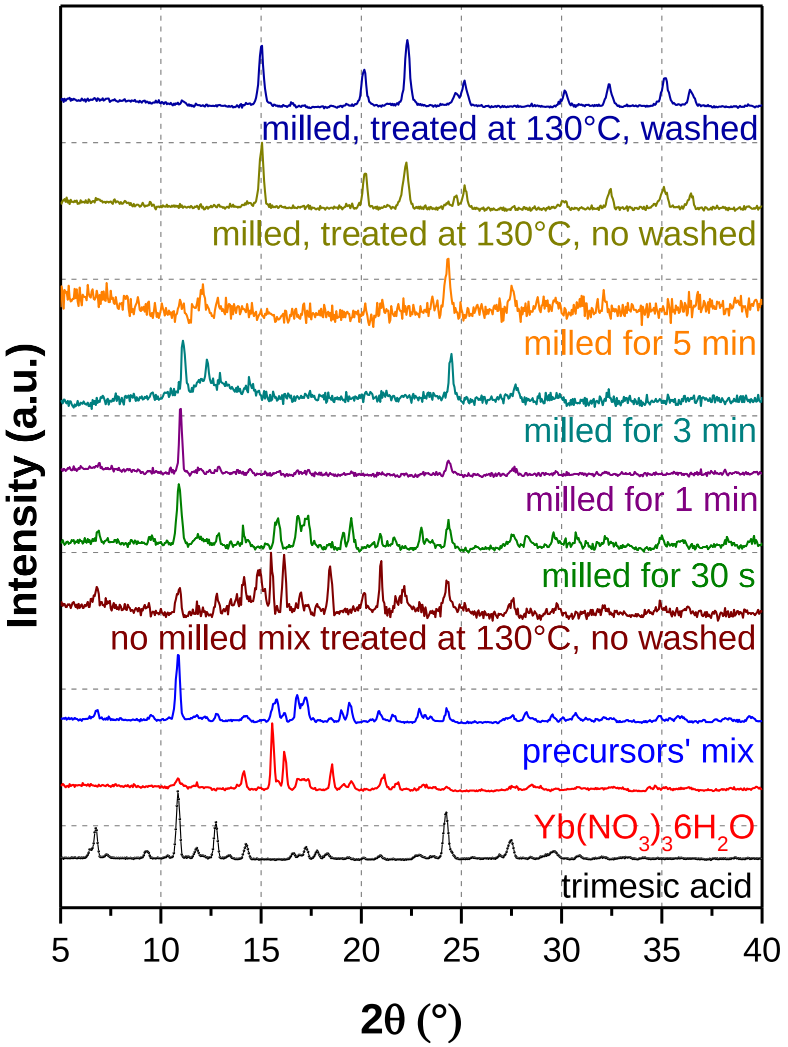

Figure S1. PXRD pattern of samples at different stages of the reaction leading to the Yb(BTC) (**6**).

1.2. Fingerprinting the compositional aspects

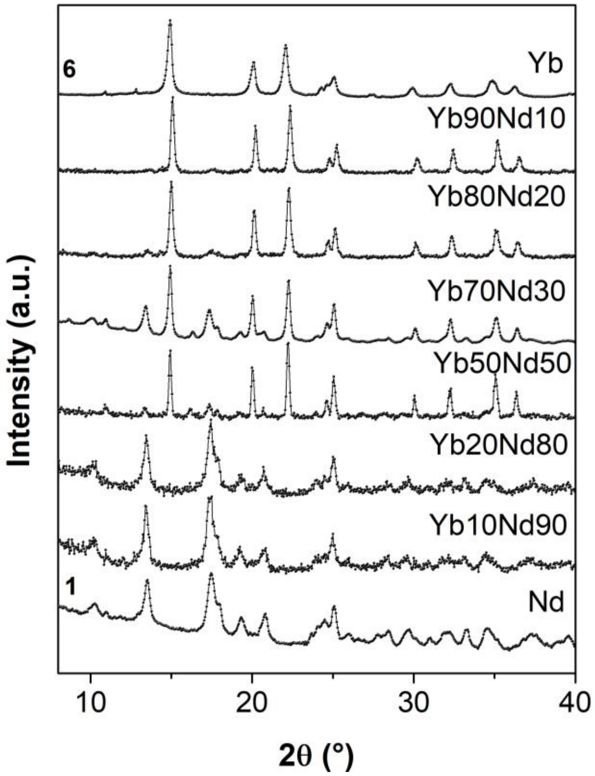

Figure S2. PXRD patterns of samples with different stoichiometric ratios Ln':Ln'' ratios in the Ln precursors.

1.3. Structural Powder Diffraction Analysis

PXRD measurements for structure solution were performed using a Bruker AXS D8 Advance diffractometer in Bragg-Brentano θ:θ geometry, equipped with a Lynxeye position sensitive detector. DS: 0.5°; Generator setting: 40 kV, 40 mA; Ni-filtered Cu-Kα radiation, λ = 1.5418 Å. Data were collected in the 3–105° 2θ range, sampling at 0.02°, with scan time lasting approximately 16h. PXRD structure solution of compounds **1** and **6** was performed using the TOPAS-R software.[1] Standard peak search methods followed by profile fitting allowed the accurate estimate of the low-angle peak position. These values, through the SVD indexing algorithm,[2] provided a C-centered monoclinic cell for **1** [a = 11.44, b = 17.93, c = 7.25 Å, b = 119.4°, GOF(24) = 64.0] and a rhombohedral one for **6** [a = 8.87, c = 18.73 Å, GOF(20) = 35.9]. Space group determination through the analysis of systematic absences, and the possible isomorphous character of **1** with RAVJUV [catena-((μ_3-Benzene-1,3,5-tricarboxylato)-hexa-aqua-gadolinium][3] and of **6** with CETMEU [catena-((μ_6-cyclohexane-1,3,5-tricarboxylato)-erbium)],[4] indicated, for the two phases, *Cc* and R–3c, respectively, later confirmed by successful structure refinement. The structural models were taken from the isomorphous structures, with the BTC ligand in **1** modeled by a partially flexible rigid body and water molecules bound to Nd geometrically restrained. The final refinements were eventually carried out by the Rietveld method. The background was modelled by a polynomial function of the Chebyshev type, peak profiles were described by the Fundamental Parameters Approach[5] and a common

(refinable) isotropic thermal factor was attributed to all atoms. **Figure S3** contains the final Rietveld refinement plots for **1** and **6**.

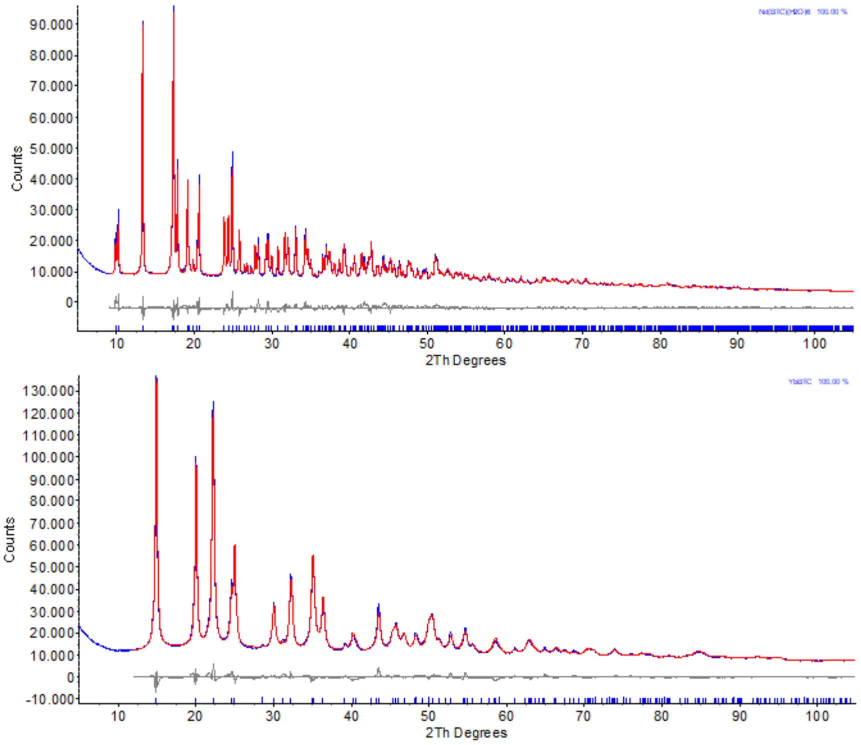

Figure S3. Final Rietveld refinement plots for **1** (top) and **6** (bottom); difference plot and peak markers at the bottom.

Table S1. Nd–O bond distances in **1**. Nd–O bonds were refined using a cost function which includes restrained geometrical parameters. The asymmetric unit of **1** is shown on the right.

Bond	Distance (Å)
Nd–O1 (BTC)	2.39
Nd–O2 (H_2O)	2.54
Nd–O3 (H_2O)	2.48
Nd–O4(BTC)	2.40
Nd–O5 (H_2O)	2.54
Nd–O6 (H_2O)	2.43
Nd–O7 (BTC)	2.47
Nd–O8 (H_2O)	2.52
Nd–O9 (H_2O)	2.45

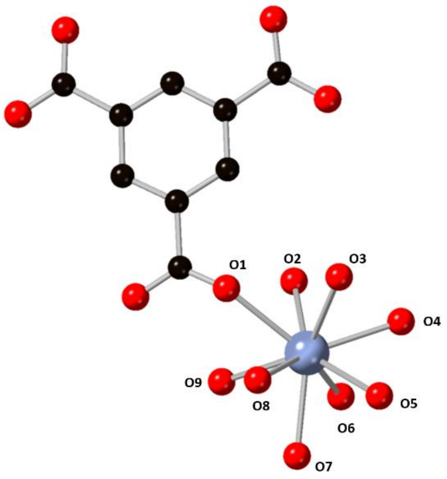

2. FT-IR Spectra

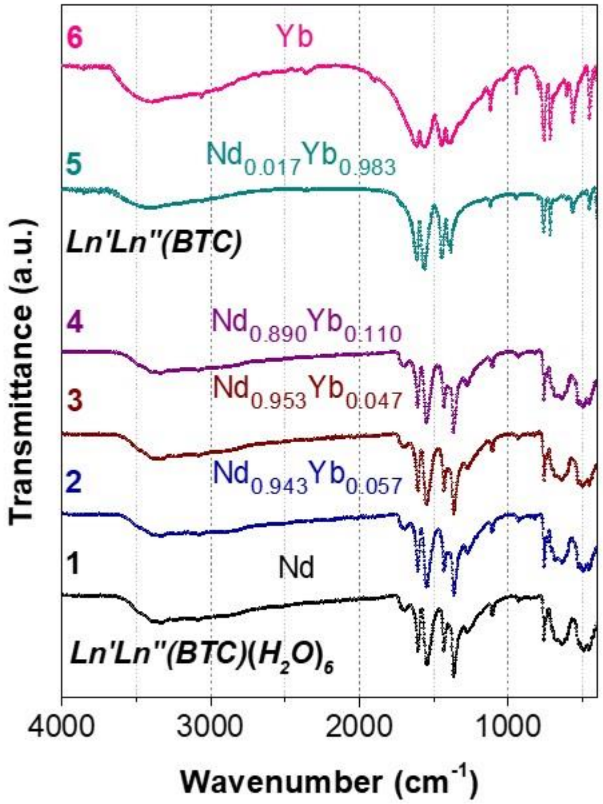

Figure S4. FT-IR spectra in the 4000-400 cm^{-1} range of the **1-6** CPs.

3. Thermogravimetric Analysis (TGA)

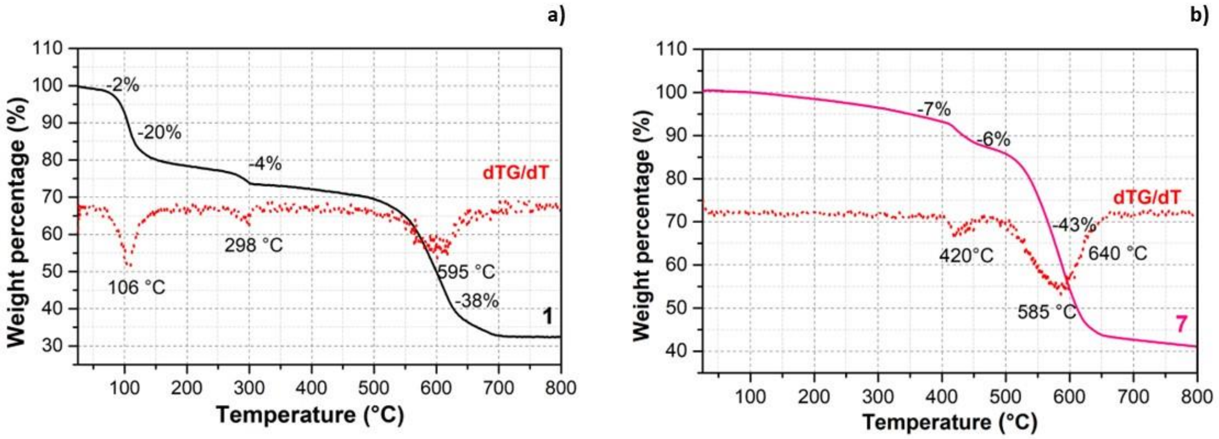

Figure S5. Thermogram of (a) **1** and (b) **6** in the 25–800°C range. The percentages shown in the graph are the corresponding weight loss.

4. EDX Microanalysis, ICP-MS Data, and SEM images

Table S2. Semiquantitative Nd/Yb ratio of **1-6**, obtained by EDX measurements and quantitative Nd/Yb ratio provided by ICP-MS.

Sample	Nd/Yb EDX	Nd/Yb formula
1	100% Nd	Nd BTC $(H_2O)_6$
2	95.1/4.9	$Nd_{0.943}Yb_{0.057}BTC\ (H_2O)_6$
3	96/4	$Nd_{0.953}Yb_{0.047}BTC\ (H_2O)_6$
4	88.4/11.6	$Nd_{0.890}Yb_{0.110}\ BTC\ (H_2O)_6$
5	1.53/98.4	$Nd_{0.017}Yb_{0.983}\ BTC$
6	100% Yb	Yb (BTC)

Table S3. Microanalysis and ICP-MS data of **2-5** samples showing the Nd/Yb atomic ratio (with three repetitions per sample).

Sample	Nd (at %)	Yb (at %)
2	93.21	6.79
	96.06	3.94
	96.17	3.83
3	94.99	5.01
	96.49	3.51
	96.49	3.51
4	87.56	12.44
	87.94	12.06
	89.78	10.22
5	0.20	99.80
	0.11	99.89
	1.5	98.5

Morphological Characterization: Scanning Electron Microscopy (SEM) images of **1-6** are reported in **Figure S6**. Compound **1** shows flower-like micrometric aggregates of about 4-14 μm in size formed by needles with diameter in the 50-100 nm range. Such morphology is consistent with the ribbon crystal structure, and it was shared by all hydrated phases, **1**-**4** CPs. At variance, compounds **5** and **6** show a clear granular morphology, where grains are apparently made up of micro or nanometric platelets with a very broad size distribution. The different shape of the secondary and primary particles in **1-6** CPs proves a structure-morphology correlation (**Figure S6**).

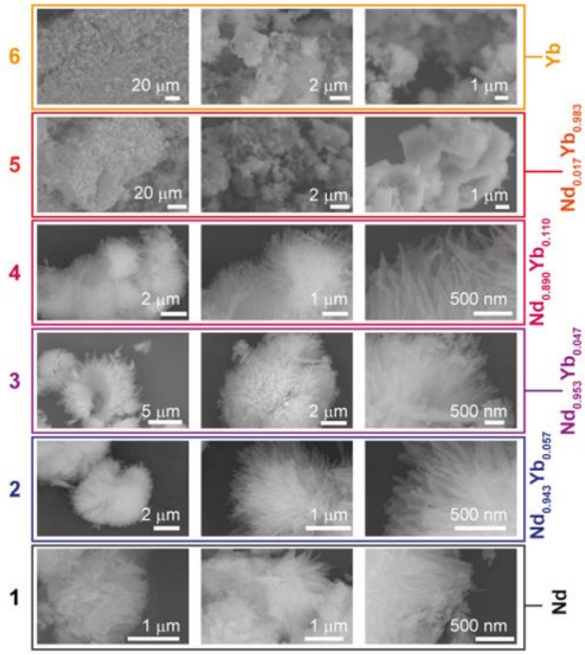

Figure S6. SEM images of 1-6 at different magnifications.

5. Photophysical Characterization

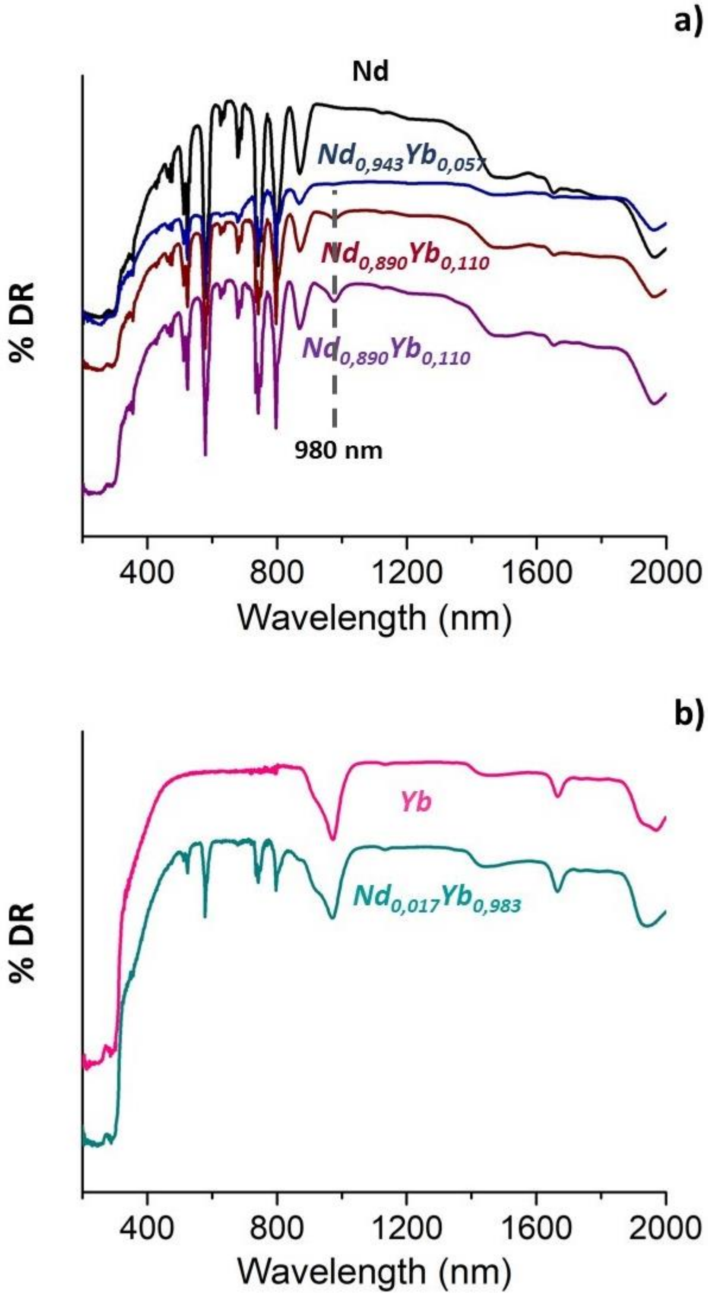

Figure S7. Diffuse Reflectance spectra of **a) 1-4** and **b) 5, 6**, in 200-2000 nm wavelength range.

6. Materials stability by DLS and PXRD Measurements

The stability in water or DMSO of both **4** and **5** have been investigated firstly by Dynamic Light Scattering (DLS) and then by PXRD measurements.

DLS was performed on **4** and **5** suspended in both water or DMSO. The suspensions of Nd/Yb CPs in water/DMSO were prepared by suspending 2 mg of microcrystalline powder in 2 mL of solvent and ultrasonicated for 15 min. Then were diluted (200 µL of suspension and 800 µL of solvents) to allow DLS performed with Malvern ZETASIZER NANO instrument.

All the four milky white suspensions were diluted 1:4 to perform the measurements. 4 is stable in DMSO with a size distribution centered at 396 nm (**Figure S8a**) whereas it decomposes in water, its mean size decreasing over a time of few minutes. On the contrary, 5 shows to be more stable in water suspension (**Figure S8b**) with a mean diameter of 450 nm, while in DMSO the size decreases over few minutes due to the high instability of the suspension.

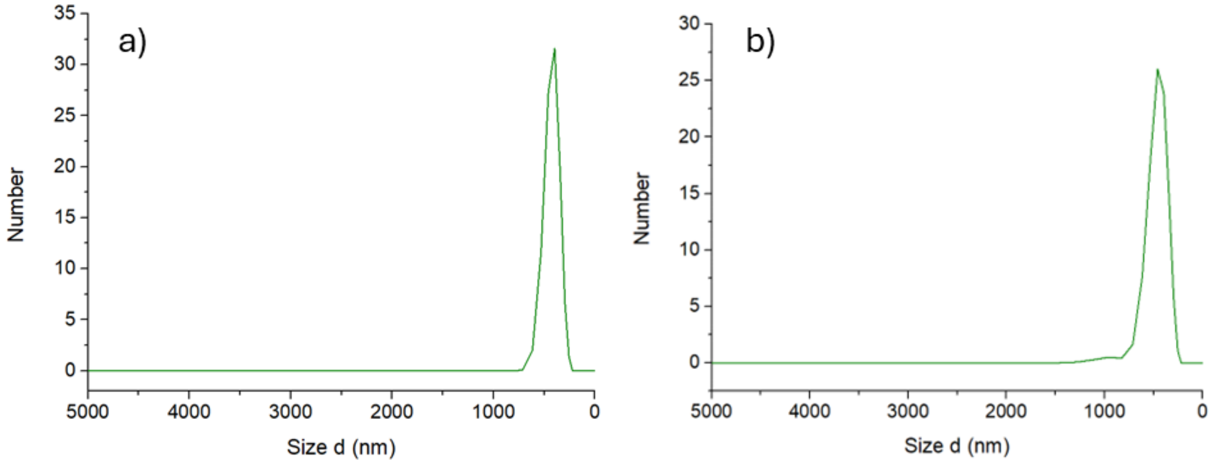

Figure S8. Hydrodynamic diameter distribution of suspension of (a) **4** in DMSO and (b) **5** in water, each one were averaged over three measurements.

Compound **4 (**in DMSO) and compound **5** (suspended in water) demonstrate stability for a period of 1-2 hours. However, beyond this timeframe, the formation of a white precipitate can be observed at the bottom of the vials.

To verify if the pristine crystal structure was retained, PXRD was performed on **4** and **5** after prolonged soaking in DMSO and water, respectively. Each sample was suspended in each solvent for 2 hours and then deposited on a zero-background sample holder for PXRD analysis. Both samples show excellent crystal-phase stability after soaking, as shown in **Figure S9**. In **4** the excess scattering below 13° is likely due to residual (not completely evaporated) DMSO.

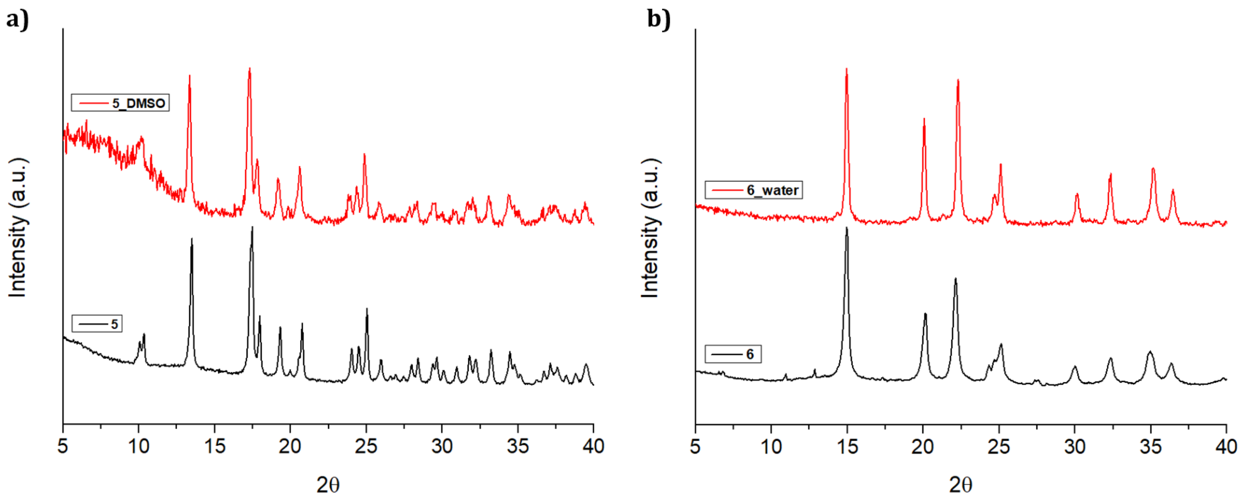

Figure S9. PXRD patterns in 5-40° 2θ range of **4** (**a**) and **5** (**b**) after soaking in DMSO and water, respectively.

7. Performance of Nd^{III}/Yb^{III}-based luminescent thermometers

Table S4. Summary of the performance parameters of Nd^{III}/Yb^{III}-based luminescent thermometers. The temperature operating range (ΔT) and maximum relative thermal sensitivity (S_m) are presented.

**Material**	**ΔT (K)**	**S_m (%·K^{-1})**	**Ref.**
$Yb^{III}Nd^{III}Gd^{III}$ $(ant)_3$	83-293	1.8	[6]
$Yb^{III}Nd^{III}Gd^{III}$ $(acr)_3(H_2O)_8$	83-393	0.2	[6]
BaY_2O_4:Yb^{III}/Nd^{III}	298-548	3.2	[7]
$LaPO_4$:Yb^{III}/Nd^{III}	280-490	1.7	[8]
La_2O_3:Yb^{III}/Nd^{III}	293-1233	1.6	[9]
$CaWO_4$:Yb^{III}/Nd^{III}	303-733	2.7	[10]
$Bi_4Ti_3O_{12}$:Yb^{III}/Nd^{III}	297-420	1.5	[11]
$NaYF_4$:Yb^{III}/Nd^{III}	297-420	2.4	[12]
$Yb^{III}Nd^{III}$-MOF	15-300	0.1	[13]
$Yb^{III}Nd^{III}(BTC)(H_2O)_6$	12-300	0.8	This work

8. Photoluminescent properties

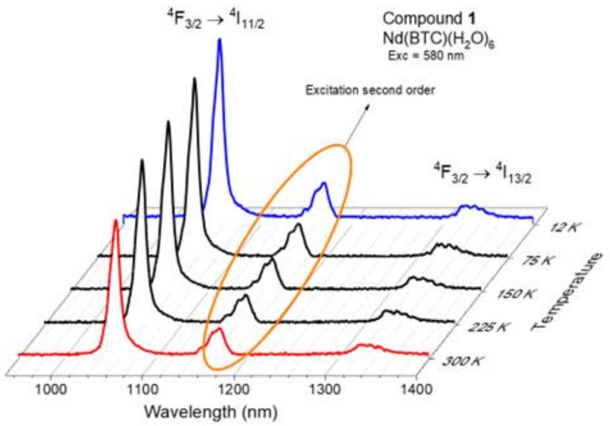

Figure S10. Emission spectra of **1**, upon 580 nm excitation, in 12–300 K range temperature. The spectral range between 1125 and 1200 nm was omitted.

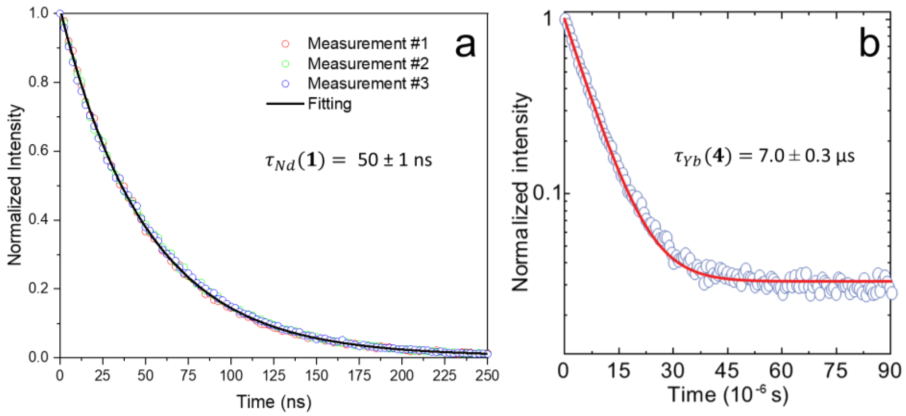

Figure S11. Luminescence decay curves at room temperature obtained for a) sample **1**, monitoring the Nd^{III} $^4F_{3/2}$ level, and b) sample **4**, monitoring the Yb^{III} $^2F_{5/2}$ level, under excitation at 801 nm.

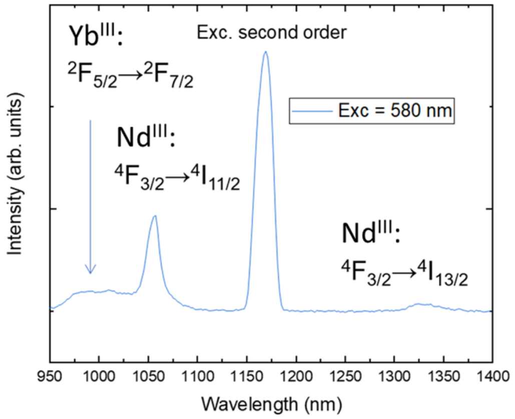

Figure S12. Emission spectrum of **5** excited at 580 nm in the range of 950 – 1400 nm.

8.1. Emission quantum yield

Absolute photoluminescence quantum yields were measured with a Quantaurus-QY (C13534, Hamamatsu) system equipped with an integrating sphere as the sample chamber and two multi-channel analyzers for signal detection in the visible and in the NIR spectral ranges. Two external laser diodes peaking at 808 nm (FC-808 5W, CNI Lasers) and 980 nm (FC-980 5W, CNI Lasers) were used as excitation sources. For both lasers, the power is adjusted corresponding to 380±10 $W \cdot cm^{-2}$. All measurements were performed on pellets placed on a quartz petri plate sample holder. As a reference sample, the empty sample petri plate was used. The photoluminescence quantum yield was calculated by the quotient between the emitted and absorbed photons by the sample. The equipment's software computes the photoluminescence quantum yield using the user-defined wavelength integration ranges for excitation and emission. The emission integration range was 950-1200 nm, for 808 nm excitation, and 1000-1200 nm, for 980 nm. Three measurements were performed for each sample at each power density value and the mean value is reported. According to the manufacturer, each measurement presents a relative systemic error of 10%.

Table S5. Emission quantum yield values measured at room temperature, using λ_x = 808 or 980 nm excitation wavelengths.

**Sample**	**Emission Quantum yield (%)**	
	**λ_x=808 nm**	**λ_x=980 nm**
1	0.015±0.002	--
2	0.015±0.002	0.002±0.0002
3	0.016±0.002	0.0020±0.0002
4	0.022±0.002	0.0030±0.0003
5	0.012±0.001	0.0018±0.0002

9. Theoretical modeling methodology

In the following subsections, details of the modeling process are presented and most of the theoretical procedure is based on the Jablonski-type energy level diagram in Figure 3D and the crystallographic structure of the $Nd(BTC)_3(H_2O)_6$. Each Ln^{III} level (or group of levels) is labeled as $|\boldsymbol{N}\rangle$ in the calculations of multiphonon rates and population fraction.

The pumping rate ϕ (in s^{-1}) was estimated using the relation,

$$\phi = \frac{\sigma \rho \lambda_{exc}}{hc} \quad \text{(S1)}$$

where σ (~10^{-20} cm^2) is the typical absorption cross-section of the Ln^{III} ions,[14,15] ρ (in $W\cdot cm^{-2}$) is the power density of the excitation source with λ_{exc} (in nm) as wavelength. h is the planck constant (in erg·s) and c is the speed of light (in $cm\cdot s^{-1}$). No unit conversion is necessary if those indicated in parentheses are used.

9.1. *Theoretical intensity parameters*

The forced electric dipole (FED – Judd-Ofelt theory) and dynamic coupling (DC) are the dominant mechanisms for the 4*f*-4*f* intensities when the lanthanide occupies a non-centrosymmetric site.[16–18] The theoretical expressions here used for the intensity parameters, Ω_λ, have been described in detail in several references.[19–21] However, to evaluate the energy transfer involving the dipole interaction (dipole-dipole and dipole-quadrupole mechanisms), only the FED contribution should be considered.[22,23]

$$\Omega_\lambda = (2\lambda+1)\sum_{t,p}\frac{|B_{\lambda tp}|^2}{2t+1} \quad , \qquad B_{\lambda tp} = B_{\lambda tp}^{FED} + B_{\lambda tp}^{DC} \quad \text{(S2)}$$

where,

$$B_{\lambda tp}^{FED} = \frac{2}{\Delta E}\langle r^{t+1}\rangle \Theta(t,\lambda)\left(\frac{4\pi}{2t+1}\right)^{\frac{1}{2}}\sum_j \frac{e^2\rho_j g_j (2\beta_j)^{t+1}}{R_j^{t+1}}\left(Y_p^{t*}\right)_j \quad \text{(S3)}$$

$$B_{\lambda tp}^{DC} = -\left[\frac{(\lambda+1)(2\lambda+3)}{(2\lambda+1)}\right]^{\frac{1}{2}}\langle r^\lambda\rangle\langle f\|C^{(\lambda)}\|f\rangle\left(\frac{4\pi}{2t+1}\right)^{\frac{1}{2}}\delta_{t,\lambda+1}\times\sum_j\frac{\left[(2\beta_j)^{t+1}\alpha_{OP,j}+\alpha'_j\right]}{R_j^{t+1}}\left(Y_p^{t*}\right)_j \quad \text{(S4)}$$

being *t* and *p* the ranks and components of the complex conjugate of the spherical harmonics $\left(Y_p^{t*}\right)$. The ρ is the overlap integral between the valence subshells of the ligating atom and the 4*f* subshell of the lanthanide ion. For the case of Ln^{III}–O chemical bonds, ρ was obtained using the parametric approach as described in Ref. [24]. $\beta = 1/(1 \pm \rho)$ is a parameter

that defines the centroid of the electronic density of the chemical bond Ln–X (X= ligating atom) and g is known as charge factor, which the product $\rho e g$ represents the electronic charge shared of the Ln–X chemical bond. α' is the effective polarizability of the ligand and α_{OP} is the Ln–X chemical bond polarizability. Eq. S3 is the expression of the Simple Overlap Model (SOM)[25,26] for the FED mechanism while Eq. S4 represents the Bond Overlap Model (BOM)[20,27] for the DC mechanism. See references [20,21,24–28] for further details on the quantities in Eqs. S3 and S4.

The coordination geometries (Figure S13) were extracted from the crystallographic structure and inserted as a input parameter in the JOYSpectra program [29] to obtain the values of Ω_λ. The related parameters (g, ρ, α', α_{OP}, and R) for both Nd^{III} and Yb^{III} in compounds **1-5** are presented in Table S6. The Ln^{III} ions in compounds **6** and **7** are placed in a centrosymmetric symmetry and this leads to very weak emissions.

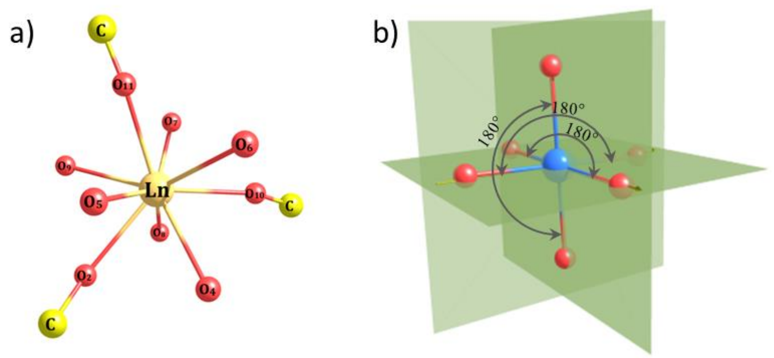

Figure S13. Coordination geometry extracted from the crystallographic structure for a) **1** and b) **7**. a) The oxygen atoms bonded with carbon represent the oxygen atoms belonging to the BTC ligand while the others are regarding the water molecules. b) Shows that the Yb^{III} is at O_h point group symmetry.

Table S6. Ln–O bond distance R (in Å), charge factor g (dimensionless), ρ (dimensionless), polarizabilities α' (in Å^3) and α_{OP} (in 10^{-3} Å^3) used for the calculation of Ω_λ and their FED contributions (both in units of 10^{-20} cm^2) for Nd^{III} and Yb^{III} coordination geometries in compounds **1–5**. The atoms label follows the same presented in Figure S13a.

Ligating atom	R	g	α'	ρ (Nd–O)	ρ (Yb–O)	α_{OP} (Nd–O)	α_{OP} (Yb–O)
**O_2 (BTC)**	2.466	0.300	0.200	0.042	0.065	2.015	6.137
**O_4 (H_2O)**	2.448	0.500	0.100	0.043	0.067	2.087	6.342
**O_5 (H_2O)**	2.430	0.500	0.100	0.044	0.069	2.157	6.537
**O_6 (H_2O)**	2.540	0.500	0.100	0.038	0.058	1.750	5.337
**O_7 (H_2O)**	2.478	0.500	0.100	0.041	0.064	1.970	6.005
**O_8 (H_2O)**	2.521	0.500	0.100	0.039	0.059	1.813	5.534
**O_9 (H_2O)**	2.537	0.500	0.100	0.038	0.058	1.758	5.363
**O_{10} (BTC)**	2.389	0.300	0.200	0.047	0.074	2.332	7.006
**O_{11} (BTC)**	2.400	0.300	0.200	0.046	0.072	2.282	6.876
**Ln^{III}**	Ω_2	Ω_2(FED)	Ω_4	Ω_4(FED)	Ω_6	Ω_6(FED)	
**Nd^{III}**	2.832	0.069	8.057	0.796	1.305	1.130	
**Yb^{III}**	2.671	0.005	2.519	0.015	0.063	0.019	

9.2. *Multiphonon decay rates*

The multiphonon decay can be estimated using the energy gap law [30–33],

$$W = W_0 e^{-(\alpha \cdot \Delta E)} \tag{S5}$$

where ΔE is the energy difference (in cm^{-1}) between adjacent J levels ($|N+1\rangle \longrightarrow |N\rangle$ in Figure 3D), W_0 (in s^{-1}) is the decay rate extrapolated to zero energy gap ($\Delta E \approx 0$). The α (in cm) depends on the material, particularly, the mean phonon energy $\hbar\varpi$.

According to Miyakawa-Dexter model,[34] this factor can be estimated by:

$$\alpha = \frac{1}{\hbar\varpi}\left[\ln\left(\frac{N}{S(n+1)}\right) - 1\right] \tag{S6}$$

where N $(= \Delta E/\hbar\varpi)$ is the number of generated phonons, S is the Huang-Rhys factor whose typical values are in the range of 0.02 to 0.10 for trivalent lanthanides ions [35–37] and n is the phonon occupancy number, given by the Bose-Einstein distribution:

$$n = \frac{1}{e^{\frac{\hbar\varpi}{k_B T}} - 1} \tag{S7}$$

We made numerical simulations setting the Huang-Rhys factor $S = 0.06$ and the mean phonon energy $\hbar\varpi = 500$ cm^{-1} as parameters. Also, $W_0 = 10^8$ s^{-1} was considered, once it is a usually reported value for resonant one-phonon transitions in Ln-doped compounds.[34,38] These simulations were performed for each Nd^{III} $|N+1\rangle \rightarrow |N\rangle$ multiphonon decay (Figure 3D). In addition, the non-radiative reabsorption $|N+1\rangle \leftarrow |N\rangle$ rates were taken into account using Boltzmann statistics,

$$W_{|N+1\rangle \leftarrow |N\rangle} = W_{|N+1\rangle \rightarrow |N\rangle} e^{-\left(\frac{\Delta E}{k_B T}\right)} \tag{S8}$$

where k_B is the Boltzmann constant and T the temperature in K.

It is important to mention that the energy gap law is reasonable for the case of low energy phonons, mainly acoustic phonons with energy within the order of hundreds of cm^{-1}.[39] However, in the case of high energy optical phonons (*e.g.*, $\hbar\varpi = 1600$ cm^{-1}), efficient coupling of a localized specific mode with the $^{(2S+1)}L_J$ level could happen and lead to very fast non-radiative decay rates, quenching the population of this level and, consequently, its emission lines. In other words, when the decay rate by a multiphonon process involving a certain Nd^{III} level is faster than the Nd-Yb energy transfer (departing from the same level), the Yb^{III} emission could barely be observed. Based on this and on the fact that the Nd^{III} $^4F_{3/2}$ level has an uncommon measured short lifetime ($\tau_{Nd} \approx 50$ ns), we believe that there are strong optical phonons

modes with a relevant density of states coupling with the $^4F_{3/2}$ level. This is considered, in an explicit way, by the inclusion of the τ_{Nd} in the rate equations model (subsection *9.6 Rate equations modeling*).

There are reported in the literature Nd^{III} $^4F_{3/2}$ lifetimes ranging from few ms [40,41] while the most common values are in the order of hundreds μs.[42–44] As far as we know, the present work reports the shortest measured lifetime of the Nd^{III} $^4F_{3/2}$ level. Furthermore, our theoretical calculations pointed out that this level is not important as thought in the energy transfer process between Nd^{III} and Yb^{III} and, consequently, an important insight regarding the well-known formulae using the experimental lifetimes to obtain the energy transfer rates and efficiencies is given thanks to this unexpected short lifetime.

9.3. *Pairwise Nd–Yb energy transfer rates*

The energy transfer rates between a pair of lanthanide ions can be calculated considering the dipole–dipole (W_{d-d}), dipole–quadrupole (W_{d-q}), quadrupole–quadrupole (W_{q-q}), and exchange (W_{ex}) mechanisms, and magnetic dipole–magnetic dipole (W_{md-md}) mechanisms:[22,23]

$$W_{d-d} = \frac{(1-\sigma_1^D)^2(1-\sigma_1^A)^2}{(2J_D^*+1)(2J_A+1)}\frac{4\pi}{3\hbar}\frac{e^4}{R^6}\left(\sum_\lambda \Omega_\lambda^D \langle\psi_D J_D\|U^{(\lambda)}\|\psi_D^* J_D^*\rangle^2\right) \times\left(\sum_\lambda \Omega_\lambda^A \langle\psi_A^* J_A^*\|U^{(\lambda)}\|\psi_A J_A\rangle^2\right)F \tag{S9}$$

$$W_{d-q} = \frac{\left(1-\sigma_1^{D,A}\right)^2\left(1-\sigma_2^{A,D}\right)^2}{(2J_D^*+1)(2J_A+1)}\frac{\pi}{\hbar}\frac{e^4}{R^8}\langle f\|C^{(2)}\|f\rangle^2 \times\left[\left(\sum_\lambda \Omega_\lambda^D \langle\psi_D J_D\|U^{(\lambda)}\|\psi_D^* J_D^*\rangle^2\right)\langle r^2\rangle_A^2\langle\psi_A^* J_A^*\|U^{(2)}\|\psi_A J_A\rangle^2 + \left(\sum_\lambda \Omega_\lambda^A \langle\psi_A^* J_A^*\|U^{(\lambda)}\|\psi_A J_A\rangle^2\right)\langle r^2\rangle_D^2\langle\psi_D^* J_D^*\|U^{(2)}\|\psi_D J_D\rangle^2\right]F \tag{S10}$$

$$W_{q-q} = \frac{(1-\sigma_2^D)^2\left(1-\sigma_2^A\right)^2}{(2J_D^*+1)(2J_A+1)}\frac{28\pi}{5\hbar}\frac{e^4}{R^{10}}\langle r^2\rangle_D^2\langle r^2\rangle_A^2\langle f\|C^{(2)}\|f\rangle^4\langle\psi_D J_D\|U^{(2)}\|\psi_D^* J_D^*\rangle^2 \times\langle\psi_A^* J_A^*\|U^{(2)}\|\psi_A J_A\rangle^2 F \tag{S11}$$

$$W_{ex} = \frac{2\pi}{\hbar}\left[\left(\frac{e^2}{R}\right)\rho_{f-f}^2\right]^2 F \tag{S12}$$

$$W_{md-md} = \frac{(1-\sigma_1^D)^2(1-\sigma_1^A)^2}{(2J_D^*+1)(2J_A+1)}\frac{4\pi}{3\hbar}\frac{{\mu_B}^4}{R^6}\langle\psi_D J_D\|L+g_S S\|\psi_D^* J_D^*\rangle^2\langle\psi_A^* J_A^*\|L+g_S S\|\psi_A J_A\rangle^2 F \tag{S13}$$

where the letters A and D stands for the acceptor and donor ions. The intensity parameters Ω_λ are calculated using only the forced electric dipole (FED) mechanism (Eqs. S2 and S3), since in Kushida's expressions the appearance of the Ω_λ parameters is due to opposite parity configuration mixing, as treated in the original Judd-Ofelt theory.[45] The W_{q-q}, W_{ex}, and W_{md-md} mechanisms are independent of the Ω_λ parameters. ρ_{f-f} (Eq. S12) is the overlap integral between the 4*f* subshells of the donor (D) and acceptor (A) lanthanide ions. The integral values as a function of the Nd–Yb distance (R) were calculated using DFT (ADF,[46] BP86 functional[47,48] / TZ2P atomic orbitals basis[49] and the inclusion of Zero-Order Regular Approximation (ZORA) to scalar relativistic effects[50–52]). The values of ρ_{f-f} can be found in reference [24].

Non-radiative energy transfer involving magnetic dipole interactions in inorganic solids was treated in the early 1950's by Dexter [53], and in the 1990's these interactions were applied to energy transfer between lanthanide ions in inorganic crystals by Tanner *et al.* [54–57]. Eq. S13 is based on the recently development in Ref. [22]. The matrix elements $\langle\psi J\|L+g_S S\|\psi^* J^*\rangle$ for the Nd^{III} ion were calculated using intermediate coupling wavefunctions from Rajnak [58] (see subsection *9.4 Matrix elements calculations*) and $\mu_B \left(=\frac{e\hbar}{2m_e c}\right)$ is the Bohr magneton.

The values of $\langle\psi J\|U^{(\lambda)}\|\psi^* J^*\rangle^2$ were taken from Ref. [59]. $\langle r^\lambda\rangle$ are 4*f* radial integrals.[60] The shielding factors $(1-\sigma_k)$ for donor and acceptor (with $k = 1$ and 2) are given by:[61]

$$\left(1-\sigma_k^{D,A}\right) = \rho(2\beta)^{k+1} \tag{S14}$$

where ρ is the overlap integral between the valence subshells of the ligating atom and the 4*f* subshell of the lanthanide ion (also can be found in reference [24]) and $\beta = 1/(1\pm\rho)$.

It can be noted that in all equations regarding the pairwise energy transfer (Eqs. S9–S13) are directly proportional to the factor F. This factor represents the spectral overlap factor that expresses the energy mismatch conditions which contains a sum over Franck–Condon factors. In the case of energy transfer between two lanthanide ions the following analytical expression for F has been used:[23]

$$F = \frac{\ln(2)}{\sqrt{\pi}}\frac{1}{\hbar^2\gamma_D\gamma_A}\left\{\left[\left(\frac{1}{\hbar\gamma_D}\right)^2+\left(\frac{1}{\hbar\gamma_A}\right)^2\right]\ln(2)\right\}^{-\frac{1}{2}} \times \exp\left[\frac{1}{4}\frac{\left(\frac{2\delta}{(\hbar\gamma_D)^2}\ln 2\right)^2}{\left[\left(\frac{1}{\hbar\gamma_A}\right)^2+\left(\frac{1}{\hbar\gamma_D}\right)^2\right]\ln 2}-\left(\frac{\delta}{\hbar\gamma_D}\right)^2\ln(2)\right] \tag{S15}$$

where $\hbar\gamma_D$ and $\hbar\gamma_A$ correspond to the bandwidths at half-height of the donor and acceptor transitions, respectively. Once Ln^{III} ions present narrow transitions, we have been considering $\gamma_D = \gamma_A = 400$ cm^{-1} in our calculations, typically the order of the Yb^{III} $^2F_{7/2} \rightarrow {}^2F_{5/2}$ transition.[23] δ is the energy difference between donor and acceptor transitions, $\delta = E_D - E_A$. The value of F (Eq. S15) for a given pathway should be multiplied by the energy barrier factor $\exp(\delta/k_B T)$ if δ is negative, where k_B is the Boltzmann constant and T is the temperature. Table S7 shows the values of δ for 64 Nd–Yb energy transfer pathways while Tables S8-S11 show the calculated energy transfer rates for different Nd–Yb distances ($R(i)$ = 5.85, 7.03, 7.26, and 8.66 Å, respectively) at 300 K.

Table S7. Values of δ (in units of cm^{-1}) for all Nd-Yb energy transfer pathways. The pathways are labeled with a sequence of numbers with respect to Nd^{III} transitions. All forward pathways involve the non-radiative decay of the Nd^{III} transitions to the absorption of Yb^{III} $^2F_{7/2}\rightarrow{}^2F_{5/2}$.

	$^4I_{9/2}$		$^4I_{11/2}$		$^4I_{13/2}$		$^4I_{15/2}$	
from	pathway	δ	pathway	δ	pathway	δ	pathway	δ
$^4F_{3/2}\rightarrow$	1	1215	17	−664	33	−2648	49	−4698
$^4F_{5/2}\rightarrow$	2	2254	18	375	34	−1609	50	−3659
$^2H_{9/2}\rightarrow$	3	2362	19	483	35	−1501	51	−3551
$^4F_{7/2}\rightarrow$	4	3213	20	1334	36	−650	52	−2700
$^4S_{3/2}\rightarrow$	5	3285	21	1406	37	−578	53	−2628
$^4F_{9/2}\rightarrow$	6	4493	22	2614	38	630	54	−1420
$^2H_{11/2}\rightarrow$	7	5699	23	3820	39	1836	55	214
$^4G_{5/2}\rightarrow$	8	7022	24	5143	40	3159	56	1109
$^2G_{7/2}\rightarrow$	9	7063	25	5184	41	3200	57	1150
$^4G_{7/2}\rightarrow$	10	8887	26	7008	42	5024	58	2974
$^4G_{9/2}\rightarrow$	11	9303	27	7424	43	5440	59	3390
$^2K_{13/2}\rightarrow$	12	9379	28	7500	44	5516	60	3466
$^2D_{3/2}\rightarrow$	13	11019	29	9140	45	7156	61	5106
$^4G_{11/2}\rightarrow$	14	11308	30	9429	46	7445	62	5395
$^2K_{15/2}\rightarrow$	15	11374	31	9495	47	7511	63	5461
$^2P_{1/2}\rightarrow$	16	13052	32	11173	48	9189	64	7139

Table S8. Pairwise nonradiative energy transfer rates (s^{-1}) from Nd^{III} to Yb^{III} at the distance order 1, R=5.85 Å) at 300 K. All pathways involve the Yb^{III} $^2F_{7/2} \rightarrow {}^2F_{5/2}$ transition.

Path. (p)	Nd^{III} transition	$\mathbf{W_{d-d}}$	$\mathbf{W_{d-q}}$	$\mathbf{W_{q-q}}$	$\mathbf{W_{ex}}$	$\mathbf{W_{md-md}}$	$\boldsymbol{\omega_1^f(p)}$	$\boldsymbol{\omega_1^b(p)}$
1	$^4F_{3/2}\to{}^4I_{9/2}$	8.5×10^{-2}	2.8×10^{1}	0.0	7.8×10^{-12}	0.0	2.8×10^{1}	4.4×10^{-2}
2	$^4F_{5/2}\to{}^4I_{9/2}$	6.0×10^{-5}	2.0×10^{-2}	3.0×10^{-2}	3.2×10^{-15}	0.0	5.0×10^{-2}	8.1×10^{-7}
3	$^2H_{9/2}\to{}^4I_{9/2}$	2.7×10^{-6}	9.4×10^{-4}	9.8×10^{-2}	1.1×10^{-15}	7.8×10^{-6}	9.8×10^{-2}	1.6×10^{-6}
4	$^4F_{7/2}\to{}^4I_{9/2}$	4.3×10^{-10}	1.4×10^{-7}	4.9×10^{-7}	3.7×10^{-20}	1.3×10^{-12}	6.3×10^{-7}	1.4×10^{-13}
5	$^4S_{3/2}\to{}^4I_{9/2}$	1.6×10^{-10}	5.3×10^{-8}	0.0	1.4×10^{-20}	0.0	5.3×10^{-8}	4.1×10^{-15}
6	$^4F_{9/2}\to{}^4I_{9/2}$	1.9×10^{-20}	6.2×10^{-18}	1.7×10^{-16}	2.0×10^{-29}	3.0×10^{-20}	1.7×10^{-16}	1.0×10^{-25}
7	$^2H_{11/2}\to{}^4I_{9/2}$	1.1×10^{-32}	3.6×10^{-30}	0.0	5.4×10^{-41}	1.3×10^{-33}	3.6×10^{-30}	7.9×10^{-42}
8	$^4G_{5/2}\to{}^4I_{9/2}$	1.0×10^{-46}	7.9×10^{-44}	1.1×10^{-40}	7.9×10^{-57}	0.0	1.1×10^{-40}	2.1×10^{-55}
9	$^2G_{7/2}\to{}^4I_{9/2}$	8.7×10^{-48}	3.6×10^{-45}	1.9×10^{-42}	2.3×10^{-57}	0.0	1.9×10^{-42}	3.9×10^{-57}
10	$^4G_{7/2}\to{}^4I_{9/2}$	4.4×10^{-75}	1.7×10^{-72}	6.8×10^{-70}	9.7×10^{-85}	1.7×10^{-77}	6.9×10^{-70}	2.3×10^{-88}
11	$^4G_{9/2}\to{}^4I_{9/2}$	1.2×10^{-82}	4.0×10^{-80}	3.1×10^{-78}	7.4×10^{-92}	3.0×10^{-83}	3.1×10^{-78}	1.7×10^{-97}
12	$^2K_{13/2}\to{}^4I_{9/2}$	1.6×10^{-84}	6.1×10^{-82}	1.6×10^{-79}	3.4×10^{-93}	0.0	1.6×10^{-79}	8.9×10^{-99}
13	$^2D_{3/2}\to{}^4I_{9/2}$	8.3×10^{-116}	2.7×10^{-113}	0.0	1.2×10^{-124}	0.0	2.7×10^{-113}	1.6×10^{-136}
14	$^4G_{11/2}\to{}^4I_{9/2}$	1.9×10^{-122}	6.3×10^{-120}	0.0	9.8×10^{-131}	1.4×10^{-124}	6.3×10^{-120}	2.8×10^{-143}
15	$^2K_{15/2}\to{}^4I_{9/2}$	8.9×10^{-124}	2.9×10^{-121}	0.0	3.8×10^{-132}	0.0	2.9×10^{-121}	1.3×10^{-144}
16	$^2P_{1/2}\to{}^4I_{9/2}$	3.0×10^{-161}	9.8×10^{-159}	0.0	1.1×10^{-170}	0.0	9.8×10^{-159}	1.7×10^{-186}
17	$^4F_{3/2}\to{}^4I_{11/2}$	1.9	6.2×10^{2}	0.0	7.4×10^{-11}	0.0	2.6×10^{1}	2.8×10^{2}
18	$^4F_{5/2}\to{}^4I_{11/2}$	7.4×10^{-1}	2.4×10^{2}	0.0	1.4×10^{-10}	0.0	2.4×10^{2}	2.7×10^{1}
19	$^2H_{9/2}\to{}^4I_{11/2}$	6.0×10^{-2}	2.1×10^{1}	3.1×10^{3}	1.2×10^{-10}	8.3×10^{-1}	3.1×10^{3}	3.4×10^{2}
20	$^4F_{7/2}\to{}^4I_{11/2}$	4.8×10^{-2}	1.6×10^{1}	4.3×10^{1}	4.1×10^{-12}	0.0	5.9×10^{1}	8.8×10^{-2}
21	$^4S_{3/2}\to{}^4I_{11/2}$	2.8×10^{-2}	9.2	0.0	2.7×10^{-12}	0.0	9.2	4.8×10^{-3}
22	$^4F_{9/2}\to{}^4I_{11/2}$	5.6×10^{-7}	1.9×10^{-4}	6.8×10^{-5}	7.2×10^{-17}	1.2×10^{-7}	2.5×10^{-4}	1.0×10^{-9}
23	$^2H_{11/2}\to{}^4I_{11/2}$	7.8×10^{-16}	3.1×10^{-13}	1.2×10^{-10}	3.6×10^{-24}	6.9×10^{-15}	1.2×10^{-10}	1.8×10^{-18}
24	$^4G_{5/2}\to{}^4I_{11/2}$	2.5×10^{-25}	8.3×10^{-23}	0.0	2.5×10^{-35}	0.0	8.3×10^{-23}	1.1×10^{-33}
25	$^2G_{7/2}\to{}^4I_{11/2}$	5.1×10^{-26}	3.6×10^{-23}	4.8×10^{-20}	1.0×10^{-35}	0.0	4.8×10^{-20}	6.7×10^{-31}
26	$^4G_{7/2}\to{}^4I_{11/2}$	3.8×10^{-47}	5.1×10^{-44}	9.6×10^{-41}	1.2×10^{-56}	0.0	9.6×10^{-41}	2.2×10^{-55}
27	$^4G_{9/2}\to{}^4I_{11/2}$	1.7×10^{-52}	7.0×10^{-50}	3.6×10^{-47}	2.7×10^{-62}	9.5×10^{-54}	3.6×10^{-47}	1.4×10^{-62}
28	$^2K_{13/2}\to{}^4I_{11/2}$	0.0	0.0	0.0	2.3×10^{-63}	5.9×10^{-54}	5.9×10^{-54}	2.2×10^{-69}
29	$^2D_{3/2}\to{}^4I_{11/2}$	8.4×10^{-80}	2.8×10^{-77}	0.0	5.0×10^{-89}	0.0	2.8×10^{-77}	1.1×10^{-96}
30	$^4G_{11/2}\to{}^4I_{11/2}$	0.0	0.0	0.0	4.4×10^{-94}	3.9×10^{-87}	3.9×10^{-87}	1.2×10^{-106}
31	$^2K_{15/2}\to{}^4I_{11/2}$	1.6×10^{-87}	6.6×10^{-85}	3.5×10^{-82}	3.0×10^{-95}	0.0	3.5×10^{-82}	1.0×10^{-101}
32	$^2P_{1/2}\to{}^4I_{11/2}$	0.0	0.0	0.0	7.0×10^{-128}	0.0	7.0×10^{-128}	3.8×10^{-151}
33	$^4F_{3/2}\to{}^4I_{13/2}$	5.1×10^{-7}	1.7×10^{-4}	0.0	4.9×10^{-17}	0.0	5.1×10^{-10}	6.4×10^{-5}
34	$^4F_{5/2}\to{}^4I_{13/2}$	1.2×10^{-2}	4.1	0.0	7.0×10^{-13}	0.0	1.8×10^{-3}	2.3
35	$^2H_{9/2}\to{}^4I_{13/2}$	3.8×10^{-3}	1.5	5.4×10^{2}	1.5×10^{-12}	0.0	4.0×10^{-1}	5.1×10^{2}
36	$^4F_{7/2}\to{}^4I_{13/2}$	4.5×10^{-1}	1.5×10^{2}	0.0	7.7×10^{-11}	0.0	6.6	1.1×10^{2}
37	$^4S_{3/2}\to{}^4I_{13/2}$	1.5	5.1×10^{2}	0.0	9.3×10^{-11}	0.0	3.2×10^{1}	1.9×10^{2}
38	$^4F_{9/2}\to{}^4I_{13/2}$	1.1	3.6×10^{2}	2.2×10^{3}	8.1×10^{-11}	0.0	2.6×10^{3}	1.2×10^{2}
39	$^2H_{11/2}\to{}^4I_{13/2}$	3.2×10^{-5}	1.2×10^{-2}	4.4	1.3×10^{-13}	8.9×10^{-4}	4.4	7.6×10^{-4}
40	$^4G_{5/2}\to{}^4I_{13/2}$	1.9×10^{-10}	6.3×10^{-8}	0.0	7.9×10^{-20}	0.0	6.3×10^{-8}	9.5×10^{-15}
41	$^2G_{7/2}\to{}^4I_{13/2}$	1.1×10^{-10}	3.5×10^{-8}	0.0	4.5×10^{-20}	0.0	3.6×10^{-8}	5.9×10^{-15}
42	$^4G_{7/2}\to{}^4I_{13/2}$	2.0×10^{-24}	6.6×10^{-22}	0.0	3.5×10^{-34}	0.0	6.6×10^{-22}	1.7×10^{-32}
43	$^4G_{9/2}\to{}^4I_{13/2}$	1.9×10^{-28}	1.7×10^{-25}	2.5×10^{-22}	2.8×10^{-38}	0.0	2.5×10^{-22}	1.1×10^{-33}
44	$^2K_{13/2}\to{}^4I_{13/2}$	1.7×10^{-31}	9.8×10^{-29}	9.9×10^{-26}	4.6×10^{-39}	9.3×10^{-30}	9.9×10^{-26}	4.3×10^{-37}
45	$^2D_{3/2}\to{}^4I_{13/2}$	2.2×10^{-49}	7.2×10^{-47}	0.0	1.3×10^{-58}	0.0	7.2×10^{-47}	3.4×10^{-62}
46	$^4G_{11/2}\to{}^4I_{13/2}$	9.6×10^{-53}	3.7×10^{-50}	1.4×10^{-47}	1.4×10^{-62}	2.7×10^{-54}	1.4×10^{-47}	5.0×10^{-63}

47	$^2K_{15/2}\rightarrow{}^4I_{13/2}$	3.6×10^{-55}	1.2×10^{-52}	2.9×10^{-51}	1.6×10^{-63}	3.8×10^{-54}	3.0×10^{-51}	1.0×10^{-66}
48	$^2P_{1/2}\rightarrow{}^4I_{13/2}$	0.0	0.0	0.0	7.1×10^{-90}	0.0	7.1×10^{-90}	5.1×10^{-109}
49	$^4F_{3/2}\rightarrow{}^4I_{15/2}$	4.6×10^{-22}	1.5×10^{-19}	0.0	3.3×10^{-31}	0.0	2.5×10^{-29}	5.1×10^{-20}
50	$^4F_{5/2}\rightarrow{}^4I_{15/2}$	3.7×10^{-13}	1.2×10^{-10}	0.0	4.9×10^{-23}	0.0	3.0×10^{-18}	6.2×10^{-11}
51	$^2H_{9/2}\rightarrow{}^4I_{15/2}$	1.2×10^{-12}	4.0×10^{-10}	0.0	2.6×10^{-22}	0.0	1.6×10^{-17}	3.3×10^{-10}
52	$^4F_{7/2}\rightarrow{}^4I_{15/2}$	4.8×10^{-7}	1.6×10^{-4}	0.0	2.7×10^{-17}	0.0	3.8×10^{-10}	1.1×10^{-4}
53	$^4S_{3/2}\rightarrow{}^4I_{15/2}$	1.0×10^{-6}	3.3×10^{-4}	0.0	6.1×10^{-17}	0.0	1.1×10^{-9}	1.1×10^{-4}
54	$^4F_{9/2}\rightarrow{}^4I_{15/2}$	4.0×10^{-2}	1.3×10^{1}	0.0	2.4×10^{-12}	0.0	1.4×10^{-2}	1.1×10^{1}
55	$^2H_{11/2}\rightarrow{}^4I_{15/2}$	1.6×10^{-1}	1.3×10^{2}	1.8×10^{5}	1.7×10^{-10}	0.0	1.8×10^{5}	6.4×10^{4}
56	$^4G_{5/2}\rightarrow{}^4I_{15/2}$	2.1×10^{-3}	6.8×10^{-1}	0.0	1.3×10^{-11}	0.0	6.8×10^{-1}	1.7×10^{-3}
57	$^2G_{7/2}\rightarrow{}^4I_{15/2}$	2.9×10^{-2}	9.7	0.0	1.1×10^{-11}	0.0	9.7	2.6×10^{-2}
58	$^4G_{7/2}\rightarrow{}^4I_{15/2}$	5.4×10^{-10}	1.8×10^{-7}	0.0	9.2×10^{-19}	0.0	1.8×10^{-7}	7.7×10^{-14}
59	$^4G_{9/2}\rightarrow{}^4I_{15/2}$	2.0×10^{-11}	6.7×10^{-9}	0.0	3.0×10^{-21}	0.0	6.7×10^{-9}	4.8×10^{-16}
60	$^2K_{13/2}\rightarrow{}^4I_{15/2}$	9.6×10^{-14}	3.2×10^{-11}	0.0	9.6×10^{-22}	1.9×10^{-14}	3.2×10^{-11}	2.2×10^{-18}
61	$^2D_{3/2}\rightarrow{}^4I_{15/2}$	2.4×10^{-26}	7.8×10^{-24}	0.0	5.7×10^{-35}	0.0	7.9×10^{-24}	6.1×10^{-35}
62	$^4G_{11/2}\rightarrow{}^4I_{15/2}$	1.2×10^{-27}	7.8×10^{-25}	9.7×10^{-22}	8.0×10^{-38}	0.0	9.7×10^{-22}	5.6×10^{-33}
63	$^2K_{15/2}\rightarrow{}^4I_{15/2}$	5.0×10^{-30}	2.1×10^{-27}	1.1×10^{-24}	1.7×10^{-38}	8.8×10^{-29}	1.1×10^{-24}	5.9×10^{-36}
64	$^2P_{1/2}\rightarrow{}^4I_{15/2}$	0.0	0.0	0.0	2.2×10^{-58}	0.0	2.2×10^{-58}	2.9×10^{-73}
					$W_1^f = \sum_{p=1}^{64} \omega_1^f(p) =$		$1.8\times10^{5}\ s^{-1}$	
					$W_1^b = \sum_{p=1}^{64} \omega_1^b(p) =$		$6.5\times10^{4}\ s^{-1}$	

Table S9. Pairwise nonradiative energy transfer rates (s^{-1}) from Nd^{III} to Yb^{III} at the distance order 2 (R= 7.03 Å) at 300 K. All pathways involve the Yb^{III} $^2F_{7/2} \rightarrow {}^2F_{5/2}$ transition.

Path. (p)	Nd^{III} transition	W_{d-d}	W_{d-q}	W_{q-q}	W_{ex}	W_{md-md}	$\omega_2^f(p)$	$\omega_2^b(p)$
1	$^4F_{3/2}\rightarrow{}^4I_{9/2}$	2.8×10^{-2}	6.4	0.0	4.0×10^{-22}	0.0	6.4	1.0×10^{-2}
2	$^4F_{5/2}\rightarrow{}^4I_{9/2}$	2.0×10^{-5}	4.5×10^{-3}	4.8×10^{-3}	1.6×10^{-25}	0.0	9.3×10^{-3}	1.5×10^{-7}
3	$^2H_{9/2}\rightarrow{}^4I_{9/2}$	9.0×10^{-7}	2.1×10^{-4}	1.5×10^{-2}	5.6×10^{-26}	2.6×10^{-6}	1.6×10^{-2}	2.5×10^{-7}
4	$^4F_{7/2}\rightarrow{}^4I_{9/2}$	1.4×10^{-10}	3.2×10^{-8}	7.7×10^{-8}	1.9×10^{-30}	4.4×10^{-13}	1.1×10^{-7}	2.4×10^{-14}
5	$^4S_{3/2}\rightarrow{}^4I_{9/2}$	5.3×10^{-11}	1.2×10^{-8}	0.0	6.9×10^{-31}	0.0	1.2×10^{-8}	9.3×10^{-16}
6	$^4F_{9/2}\rightarrow{}^4I_{9/2}$	6.1×10^{-21}	1.4×10^{-18}	2.6×10^{-17}	1.0×10^{-39}	1.0×10^{-20}	2.8×10^{-17}	1.6×10^{-26}
7	$^2H_{11/2}\rightarrow{}^4I_{9/2}$	3.6×10^{-33}	8.3×10^{-31}	0.0	2.8×10^{-51}	4.3×10^{-34}	8.3×10^{-31}	1.8×10^{-42}
8	$^4G_{5/2}\rightarrow{}^4I_{9/2}$	3.3×10^{-47}	1.8×10^{-44}	1.8×10^{-41}	4.1×10^{-67}	0.0	1.8×10^{-41}	3.3×10^{-56}
9	$^2G_{7/2}\rightarrow{}^4I_{9/2}$	2.9×10^{-48}	8.3×10^{-46}	3.0×10^{-43}	1.2×10^{-67}	0.0	3.0×10^{-43}	6.2×10^{-58}
10	$^4G_{7/2}\rightarrow{}^4I_{9/2}$	1.4×10^{-75}	3.9×10^{-73}	1.1×10^{-70}	5.0×10^{-95}	5.7×10^{-78}	1.1×10^{-70}	3.6×10^{-89}
11	$^4G_{9/2}\rightarrow{}^4I_{9/2}$	3.9×10^{-83}	9.1×10^{-81}	4.8×10^{-79}	3.8×10^{-102}	1.0×10^{-83}	4.9×10^{-79}	2.8×10^{-98}
12	$^2K_{13/2}\rightarrow{}^4I_{9/2}$	5.4×10^{-85}	1.4×10^{-82}	2.6×10^{-80}	1.7×10^{-103}	0.0	2.6×10^{-80}	1.4×10^{-99}
13	$^2D_{3/2}\rightarrow{}^4I_{9/2}$	2.7×10^{-116}	6.2×10^{-114}	0.0	5.9×10^{-135}	0.0	6.3×10^{-114}	3.7×10^{-137}
14	$^4G_{11/2}\rightarrow{}^4I_{9/2}$	6.3×10^{-123}	1.4×10^{-120}	0.0	5.0×10^{-141}	4.5×10^{-125}	1.4×10^{-120}	6.4×10^{-144}
15	$^2K_{15/2}\rightarrow{}^4I_{9/2}$	2.9×10^{-124}	6.7×10^{-122}	0.0	2.0×10^{-142}	0.0	6.7×10^{-122}	2.9×10^{-145}
16	$^2P_{1/2}\rightarrow{}^4I_{9/2}$	9.8×10^{-162}	2.2×10^{-159}	0.0	5.5×10^{-181}	0.0	2.2×10^{-159}	3.9×10^{-187}
17	$^4F_{3/2}\rightarrow{}^4I_{11/2}$	6.2×10^{-1}	1.4×10^{2}	0.0	3.8×10^{-21}	0.0	5.9	6.3×10^{1}
18	$^4F_{5/2}\rightarrow{}^4I_{11/2}$	2.4×10^{-1}	5.6×10^{1}	0.0	7.3×10^{-21}	0.0	5.6×10^{1}	6.2
19	$^2H_{9/2}\rightarrow{}^4I_{11/2}$	2.0×10^{-2}	4.8	4.8×10^{2}	5.9×10^{-21}	2.7×10^{-1}	4.9×10^{2}	5.4×10^{1}
20	$^4F_{7/2}\rightarrow{}^4I_{11/2}$	1.6×10^{-2}	3.6	6.8	2.1×10^{-22}	0.0	1.0×10^{1}	1.5×10^{-2}
21	$^4S_{3/2}\rightarrow{}^4I_{11/2}$	9.2×10^{-3}	2.1	0.0	1.4×10^{-22}	0.0	2.1	1.1×10^{-3}
22	$^4F_{9/2}\rightarrow{}^4I_{11/2}$	1.9×10^{-7}	4.2×10^{-5}	1.1×10^{-5}	3.7×10^{-27}	3.9×10^{-8}	5.3×10^{-5}	2.1×10^{-10}
23	$^2H_{11/2}\rightarrow{}^4I_{11/2}$	2.6×10^{-16}	7.0×10^{-14}	1.9×10^{-11}	1.8×10^{-34}	2.3×10^{-15}	1.9×10^{-11}	2.8×10^{-19}
24	$^4G_{5/2}\rightarrow{}^4I_{11/2}$	8.3×10^{-26}	1.9×10^{-23}	0.0	1.3×10^{-45}	0.0	1.9×10^{-23}	2.4×10^{-34}
25	$^2G_{7/2}\rightarrow{}^4I_{11/2}$	1.7×10^{-26}	8.2×10^{-24}	7.5×10^{-21}	5.2×10^{-46}	0.0	7.5×10^{-21}	1.1×10^{-31}
26	$^4G_{7/2}\rightarrow{}^4I_{11/2}$	1.3×10^{-47}	1.2×10^{-44}	1.5×10^{-41}	6.2×10^{-67}	0.0	1.5×10^{-41}	3.4×10^{-56}
27	$^4G_{9/2}\rightarrow{}^4I_{11/2}$	5.5×10^{-53}	1.6×10^{-50}	5.7×10^{-48}	1.4×10^{-72}	3.1×10^{-54}	5.7×10^{-48}	2.2×10^{-63}
28	$^2K_{13/2}\rightarrow{}^4I_{11/2}$	0.0	0.0	0.0	1.2×10^{-73}	1.9×10^{-54}	1.9×10^{-54}	7.2×10^{-70}
29	$^2D_{3/2}\rightarrow{}^4I_{11/2}$	2.8×10^{-80}	6.3×10^{-78}	0.0	2.5×10^{-99}	0.0	6.3×10^{-78}	2.6×10^{-97}
30	$^4G_{11/2}\rightarrow{}^4I_{11/2}$	0.0	0.0	0.0	2.3×10^{-104}	1.3×10^{-87}	1.3×10^{-87}	3.9×10^{-107}
31	$^2K_{15/2}\rightarrow{}^4I_{11/2}$	5.2×10^{-88}	1.5×10^{-85}	5.5×10^{-83}	1.5×10^{-105}	0.0	5.6×10^{-83}	1.7×10^{-102}
32	$^2P_{1/2}\rightarrow{}^4I_{11/2}$	0.0	0.0	0.0	3.6×10^{-138}	0.0	3.6×10^{-138}	1.9×10^{-161}
33	$^4F_{3/2}\rightarrow{}^4I_{13/2}$	1.7×10^{-7}	3.8×10^{-5}	0.0	2.5×10^{-27}	0.0	1.2×10^{-10}	1.5×10^{-5}
34	$^4F_{5/2}\rightarrow{}^4I_{13/2}$	4.1×10^{-3}	9.4×10^{-1}	0.0	3.6×10^{-23}	0.0	4.2×10^{-4}	5.4×10^{-1}
35	$^2H_{9/2}\rightarrow{}^4I_{13/2}$	1.2×10^{-3}	3.3×10^{-1}	8.5×10^{1}	7.5×10^{-23}	0.0	6.4×10^{-2}	8.1×10^{1}
36	$^4F_{7/2}\rightarrow{}^4I_{13/2}$	1.5×10^{-1}	3.4×10^{1}	0.0	3.9×10^{-21}	0.0	1.5	2.6×10^{1}
37	$^4S_{3/2}\rightarrow{}^4I_{13/2}$	5.1×10^{-1}	1.2×10^{2}	0.0	4.8×10^{-21}	0.0	7.3	4.4×10^{1}
38	$^4F_{9/2}\rightarrow{}^4I_{13/2}$	3.6×10^{-1}	8.1×10^{1}	3.5×10^{2}	4.2×10^{-21}	0.0	4.3×10^{2}	2.0×10^{1}
39	$^2H_{11/2}\rightarrow{}^4I_{13/2}$	1.1×10^{-5}	2.8×10^{-3}	6.9×10^{-1}	6.6×10^{-24}	2.9×10^{-4}	7.0×10^{-1}	1.2×10^{-4}
40	$^4G_{5/2}\rightarrow{}^4I_{13/2}$	6.3×10^{-11}	1.4×10^{-8}	0.0	4.0×10^{-30}	0.0	1.4×10^{-8}	2.2×10^{-15}
41	$^2G_{7/2}\rightarrow{}^4I_{13/2}$	3.5×10^{-11}	8.1×10^{-9}	0.0	2.3×10^{-30}	0.0	8.1×10^{-9}	1.3×10^{-15}
42	$^4G_{7/2}\rightarrow{}^4I_{13/2}$	6.6×10^{-25}	1.5×10^{-22}	0.0	1.8×10^{-44}	0.0	1.5×10^{-22}	4.0×10^{-33}
43	$^4G_{9/2}\rightarrow{}^4I_{13/2}$	6.3×10^{-29}	3.8×10^{-26}	4.0×10^{-23}	1.4×10^{-48}	0.0	4.0×10^{-23}	1.8×10^{-34}
44	$^2K_{13/2}\rightarrow{}^4I_{13/2}$	5.8×10^{-32}	2.2×10^{-29}	1.6×10^{-26}	2.3×10^{-49}	3.1×10^{-30}	1.6×10^{-26}	6.8×10^{-38}
45	$^2D_{3/2}\rightarrow{}^4I_{13/2}$	7.2×10^{-50}	1.6×10^{-47}	0.0	6.6×10^{-69}	0.0	1.6×10^{-47}	7.8×10^{-63}
46	$^4G_{11/2}\rightarrow{}^4I_{13/2}$	3.2×10^{-53}	8.5×10^{-51}	2.2×10^{-48}	7.1×10^{-73}	9.1×10^{-55}	2.2×10^{-48}	7.9×10^{-64}

47	$^2K_{15/2}\rightarrow{}^4I_{13/2}$	1.2×10^{-55}	2.7×10^{-53}	4.6×10^{-52}	8.4×10^{-74}	1.3×10^{-54}	4.9×10^{-52}	1.7×10^{-67}
48	$^2P_{1/2}\rightarrow{}^4I_{13/2}$	0.0	0.0	0.0	3.6×10^{-100}	0.0	3.6×10^{-100}	2.6×10^{-119}
49	$^4F_{3/2}\rightarrow{}^4I_{15/2}$	1.5×10^{-22}	3.5×10^{-20}	0.0	1.7×10^{-41}	0.0	5.8×10^{-30}	1.2×10^{-20}
50	$^4F_{5/2}\rightarrow{}^4I_{15/2}$	1.2×10^{-13}	2.8×10^{-11}	0.0	2.5×10^{-33}	0.0	6.8×10^{-19}	1.4×10^{-11}
51	$^2H_{9/2}\rightarrow{}^4I_{15/2}$	4.0×10^{-13}	9.1×10^{-11}	0.0	1.4×10^{-32}	0.0	3.7×10^{-18}	7.6×10^{-11}
52	$^4F_{7/2}\rightarrow{}^4I_{15/2}$	1.6×10^{-7}	3.6×10^{-5}	0.0	1.4×10^{-27}	0.0	8.7×10^{-11}	2.4×10^{-5}
53	$^4S_{3/2}\rightarrow{}^4I_{15/2}$	3.3×10^{-7}	7.6×10^{-5}	0.0	3.1×10^{-27}	0.0	2.6×10^{-10}	2.5×10^{-5}
54	$^4F_{9/2}\rightarrow{}^4I_{15/2}$	1.3×10^{-2}	3.0	0.0	1.2×10^{-22}	0.0	3.3×10^{-3}	2.5
55	$^2H_{11/2}\rightarrow{}^4I_{15/2}$	5.4×10^{-2}	2.9×10^{1}	2.8×10^{4}	8.9×10^{-21}	0.0	2.8×10^{4}	1.0×10^{4}
56	$^4G_{5/2}\rightarrow{}^4I_{15/2}$	6.8×10^{-4}	1.5×10^{-1}	0.0	6.9×10^{-22}	0.0	1.6×10^{-1}	3.8×10^{-4}
57	$^2G_{7/2}\rightarrow{}^4I_{15/2}$	9.7×10^{-3}	2.2	0.0	5.6×10^{-22}	0.0	2.2	5.9×10^{-3}
58	$^4G_{7/2}\rightarrow{}^4I_{15/2}$	1.8×10^{-10}	4.1×10^{-8}	0.0	4.7×10^{-29}	0.0	4.1×10^{-8}	1.8×10^{-14}
59	$^4G_{9/2}\rightarrow{}^4I_{15/2}$	6.7×10^{-12}	1.5×10^{-9}	0.0	1.5×10^{-31}	0.0	1.5×10^{-9}	1.1×10^{-16}
60	$^2K_{13/2}\rightarrow{}^4I_{15/2}$	3.2×10^{-14}	7.3×10^{-12}	0.0	4.9×10^{-32}	6.1×10^{-15}	7.3×10^{-12}	5.1×10^{-19}
61	$^2D_{3/2}\rightarrow{}^4I_{15/2}$	7.8×10^{-27}	1.8×10^{-24}	0.0	2.9×10^{-45}	0.0	1.8×10^{-24}	1.4×10^{-35}
62	$^4G_{11/2}\rightarrow{}^4I_{15/2}$	3.9×10^{-28}	1.8×10^{-25}	1.5×10^{-22}	4.1×10^{-48}	0.0	1.5×10^{-22}	8.8×10^{-34}
63	$^2K_{15/2}\rightarrow{}^4I_{15/2}$	1.7×10^{-30}	4.8×10^{-28}	1.7×10^{-25}	8.7×10^{-49}	2.9×10^{-29}	1.7×10^{-25}	9.4×10^{-37}
64	$^2P_{1/2}\rightarrow{}^4I_{15/2}$	0.0	0.0	0.0	1.1×10^{-68}	0.0	1.1×10^{-68}	1.5×10^{-83}
					$W_2^f = \sum_{p=1}^{64} \omega_2^f(p) =$		2.9×10^{4} s^{-1}	
					$W_2^b = \sum_{p=1}^{64} \omega_2^b(p) =$		1.0×10^{4} s^{-1}	

Table S10. Pairwise nonradiative energy transfer rates (s^{-1}) from Nd^{III} to Yb^{III} at the distance order 3 (R= 7.26 Å) at 300 K. All pathways involve the Yb^{III} $^2F_{7/2} \rightarrow {}^2F_{5/2}$ transition.

Path. (p)	Nd^{III} transition	$\mathbf{W_{d-d}}$	$\mathbf{W_{d-q}}$	$\mathbf{W_{q-q}}$	$\mathbf{W_{ex}}$	$\mathbf{W_{md-md}}$	$\omega_3^f(p)$	$\omega_3^b(p)$
1	$^4F_{3/2}\rightarrow{}^4I_{9/2}$	2.3×10^{-2}	5.0	0.0	2.9×10^{-24}	0.0	5.0	7.8×10^{-3}
2	$^4F_{5/2}\rightarrow{}^4I_{9/2}$	1.6×10^{-5}	3.5×10^{-3}	3.5×10^{-3}	1.2×10^{-27}	0.0	7.0×10^{-3}	1.1×10^{-7}
3	$^2H_{9/2}\rightarrow{}^4I_{9/2}$	7.4×10^{-7}	1.7×10^{-4}	1.1×10^{-2}	4.0×10^{-28}	2.1×10^{-6}	1.1×10^{-2}	1.8×10^{-7}
4	$^4F_{7/2}\rightarrow{}^4I_{9/2}$	1.2×10^{-10}	2.5×10^{-8}	5.6×10^{-8}	1.4×10^{-32}	3.6×10^{-13}	8.1×10^{-8}	1.8×10^{-14}
5	$^4S_{3/2}\rightarrow{}^4I_{9/2}$	4.4×10^{-11}	9.4×10^{-9}	0.0	5.0×10^{-33}	0.0	9.4×10^{-9}	7.2×10^{-16}
6	$^4F_{9/2}\rightarrow{}^4I_{9/2}$	5.1×10^{-21}	1.1×10^{-18}	1.9×10^{-17}	7.2×10^{-42}	8.3×10^{-21}	2.0×10^{-17}	1.2×10^{-26}
7	$^2H_{11/2}\rightarrow{}^4I_{9/2}$	3.0×10^{-33}	6.4×10^{-31}	0.0	2.0×10^{-53}	3.6×10^{-34}	6.5×10^{-31}	1.4×10^{-42}
8	$^4G_{5/2}\rightarrow{}^4I_{9/2}$	2.7×10^{-47}	1.4×10^{-44}	1.3×10^{-41}	2.9×10^{-69}	0.0	1.3×10^{-41}	2.4×10^{-56}
9	$^2G_{7/2}\rightarrow{}^4I_{9/2}$	2.4×10^{-48}	6.4×10^{-46}	2.2×10^{-43}	8.3×10^{-70}	0.0	2.2×10^{-43}	4.5×10^{-58}
10	$^4G_{7/2}\rightarrow{}^4I_{9/2}$	1.2×10^{-75}	3.1×10^{-73}	7.9×10^{-71}	3.5×10^{-97}	4.7×10^{-78}	7.9×10^{-71}	2.6×10^{-89}
11	$^4G_{9/2}\rightarrow{}^4I_{9/2}$	3.2×10^{-83}	7.1×10^{-81}	3.5×10^{-79}	2.7×10^{-104}	8.3×10^{-84}	3.6×10^{-79}	2.0×10^{-98}
12	$^2K_{13/2}\rightarrow{}^4I_{9/2}$	4.5×10^{-85}	1.1×10^{-82}	1.9×10^{-80}	1.2×10^{-105}	0.0	1.9×10^{-80}	1.0×10^{-99}
13	$^2D_{3/2}\rightarrow{}^4I_{9/2}$	2.3×10^{-116}	4.8×10^{-114}	0.0	4.2×10^{-137}	0.0	4.9×10^{-114}	2.9×10^{-137}
14	$^4G_{11/2}\rightarrow{}^4I_{9/2}$	5.2×10^{-123}	1.1×10^{-120}	0.0	3.6×10^{-143}	3.7×10^{-125}	1.1×10^{-120}	5.0×10^{-144}
15	$^2K_{15/2}\rightarrow{}^4I_{9/2}$	2.4×10^{-124}	5.2×10^{-122}	0.0	1.4×10^{-144}	0.0	5.2×10^{-122}	2.3×10^{-145}
16	$^2P_{1/2}\rightarrow{}^4I_{9/2}$	8.1×10^{-162}	1.7×10^{-159}	0.0	3.9×10^{-183}	0.0	1.7×10^{-159}	3.0×10^{-187}
17	$^4F_{3/2}\rightarrow{}^4I_{11/2}$	5.1×10^{-1}	1.1×10^{2}	0.0	2.7×10^{-23}	0.0	4.6	4.9×10^{1}
18	$^4F_{5/2}\rightarrow{}^4I_{11/2}$	2.0×10^{-1}	4.3×10^{1}	0.0	5.2×10^{-23}	0.0	4.3×10^{1}	4.8
19	$^2H_{9/2}\rightarrow{}^4I_{11/2}$	1.6×10^{-2}	3.7	3.5×10^{2}	4.2×10^{-23}	2.3×10^{-1}	3.6×10^{2}	3.9×10^{1}
20	$^4F_{7/2}\rightarrow{}^4I_{11/2}$	1.3×10^{-2}	2.8	5.0	1.5×10^{-24}	0.0	7.8	1.2×10^{-2}
21	$^4S_{3/2}\rightarrow{}^4I_{11/2}$	7.6×10^{-3}	1.6	0.0	9.7×10^{-25}	0.0	1.6	8.6×10^{-4}
22	$^4F_{9/2}\rightarrow{}^4I_{11/2}$	1.5×10^{-7}	3.3×10^{-5}	7.8×10^{-6}	2.6×10^{-29}	3.2×10^{-8}	4.1×10^{-5}	1.6×10^{-10}
23	$^2H_{11/2}\rightarrow{}^4I_{11/2}$	2.1×10^{-16}	5.5×10^{-14}	1.4×10^{-11}	1.3×10^{-36}	1.9×10^{-15}	1.4×10^{-11}	2.1×10^{-19}
24	$^4G_{5/2}\rightarrow{}^4I_{11/2}$	6.8×10^{-26}	1.5×10^{-23}	0.0	9.2×10^{-48}	0.0	1.5×10^{-23}	1.9×10^{-34}
25	$^2G_{7/2}\rightarrow{}^4I_{11/2}$	1.4×10^{-26}	6.4×10^{-24}	5.5×10^{-21}	3.7×10^{-48}	0.0	5.5×10^{-21}	7.8×10^{-32}
26	$^4G_{7/2}\rightarrow{}^4I_{11/2}$	1.0×10^{-47}	9.1×10^{-45}	1.1×10^{-41}	4.4×10^{-69}	0.0	1.1×10^{-41}	2.5×10^{-56}
27	$^4G_{9/2}\rightarrow{}^4I_{11/2}$	4.5×10^{-53}	1.2×10^{-50}	4.2×10^{-48}	1.0×10^{-74}	2.6×10^{-54}	4.2×10^{-48}	1.6×10^{-63}
28	$^2K_{13/2}\rightarrow{}^4I_{11/2}$	0.0	0.0	0.0	8.5×10^{-76}	1.6×10^{-54}	1.6×10^{-54}	6.0×10^{-70}
29	$^2D_{3/2}\rightarrow{}^4I_{11/2}$	2.3×10^{-80}	4.9×10^{-78}	0.0	1.8×10^{-101}	0.0	4.9×10^{-78}	2.0×10^{-97}
30	$^4G_{11/2}\rightarrow{}^4I_{11/2}$	0.0	0.0	0.0	1.6×10^{-106}	1.1×10^{-87}	1.1×10^{-87}	3.2×10^{-107}
31	$^2K_{15/2}\rightarrow{}^4I_{11/2}$	4.3×10^{-88}	1.2×10^{-85}	4.0×10^{-83}	1.1×10^{-107}	0.0	4.1×10^{-83}	1.2×10^{-102}
32	$^2P_{1/2}\rightarrow{}^4I_{11/2}$	0.0	0.0	0.0	2.6×10^{-140}	0.0	2.6×10^{-140}	1.4×10^{-163}
33	$^4F_{3/2}\rightarrow{}^4I_{13/2}$	1.4×10^{-7}	3.0×10^{-5}	0.0	1.8×10^{-29}	0.0	9.1×10^{-11}	1.1×10^{-5}
34	$^4F_{5/2}\rightarrow{}^4I_{13/2}$	3.4×10^{-3}	7.3×10^{-1}	0.0	2.6×10^{-25}	0.0	3.2×10^{-4}	4.2×10^{-1}
35	$^2H_{9/2}\rightarrow{}^4I_{13/2}$	1.0×10^{-3}	2.6×10^{-1}	6.2×10^{1}	5.3×10^{-25}	0.0	4.6×10^{-2}	5.9×10^{1}
36	$^4F_{7/2}\rightarrow{}^4I_{13/2}$	1.2×10^{-1}	2.6×10^{1}	0.0	2.8×10^{-23}	0.0	1.2	2.0×10^{1}
37	$^4S_{3/2}\rightarrow{}^4I_{13/2}$	4.2×10^{-1}	9.0×10^{1}	0.0	3.4×10^{-23}	0.0	5.6	3.4×10^{1}
38	$^4F_{9/2}\rightarrow{}^4I_{13/2}$	2.9×10^{-1}	6.3×10^{1}	2.6×10^{2}	3.0×10^{-23}	0.0	3.2×10^{2}	1.5×10^{1}
39	$^2H_{11/2}\rightarrow{}^4I_{13/2}$	8.8×10^{-6}	2.2×10^{-3}	5.1×10^{-1}	4.7×10^{-26}	2.4×10^{-4}	5.1×10^{-1}	8.7×10^{-5}
40	$^4G_{5/2}\rightarrow{}^4I_{13/2}$	5.2×10^{-11}	1.1×10^{-8}	0.0	2.9×10^{-32}	0.0	1.1×10^{-8}	1.7×10^{-15}
41	$^2G_{7/2}\rightarrow{}^4I_{13/2}$	2.9×10^{-11}	6.3×10^{-9}	0.0	1.6×10^{-32}	0.0	6.3×10^{-9}	1.0×10^{-15}
42	$^4G_{7/2}\rightarrow{}^4I_{13/2}$	5.5×10^{-25}	1.2×10^{-22}	0.0	1.3×10^{-46}	0.0	1.2×10^{-22}	3.1×10^{-33}
43	$^4G_{9/2}\rightarrow{}^4I_{13/2}$	5.2×10^{-29}	2.9×10^{-26}	2.9×10^{-23}	1.0×10^{-50}	0.0	2.9×10^{-23}	1.3×10^{-34}
44	$^2K_{13/2}\rightarrow{}^4I_{13/2}$	4.8×10^{-32}	1.7×10^{-29}	1.1×10^{-26}	1.7×10^{-51}	2.5×10^{-30}	1.1×10^{-26}	4.9×10^{-38}
45	$^2D_{3/2}\rightarrow{}^4I_{13/2}$	5.9×10^{-50}	1.3×10^{-47}	0.0	4.7×10^{-71}	0.0	1.3×10^{-47}	6.1×10^{-63}
46	$^4G_{11/2}\rightarrow{}^4I_{13/2}$	2.6×10^{-53}	6.6×10^{-51}	1.6×10^{-48}	5.1×10^{-75}	7.5×10^{-55}	1.6×10^{-48}	5.8×10^{-64}

47	$^2K_{15/2}\rightarrow{}^4I_{13/2}$	9.7×10^{-56}	2.1×10^{-53}	3.3×10^{-52}	6.0×10^{-76}	1.0×10^{-54}	3.6×10^{-52}	1.2×10^{-67}
48	$^2P_{1/2}\rightarrow{}^4I_{13/2}$	0.0	0.0	0.0	2.6×10^{-102}	0.0	2.6×10^{-102}	1.9×10^{-121}
49	$^4F_{3/2}\rightarrow{}^4I_{15/2}$	1.3×10^{-22}	2.7×10^{-20}	0.0	1.2×10^{-43}	0.0	4.5×10^{-30}	9.1×10^{-21}
50	$^4F_{5/2}\rightarrow{}^4I_{15/2}$	1.0×10^{-13}	2.2×10^{-11}	0.0	1.8×10^{-35}	0.0	5.3×10^{-19}	1.1×10^{-11}
51	$^2H_{9/2}\rightarrow{}^4I_{15/2}$	3.3×10^{-13}	7.1×10^{-11}	0.0	9.7×10^{-35}	0.0	2.9×10^{-18}	5.9×10^{-11}
52	$^4F_{7/2}\rightarrow{}^4I_{15/2}$	1.3×10^{-7}	2.8×10^{-5}	0.0	9.7×10^{-30}	0.0	6.8×10^{-11}	1.9×10^{-5}
53	$^4S_{3/2}\rightarrow{}^4I_{15/2}$	2.8×10^{-7}	5.9×10^{-5}	0.0	2.2×10^{-29}	0.0	2.0×10^{-10}	2.0×10^{-5}
54	$^4F_{9/2}\rightarrow{}^4I_{15/2}$	1.1×10^{-2}	2.3	0.0	8.9×10^{-25}	0.0	2.6×10^{-3}	1.9
55	$^2H_{11/2}\rightarrow{}^4I_{15/2}$	4.5×10^{-2}	2.2×10^{1}	2.0×10^{4}	6.4×10^{-23}	0.0	2.0×10^{4}	7.3×10^{3}
56	$^4G_{5/2}\rightarrow{}^4I_{15/2}$	5.6×10^{-4}	1.2×10^{-1}	0.0	4.9×10^{-24}	0.0	1.2×10^{-1}	2.9×10^{-4}
57	$^2G_{7/2}\rightarrow{}^4I_{15/2}$	8.0×10^{-3}	1.7	0.0	4.0×10^{-24}	0.0	1.7	4.6×10^{-3}
58	$^4G_{7/2}\rightarrow{}^4I_{15/2}$	1.5×10^{-10}	3.2×10^{-8}	0.0	3.4×10^{-31}	0.0	3.2×10^{-8}	1.4×10^{-14}
59	$^4G_{9/2}\rightarrow{}^4I_{15/2}$	5.5×10^{-12}	1.2×10^{-9}	0.0	1.1×10^{-33}	0.0	1.2×10^{-9}	8.6×10^{-17}
60	$^2K_{13/2}\rightarrow{}^4I_{15/2}$	2.6×10^{-14}	5.6×10^{-12}	0.0	3.5×10^{-34}	5.1×10^{-15}	5.7×10^{-12}	4.0×10^{-19}
61	$^2D_{3/2}\rightarrow{}^4I_{15/2}$	6.5×10^{-27}	1.4×10^{-24}	0.0	2.1×10^{-47}	0.0	1.4×10^{-24}	1.1×10^{-35}
62	$^4G_{11/2}\rightarrow{}^4I_{15/2}$	3.2×10^{-28}	1.4×10^{-25}	1.1×10^{-22}	2.9×10^{-50}	0.0	1.1×10^{-22}	6.4×10^{-34}
63	$^2K_{15/2}\rightarrow{}^4I_{15/2}$	1.4×10^{-30}	3.7×10^{-28}	1.2×10^{-25}	6.2×10^{-51}	2.4×10^{-29}	1.2×10^{-25}	6.8×10^{-37}
64	$^2P_{1/2}\rightarrow{}^4I_{15/2}$	0.0	0.0	0.0	8.0×10^{-71}	0.0	8.0×10^{-71}	1.1×10^{-85}
					$W_3^f = \sum_{p=1}^{64} \omega_3^f(p) =$		2.1×10^4 s^{-1}	
					$W_3^b = \sum_{p=1}^{64} \omega_3^b(p) =$		7.5×10^3 s^{-1}	

Table S11. Pairwise nonradiative energy transfer rates (s^{-1}) from Nd^{III} to Yb^{III} at the distance order 4 (R= 8.66 Å) at 300 K. All pathways involve the Yb^{III} $^2F_{7/2} \rightarrow {}^2F_{5/2}$ transition.

Path. (p)	Nd^{III} transition	W_{d-d}	W_{d-q}	W_{q-q}	W_{ex}	W_{md-md}	$\omega_4^f(p)$	$\omega_4^b(p)$
1	$^4F_{3/2}\rightarrow{}^4I_{9/2}$	8.0×10^{-3}	1.2	0.0	6.6×10^{-39}	0.0	1.2	1.9×10^{-3}
2	$^4F_{5/2}\rightarrow{}^4I_{9/2}$	5.7×10^{-6}	8.5×10^{-4}	5.9×10^{-4}	2.7×10^{-42}	0.0	1.5×10^{-3}	2.3×10^{-8}
3	$^2H_{9/2}\rightarrow{}^4I_{9/2}$	2.6×10^{-7}	4.1×10^{-5}	1.9×10^{-3}	9.1×10^{-43}	7.4×10^{-7}	2.0×10^{-3}	3.1×10^{-8}
4	$^4F_{7/2}\rightarrow{}^4I_{9/2}$	4.0×10^{-11}	6.1×10^{-9}	9.6×10^{-9}	3.1×10^{-47}	1.3×10^{-13}	1.6×10^{-8}	3.4×10^{-15}
5	$^4S_{3/2}\rightarrow{}^4I_{9/2}$	1.5×10^{-11}	2.3×10^{-9}	0.0	1.1×10^{-47}	0.0	2.3×10^{-9}	1.8×10^{-16}
6	$^4F_{9/2}\rightarrow{}^4I_{9/2}$	1.8×10^{-21}	2.7×10^{-19}	3.3×10^{-18}	1.7×10^{-56}	2.9×10^{-21}	3.6×10^{-18}	2.1×10^{-27}
7	$^2H_{11/2}\rightarrow{}^4I_{9/2}$	1.0×10^{-33}	1.6×10^{-31}	0.0	4.5×10^{-68}	1.2×10^{-34}	1.6×10^{-31}	3.4×10^{-43}
8	$^4G_{5/2}\rightarrow{}^4I_{9/2}$	9.5×10^{-48}	3.4×10^{-45}	2.2×10^{-42}	6.7×10^{-84}	0.0	2.2×10^{-42}	4.2×10^{-57}
9	$^2G_{7/2}\rightarrow{}^4I_{9/2}$	8.2×10^{-49}	1.6×10^{-46}	3.7×10^{-44}	1.9×10^{-84}	0.0	3.7×10^{-44}	7.8×10^{-59}
10	$^4G_{7/2}\rightarrow{}^4I_{9/2}$	4.1×10^{-76}	7.4×10^{-74}	1.3×10^{-71}	8.2×10^{-112}	1.6×10^{-78}	1.4×10^{-71}	4.5×10^{-90}
11	$^4G_{9/2}\rightarrow{}^4I_{9/2}$	1.1×10^{-83}	1.7×10^{-81}	6.1×10^{-80}	6.2×10^{-119}	2.9×10^{-84}	6.2×10^{-80}	3.5×10^{-99}
12	$^2K_{13/2}\rightarrow{}^4I_{9/2}$	1.5×10^{-85}	2.6×10^{-83}	3.2×10^{-81}	2.9×10^{-120}	0.0	3.2×10^{-81}	1.8×10^{-100}
13	$^2D_{3/2}\rightarrow{}^4I_{9/2}$	7.8×10^{-117}	1.2×10^{-114}	0.0	9.7×10^{-152}	0.0	1.2×10^{-114}	7.1×10^{-138}
14	$^4G_{11/2}\rightarrow{}^4I_{9/2}$	1.8×10^{-123}	2.7×10^{-121}	0.0	8.3×10^{-158}	1.3×10^{-125}	2.7×10^{-121}	1.2×10^{-144}
15	$^2K_{15/2}\rightarrow{}^4I_{9/2}$	8.4×10^{-125}	1.3×10^{-122}	0.0	3.2×10^{-159}	0.0	1.3×10^{-122}	5.6×10^{-146}
16	$^2P_{1/2}\rightarrow{}^4I_{9/2}$	2.8×10^{-162}	4.2×10^{-160}	0.0	9.0×10^{-198}	0.0	4.3×10^{-160}	7.4×10^{-188}
17	$^4F_{3/2}\rightarrow{}^4I_{11/2}$	1.8×10^{-1}	2.7×10^{1}	0.0	6.2×10^{-38}	0.0	1.1	1.2×10^{1}
18	$^4F_{5/2}\rightarrow{}^4I_{11/2}$	7.0×10^{-2}	1.1×10^{1}	0.0	1.2×10^{-37}	0.0	1.1×10^{1}	1.2
19	$^2H_{9/2}\rightarrow{}^4I_{11/2}$	5.7×10^{-3}	9.1×10^{-1}	6.0×10^{1}	9.8×10^{-38}	7.8×10^{-2}	6.1×10^{1}	6.7
20	$^4F_{7/2}\rightarrow{}^4I_{11/2}$	4.5×10^{-3}	6.8×10^{-1}	8.5×10^{-1}	3.4×10^{-39}	0.0	1.5	2.3×10^{-3}
21	$^4S_{3/2}\rightarrow{}^4I_{11/2}$	2.6×10^{-3}	4.0×10^{-1}	0.0	2.2×10^{-39}	0.0	4.0×10^{-1}	2.1×10^{-4}
22	$^4F_{9/2}\rightarrow{}^4I_{11/2}$	5.3×10^{-8}	8.0×10^{-6}	1.3×10^{-6}	6.0×10^{-44}	1.1×10^{-8}	9.4×10^{-6}	3.8×10^{-11}
23	$^2H_{11/2}\rightarrow{}^4I_{11/2}$	7.4×10^{-17}	1.3×10^{-14}	2.4×10^{-12}	3.0×10^{-51}	6.6×10^{-16}	2.4×10^{-12}	3.6×10^{-20}
24	$^4G_{5/2}\rightarrow{}^4I_{11/2}$	2.4×10^{-26}	3.6×10^{-24}	0.0	2.1×10^{-62}	0.0	3.6×10^{-24}	4.6×10^{-35}
25	$^2G_{7/2}\rightarrow{}^4I_{11/2}$	4.8×10^{-27}	1.6×10^{-24}	9.4×10^{-22}	8.5×10^{-63}	0.0	9.4×10^{-22}	1.3×10^{-32}
26	$^4G_{7/2}\rightarrow{}^4I_{11/2}$	3.6×10^{-48}	2.2×10^{-45}	1.9×10^{-42}	1.0×10^{-83}	0.0	1.9×10^{-42}	4.2×10^{-57}
27	$^4G_{9/2}\rightarrow{}^4I_{11/2}$	1.6×10^{-53}	3.0×10^{-51}	7.1×10^{-49}	2.3×10^{-89}	9.0×10^{-55}	7.1×10^{-49}	2.7×10^{-64}
28	$^2K_{13/2}\rightarrow{}^4I_{11/2}$	0.0	0.0	0.0	2.0×10^{-90}	5.6×10^{-55}	5.6×10^{-55}	2.1×10^{-70}
29	$^2D_{3/2}\rightarrow{}^4I_{11/2}$	7.9×10^{-81}	1.2×10^{-78}	0.0	4.2×10^{-116}	0.0	1.2×10^{-78}	4.9×10^{-98}
30	$^4G_{11/2}\rightarrow{}^4I_{11/2}$	0.0	0.0	0.0	3.7×10^{-121}	3.7×10^{-88}	3.7×10^{-88}	1.1×10^{-107}
31	$^2K_{15/2}\rightarrow{}^4I_{11/2}$	1.5×10^{-88}	2.9×10^{-86}	6.9×10^{-84}	2.5×10^{-122}	0.0	6.9×10^{-84}	2.1×10^{-103}
32	$^2P_{1/2}\rightarrow{}^4I_{11/2}$	0.0	0.0	0.0	5.9×10^{-155}	0.0	5.9×10^{-155}	3.2×10^{-178}
33	$^4F_{3/2}\rightarrow{}^4I_{13/2}$	4.8×10^{-8}	7.2×10^{-6}	0.0	4.1×10^{-44}	0.0	2.2×10^{-11}	2.8×10^{-6}
34	$^4F_{5/2}\rightarrow{}^4I_{13/2}$	1.2×10^{-3}	1.8×10^{-1}	0.0	5.9×10^{-40}	0.0	7.9×10^{-5}	1.0×10^{-1}
35	$^2H_{9/2}\rightarrow{}^4I_{13/2}$	3.6×10^{-4}	6.3×10^{-2}	1.1×10^{1}	1.2×10^{-39}	0.0	7.9×10^{-3}	1.0×10^{1}
36	$^4F_{7/2}\rightarrow{}^4I_{13/2}$	4.3×10^{-2}	6.4	0.0	6.5×10^{-38}	0.0	2.9×10^{-1}	4.9
37	$^4S_{3/2}\rightarrow{}^4I_{13/2}$	1.5×10^{-1}	2.2×10^{1}	0.0	7.9×10^{-38}	0.0	1.4	8.4
38	$^4F_{9/2}\rightarrow{}^4I_{13/2}$	1.0×10^{-1}	1.5×10^{1}	4.4×10^{1}	6.9×10^{-38}	0.0	5.9×10^{1}	2.8
39	$^2H_{11/2}\rightarrow{}^4I_{13/2}$	3.1×10^{-6}	5.4×10^{-4}	8.6×10^{-2}	1.1×10^{-40}	8.4×10^{-5}	8.7×10^{-2}	1.5×10^{-5}
40	$^4G_{5/2}\rightarrow{}^4I_{13/2}$	1.8×10^{-11}	2.7×10^{-9}	0.0	6.6×10^{-47}	0.0	2.7×10^{-9}	4.1×10^{-16}
41	$^2G_{7/2}\rightarrow{}^4I_{13/2}$	1.0×10^{-11}	1.5×10^{-9}	0.0	3.8×10^{-47}	0.0	1.5×10^{-9}	2.5×10^{-16}
42	$^4G_{7/2}\rightarrow{}^4I_{13/2}$	1.9×10^{-25}	2.8×10^{-23}	0.0	2.9×10^{-61}	0.0	2.9×10^{-23}	7.5×10^{-34}
43	$^4G_{9/2}\rightarrow{}^4I_{13/2}$	1.8×10^{-29}	7.1×10^{-27}	4.9×10^{-24}	2.3×10^{-65}	0.0	5.0×10^{-24}	2.2×10^{-35}
44	$^2K_{13/2}\rightarrow{}^4I_{13/2}$	1.7×10^{-32}	4.2×10^{-30}	2.0×10^{-27}	3.9×10^{-66}	8.8×10^{-31}	2.0×10^{-27}	8.5×10^{-39}
45	$^2D_{3/2}\rightarrow{}^4I_{13/2}$	2.1×10^{-50}	3.1×10^{-48}	0.0	1.1×10^{-85}	0.0	3.1×10^{-48}	1.5×10^{-63}

46	$^4G_{11/2}\to{}^4I_{13/2}$	9.1×10^{-54}	1.6×10^{-51}	2.8×10^{-49}	1.2×10^{-89}	2.6×10^{-55}	2.8×10^{-49}	9.9×10^{-65}
47	$^2K_{15/2}\to{}^4I_{13/2}$	3.4×10^{-56}	5.1×10^{-54}	5.7×10^{-53}	1.4×10^{-90}	3.6×10^{-55}	6.3×10^{-53}	2.2×10^{-68}
48	$^2P_{1/2}\to{}^4I_{13/2}$	0.0	0.0	0.0	6.0×10^{-117}	0.0	6.0×10^{-117}	4.3×10^{-136}
49	$^4F_{3/2}\to{}^4I_{15/2}$	4.4×10^{-23}	6.6×10^{-21}	0.0	2.8×10^{-58}	0.0	1.1×10^{-30}	2.2×10^{-21}
50	$^4F_{5/2}\to{}^4I_{15/2}$	3.5×10^{-14}	5.3×10^{-12}	0.0	4.1×10^{-50}	0.0	1.3×10^{-19}	2.7×10^{-12}
51	$^2H_{9/2}\to{}^4I_{15/2}$	1.1×10^{-13}	1.7×10^{-11}	0.0	2.2×10^{-49}	0.0	7.0×10^{-19}	1.4×10^{-11}
52	$^4F_{7/2}\to{}^4I_{15/2}$	4.6×10^{-8}	6.9×10^{-6}	0.0	2.2×10^{-44}	0.0	1.6×10^{-11}	4.6×10^{-6}
53	$^4S_{3/2}\to{}^4I_{15/2}$	9.6×10^{-8}	1.4×10^{-5}	0.0	5.2×10^{-44}	0.0	4.9×10^{-11}	4.8×10^{-6}
54	$^4F_{9/2}\to{}^4I_{15/2}$	3.8×10^{-3}	5.7×10^{-1}	0.0	2.1×10^{-39}	0.0	6.3×10^{-4}	4.7×10^{-1}
55	$^2H_{11/2}\to{}^4I_{15/2}$	1.5×10^{-2}	5.4	3.5×10^{3}	1.5×10^{-37}	0.0	3.5×10^{3}	1.3×10^{3}
56	$^4G_{5/2}\to{}^4I_{15/2}$	1.9×10^{-4}	2.9×10^{-2}	0.0	1.1×10^{-38}	0.0	2.9×10^{-2}	7.2×10^{-5}
57	$^2G_{7/2}\to{}^4I_{15/2}$	2.8×10^{-3}	4.2×10^{-1}	0.0	9.2×10^{-39}	0.0	4.2×10^{-1}	1.1×10^{-3}
58	$^4G_{7/2}\to{}^4I_{15/2}$	5.2×10^{-11}	7.8×10^{-9}	0.0	7.7×10^{-46}	0.0	7.8×10^{-9}	3.3×10^{-15}
59	$^4G_{9/2}\to{}^4I_{15/2}$	1.9×10^{-12}	2.9×10^{-10}	0.0	2.5×10^{-48}	0.0	2.9×10^{-10}	2.1×10^{-17}
60	$^2K_{13/2}\to{}^4I_{15/2}$	9.1×10^{-15}	1.4×10^{-12}	0.0	8.1×10^{-49}	1.8×10^{-15}	1.4×10^{-12}	9.8×10^{-20}
61	$^2D_{3/2}\to{}^4I_{15/2}$	2.2×10^{-27}	3.4×10^{-25}	0.0	4.8×10^{-62}	0.0	3.4×10^{-25}	2.6×10^{-36}
62	$^4G_{11/2}\to{}^4I_{15/2}$	1.1×10^{-28}	3.4×10^{-26}	1.9×10^{-23}	6.7×10^{-65}	0.0	1.9×10^{-23}	1.1×10^{-34}
63	$^2K_{15/2}\to{}^4I_{15/2}$	4.8×10^{-31}	9.0×10^{-29}	2.1×10^{-26}	1.4×10^{-65}	8.3×10^{-30}	2.1×10^{-26}	1.2×10^{-37}
64	$^2P_{1/2}\to{}^4I_{15/2}$	0.0	0.0	0.0	1.8×10^{-85}	0.0	1.8×10^{-85}	2.5×10^{-100}

$$W_4^f = \sum_{p=1}^{64} \omega_4^f(p) = \quad 3.6\times10^{3}\ \mathrm{s}^{-1}$$

$$W_4^b = \sum_{p=1}^{64} \omega_4^b(p) = \quad 1.3\times10^{3}\ \mathrm{s}^{-1}$$

9.4. Matrix elements calculations

The matrix elements $\langle\psi J\|L+g_S S\|\psi^* J^*\rangle$ of the angular L and spin S operators for Nd^{III} transitions were estimated using Rajnak's wavefunctions in the intermediate coupling scheme.[58] Taking into account the selection rules, as mentioned before, the non-null contributions are regarding transitions that obey $\Delta J = J - J^* = 0, \pm 1$ (with $J = J^* = 0$ excluded, but there are no Nd^{III} transitions within this condition). Thus, the following transitions remains:

$$\left[{}^2H_{9/2}, {}^4F_{7/2}, {}^4F_{9/2}, {}^2H_{11/2}, {}^4G_{7/2}, {}^4G_{9/2}, {}^4G_{11/2}\right] \leftrightarrow {}^4I_{9/2}$$

$$\left[{}^2H_{9/2}, {}^4F_{9/2}, {}^2H_{11/2}, {}^4G_{9/2}, {}^2K_{13/2}, {}^4G_{11/2}\right] \leftrightarrow {}^4I_{11/2}$$

$$\left[{}^2H_{11/2}, {}^2K_{13/2}, {}^4G_{11/2}, {}^2K_{15/2}\right] \leftrightarrow {}^4I_{13/2}$$

$$\left[{}^2K_{13/2}, {}^2K_{15/2}\right] \leftrightarrow {}^4I_{15/2}$$

So, the matrix element $\langle\psi J\|L+g_S S\|\psi^* J^*\rangle$ can be separated into two contributions:

$$\begin{aligned}\langle\psi J\|L+g_S S\|\psi^* J^*\rangle &= \langle\psi J\|L\|\psi^* J^*\rangle + g_S\langle\psi J\|S\|\psi^* J^*\rangle \\ &= \sum_{i,L,S} a_i\,\langle LSJ\|L\|\mathrm{LS}J^*\rangle + g_S\sum_{j,L,S} b_j\,\langle LSJ\|S\|\mathrm{LS}J^*\rangle\end{aligned} \tag{S16}$$

where $g_S \cong 2.0023$ is the electron g-factor. Using Rajnak's intermediate coupling wavefunctions, getting the product into the same LS term, and applying the expressions below,

$$\langle LSJ\|L\|LSJ^*\rangle = (-1)^{L+S+J+1}\sqrt{(2J+1)(2J^*+1)L(L+1)(2L+1)}\begin{Bmatrix} J & 1 & J^* \\ L & S & L\end{Bmatrix} \tag{S17}$$

$$\langle LSJ\|S\|LSJ^*\rangle = (-1)^{L+S+J^*+1}\sqrt{(2J+1)(2J^*+1)S(S+1)(2S+1)}\begin{Bmatrix} S & J & L \\ J^* & S & 1\end{Bmatrix} \tag{S18}$$

we can evaluate Eq. S16.

As an example, we describe in detail the calculus of the $\langle{}^2H_{9/2}\|L+g_S S\|{}^4I_{9/2}\rangle$:

1) Describing each term as a linear combination in the intermediate coupling scheme:

$$\begin{aligned}|{}^4I_{9/2}\rangle = &-0.0031|{}^4F\rangle - 0.0077|{}^4G\rangle - 0.9847|{}^4I\rangle + 0.0168|{}^2G\rangle - 0.0148|{}^2G'\rangle - 0.0566|{}^2H\rangle \\ &+ 0.1631|{}^2H'\rangle\end{aligned}$$

$$|{}^2H_{9/2}\rangle = 0.377|{}^4F\rangle + 0.144|{}^4G\rangle - 0.1505|{}^4I\rangle - 0.3382|{}^2G\rangle + 0.2798|{}^2G'\rangle + 0.2868|{}^2H\rangle - 0.7346|{}^2H'\rangle$$

2) Considering $\hat{H}$ a Hermitian operator, we can describe the matrix elements as follows:

$\langle {}^2H_{9/2} \| \hat{H} \| {}^4I_{9/2} \rangle$

$$= -0.0031 \cdot 0.377 \langle {}^4F \| \hat{H} \| {}^4F \rangle - 0.0077 \cdot 0.144 \langle {}^4G \| \hat{H} \| {}^4G \rangle + 0.9847 \cdot 0.1505 \langle {}^4I \| \hat{H} \| {}^4I \rangle - 0.0168 \cdot 0.3382 \langle {}^2G \| \hat{H} \| {}^2G \rangle - 0.0148 \cdot 0.2798 \langle {}^2G' \| \hat{H} \| {}^2G' \rangle - 0.0566 \cdot 0.2868 \langle {}^2H \| \hat{H} \| {}^2H \rangle - 0.1631 \cdot 0.7346 \langle {}^2H' \| \hat{H} \| {}^2H' \rangle$$

3) Applying Eq. S17 (if $\hat{H} = L$) and Eq. S18 (if $\hat{H} = S$) for each $\langle LS \| \hat{H} \| LS \rangle$ term:

$$\langle {}^4F \| \mathrm{L} \| {}^4F \rangle = (-1)^{3+\frac{3}{2}+\frac{9}{2}+1} \sqrt{\left(2\frac{9}{2}+1\right)\left(2\frac{9}{2}+1\right)3(3+1)(2\cdot 3+1)} \begin{Bmatrix} 9/2 & 1 & 9/2 \\ 3 & 3/2 & 3 \end{Bmatrix}$$

$$\langle {}^4F \| \mathrm{S} \| {}^4F \rangle = (-1)^{3+\frac{3}{2}+\frac{9}{2}+1} \sqrt{\left(2\frac{9}{2}+1\right)\left(2\frac{9}{2}+1\right)\frac{3}{2}\left(\frac{3}{2}+1\right)\left(2\cdot\frac{3}{2}+1\right)} \begin{Bmatrix} 3/2 & 9/2 & 3 \\ 9/2 & 3/2 & 1 \end{Bmatrix}$$

where the 6-j symbol can be evaluated using the Wigner coefficient calculator web tool by Anthony Stone:[62]

$$\begin{Bmatrix} 9/2 & 1 & 9/2 \\ 3 & 3/2 & 3 \end{Bmatrix} = \frac{1}{2}\sqrt{\frac{11}{210}}$$

and

$$\begin{Bmatrix} 3/2 & 9/2 & 3 \\ 9/2 & 3/2 & 1 \end{Bmatrix} = \frac{1}{10}\sqrt{\frac{11}{6}}$$

Leading to:

$$\langle {}^4F \| \mathrm{L} \| {}^4F \rangle = 10.49 \text{ and } \langle {}^4F \| \mathrm{S} \| {}^4F \rangle = 5.24$$

where these terms times the product $(-0.0031 \cdot 0.377)$ enter as the first contribution in the equation of point **2**.

4) Calculate the other LS terms until complete Eq. S16.

Once the Yb^{III} has only one 4f transition and it is into the same LS term, the $\langle {}^2F_{5/2} \| L + g_S S \| {}^2F_{7/2} \rangle^2 = 2.5719$ is obtained by the direct application of Eq. S16 with $a_1 = b_1 = 1$ and there is no other contribution ($a_i = b_j = 0$ for i and $j > 1$). Table S12 shows the $\langle \psi_A^* J_A^* \| U^{(\lambda)} \| \psi_A J_A \rangle^2$ and $\langle \psi_A^* J_A^* \| L + g_S S \| \psi_A J_A \rangle^2$ used to calculate the pairwise energy transfer in Tables S8–S11.

Table S12. Matrix elements used to obtain the pairwise energy transfer rates. The values of $\langle \|U^{(\lambda)}\| \rangle^2$ were taken from Ref. [59] while the $\langle \|L + g_S S\| \rangle^2$ were calculated from Rajnak's wavefunctions.[58] For the Yb^{III} $^2F_{5/2} \rightarrow {}^2F_{7/2}$ transition, $\langle {}^2F_{7/2} \| U^{(\lambda)} \| {}^2F_{5/2} \rangle^2$ = 0.1225, 0.4096, and 0.8575 (for λ = 2, 4, and 6, respectively)[45] and $\langle {}^2F_{7/2} \| L + g_S S \| {}^2F_{5/2} \rangle^2$ = 2.5719. All matrix elements are in units of $\hbar$.

Pathway (p)	Nd^{III} transition	$\langle \|\boldsymbol{U}^{(2)}\| \rangle^2$	$\langle \|\boldsymbol{U}^{(4)}\| \rangle^2$	$\langle \|\boldsymbol{U}^{(6)}\| \rangle^2$	$\langle \|\boldsymbol{L} + \boldsymbol{g_S S}\| \rangle^2$
1	$^4F_{3/2}\rightarrow{}^4I_{9/2}$	0	0.2283	0.0554	0
2	$^4F_{5/2}\rightarrow{}^4I_{9/2}$	0.0006	0.2337	0.3983	0
3	$^2H_{9/2}\rightarrow{}^4I_{9/2}$	0.0095	0.0082	0.1195	0.2179
4	$^4F_{7/2}\rightarrow{}^4I_{9/2}$	0.0011	0.0406	0.4272	0.0009
5	$^4S_{3/2}\rightarrow{}^4I_{9/2}$	0	0.0025	0.2347	0
6	$^4F_{9/2}\rightarrow{}^4I_{9/2}$	0.0009	0.0092	0.0406	0.0468
7	$^2H_{11/2}\rightarrow{}^4I_{9/2}$	0	0.0027	0.0104	0.0009
8	$^4G_{5/2}\rightarrow{}^4I_{9/2}$	0.8975	0.4126	0.0346	0
9	$^2G_{7/2}\rightarrow{}^4I_{9/2}$	0.0707	0.172	0.0274	0
10	$^4G_{7/2}\rightarrow{}^4I_{9/2}$	0.0596	0.1709	0.0566	0.0004
11	$^4G_{9/2}\rightarrow{}^4I_{9/2}$	0.0044	0.0584	0.0383	0.0125
12	$^2K_{13/2}\rightarrow{}^4I_{9/2}$	0.0071	0.0002	0.033	0
13	$^2D_{3/2}\rightarrow{}^4I_{9/2}$	0	0.0202	0.0001	0
14	$^4G_{11/2}\rightarrow{}^4I_{9/2}$	0	0.0052	0.0079	0.0001
15	$^2K_{15/2}\rightarrow{}^4I_{9/2}$	0	0.0052	0.0149	0
16	$^2P_{1/2}\rightarrow{}^4I_{9/2}$	0	0.0396	0	0
17	$^4F_{3/2}\rightarrow{}^4I_{11/2}$	0	0.1423	0.4083	0
18	$^4F_{5/2}\rightarrow{}^4I_{11/2}$	0	0.1698	0.0369	0
19	$^2H_{9/2}\rightarrow{}^4I_{11/2}$	0.0028	0.0004	0.0254	0.2159
20	$^4F_{7/2}\rightarrow{}^4I_{11/2}$	0.0009	0.2335	0.3076	0
21	$^4S_{3/2}\rightarrow{}^4I_{11/2}$	0	0	0.2099	0
22	$^4F_{9/2}\rightarrow{}^4I_{11/2}$	0.0001	0.0328	0.3702	0.0497
23	$^2H_{11/2}\rightarrow{}^4I_{11/2}$	0.0043	0.0094	0.0062	0.0702
24	$^4G_{5/2}\rightarrow{}^4I_{11/2}$	0	0.2867	0.0961	0
25	$^2G_{7/2}\rightarrow{}^4I_{11/2}$	0.3996	0.1764	0.0522	0
26	$^4G_{7/2}\rightarrow{}^4I_{11/2}$	0.6684	0.1075	0.0099	0
27	$^4G_{9/2}\rightarrow{}^4I_{11/2}$	0.1403	0.3495	0.0505	0.0106
28	$^2K_{13/2}\rightarrow{}^4I_{11/2}$	0	0	0	0.1072
29	$^2D_{3/2}\rightarrow{}^4I_{11/2}$	0	0.0016	0.0326	0
30	$^4G_{11/2}\rightarrow{}^4I_{11/2}$	0	0	0	0.0003
31	$^2K_{15/2}\rightarrow{}^4I_{11/2}$	0.0020	0.0003	0.0039	0
32	$^2P_{1/2}\rightarrow{}^4I_{11/2}$	0	0	0	0
33	$^4F_{3/2}\rightarrow{}^4I_{13/2}$	0	0	0.2093	0
34	$^4F_{5/2}\rightarrow{}^4I_{13/2}$	0	0.1817	0.4010	0
35	$^2H_{9/2}\rightarrow{}^4I_{13/2}$	0.0389	0.0064	0.1217	0
36	$^4F_{7/2}\rightarrow{}^4I_{13/2}$	0	0.3314	0.0001	0
37	$^4S_{3/2}\rightarrow{}^4I_{13/2}$	0	0	0.3295	0
38	$^4F_{9/2}\rightarrow{}^4I_{13/2}$	0.0029	0.2148	0.5102	0
39	$^2H_{11/2}\rightarrow{}^4I_{13/2}$	0.0043	0.0168	0.0029	0.2493
40	$^4G_{5/2}\rightarrow{}^4I_{13/2}$	0	0.0342	0.0485	0
41	$^2G_{7/2}\rightarrow{}^4I_{13/2}$	0	0.0875	0.0345	0
42	$^4G_{7/2}\rightarrow{}^4I_{13/2}$	0	0.2407	0.0613	0
43	$^4G_{9/2}\rightarrow{}^4I_{13/2}$	0.9552	0.3843	0.0157	0

44	$^2K_{13/2}\rightarrow{}^4I_{13/2}$	0.0032	0.0001	0.0024	0.0861
45	$^2D_{3/2}\rightarrow{}^4I_{13/2}$	0	0.0016	0.0326	0
46	$^4G_{11/2}\rightarrow{}^4I_{13/2}$	0.1283	0.3514	0.1609	0.0072
47	$^2K_{15/2}\rightarrow{}^4I_{13/2}$	0.0003	0.0003	0.0172	0.1135
48	$^2P_{1/2}\rightarrow{}^4I_{13/2}$	0	0	0	0
49	$^4F_{3/2}\rightarrow{}^4I_{15/2}$	0	0	0.0280	0
50	$^4F_{5/2}\rightarrow{}^4I_{15/2}$	0	0	0.2300	0
51	$^2H_{9/2}\rightarrow{}^4I_{15/2}$	0	0.2155	0.0775	0
52	$^4F_{7/2}\rightarrow{}^4I_{15/2}$	0	0.1553	0.6166	0
53	$^4S_{3/2}\rightarrow{}^4I_{15/2}$	0	0	0.3306	0
54	$^4F_{9/2}\rightarrow{}^4I_{15/2}$	0	0.5000	0.4628	0
55	$^2H_{11/2}\rightarrow{}^4I_{15/2}$	0.1293	0.0687	0	0
56	$^4G_{5/2}\rightarrow{}^4I_{15/2}$	0	0	0.0046	0
57	$^2G_{7/2}\rightarrow{}^4I_{15/2}$	0	0.0010	0.1064	0
58	$^4G_{7/2}\rightarrow{}^4I_{15/2}$	0	0.0273	0.0045	0
59	$^4G_{9/2}\rightarrow{}^4I_{15/2}$	0	0.1426	0.2398	0
60	$^2K_{13/2}\rightarrow{}^4I_{15/2}$	0	0.0009	0.0064	0.0008
61	$^2D_{3/2}\rightarrow{}^4I_{15/2}$	0	0	0.0083	0
62	$^4G_{11/2}\rightarrow{}^4I_{15/2}$	1.5301	0.8915	0.159	0
63	$^2K_{15/2}\rightarrow{}^4I_{15/2}$	0.0105	0.0001	0.0231	0.2506
64	$^2P_{1/2}\rightarrow{}^4I_{15/2}$	0	0	0	0

9.5. Simulations of the Nd–Yb distribution and the average energy transfer rates

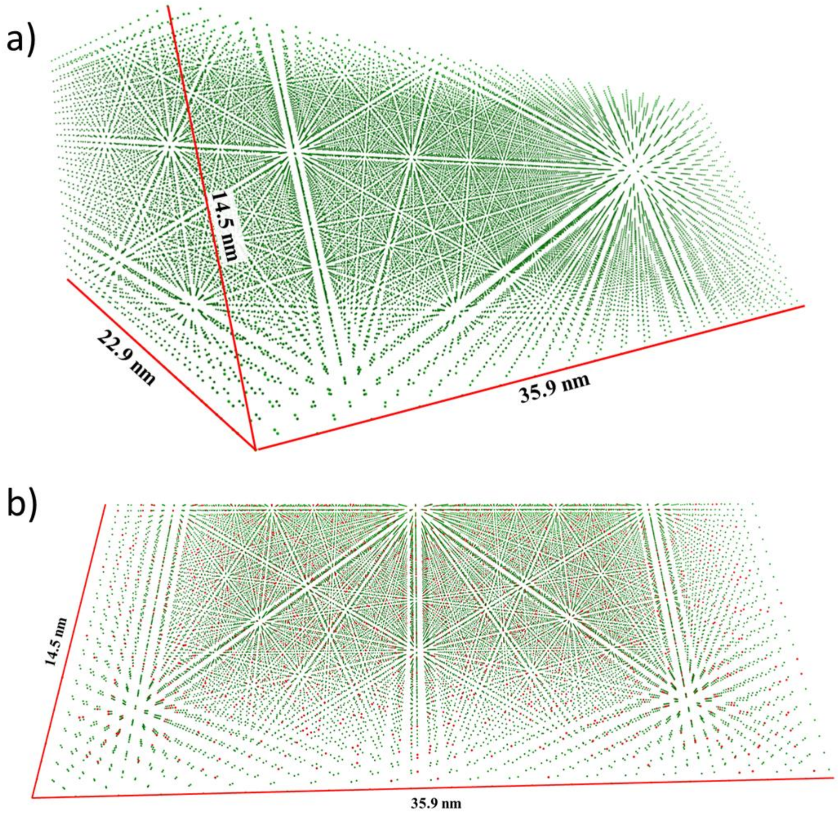

Figure S14. a) Expanded crystallographic structure showing only Nd^{III} sites to be replaced with Yb^{III} ion. b) Example of one doping Monte-Carlo simulation with Yb^{III} $(1 - x = 0.110)$ where red atoms represent the Yb^{III} while Nd^{III} by the green ones.

Table S13. Nd–Yb pair occurrence coefficients $O_i(x)$ and $O_i(1-x)$ (unitless), where index i (1, 2, 3, and 4) represents the order of Nd–Yb distances (5.846, 7.032, 7.258, and 8.659 Å, respectively). These values were obtained from 100 doping simulations with Yb^{III} in expanded 20×20×20 matrices of $Nd(BTC)_3(H_2O)_6$ which contains $s = 32000$ Nd^{III} sites (Eq. 5). $\langle W \rangle^f$ and $\langle W \rangle^b$ (in s^{-1}) are the average energy transfer rates at 300 K. $\langle W \rangle^f$ is almost temperature independent while $\langle W \rangle^b$ has a strong thermal dependence (Figure S15)

Yb^{III} amount		forward energy transfer				
$\mathbf{1-x}$	Sample	$\mathbf{O_1(1-x)}$	$\mathbf{O_2(1-x)}$	$\mathbf{O_3(1-x)}$	$\mathbf{O_4(1-x)}$	$\langle W \rangle^f$
0.047	(3)	1.812	1.810	1.811	1.812	1.924E+04
0.057	(2)	1.794	1.793	1.791	1.793	2.286E+04
0.110	(4)	1.691	1.692	1.692	1.692	3.928E+04
0.144	-	1.626	1.629	1.627	1.629	4.759E+04
0.200	-	1.520	1.521	1.521	1.520	5.771E+04
Nd^{III} amount		backward energy transfer				
$\mathbf{x}$	Sample	$\mathbf{O_1(x)}$	$\mathbf{O_2(x)}$	$\mathbf{O_3(x)}$	$\mathbf{O_4(x)}$	$\langle W \rangle^b$
0.953	(3)	0.089	0.089	0.089	0.089	3.350E+02
0.943	(2)	0.108	0.108	0.108	0.108	4.877E+02
0.890	(4)	0.209	0.209	0.209	0.209	1.714E+03
0.856	-	0.273	0.274	0.274	0.274	2.826E+03
0.800	-	0.380	0.380	0.380	0.380	5.093E+03

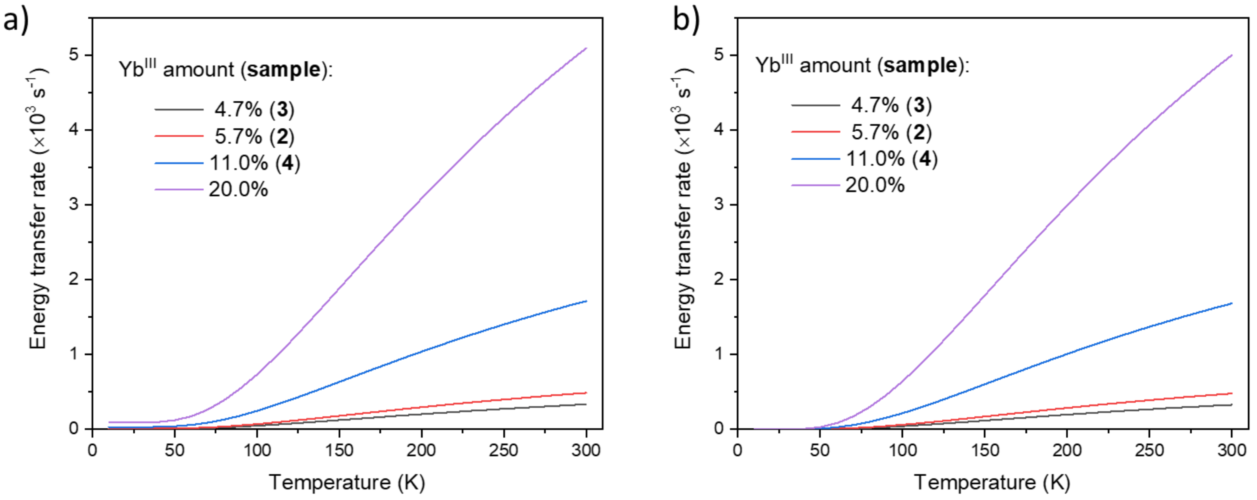

Figure S15. Backward energy transfer rates (Yb–Nd) as a function of the temperature obtained from the doping simulations and considering a) all pathways and b) only the main contribution from Yb^{III} [$^2F_{5/2} \rightarrow {}^2F_{7/2}$] ⇒ Nd^{III} [$^4I_{15/2} \rightarrow {}^2H_{11/2}$] (pathway 55 in Tables S8-S11).

9.6. Rate equations modeling

Considering the levels $|N\rangle$ depicted in Figure 3D, the following eleven-levels coupled rate equations can be described as:

$|0\rangle$
$$\frac{d}{dt}P_0(t) = W_{1\to0}P_1(t) + \frac{1}{\tau_{Nd}}P_4(t) + \frac{1}{\tau_{Yb}}P_{10}(t) + W_{3\to8}P_{10}(t)P_3(t) - \left(\phi + W_{0\to1} + W_{8\to3}P_8(t) + W_{7\to2}P_7(t) + W_{5\to1}P_5(t)\right)P_0(t) \tag{S19}$$

$|1\rangle$
$$\frac{d}{dt}P_1(t) = W_{0\to1}P_0(t) + W_{2\to1}P_2(t) + W_{5\to1}P_5(t)P_0(t) - (W_{1\to0} + W_{1\to2})P_1(t) \tag{S20}$$

$|2\rangle$
$$\frac{d}{dt}P_2(t) = W_{3\to2}P_3(t) + W_{1\to2}P_1(t) + W_{7\to2}P_7(t)P_0(t) - (W_{2\to1} + W_{2\to3})P_2(t) \tag{S21}$$

$|3\rangle$
$$\frac{d}{dt}P_3(t) = W_{8\to3}P_8(t)P_0(t) + W_{2\to3}P_2(t) - \left(W_{3\to2} + W_{3\to8}P_{10}(t)\right)P_3(t) \tag{S22}$$

$|4\rangle$
$$\frac{d}{dt}P_4(t) = W_{5\to4}P_5(t) - \left(W_{4\to5} + \frac{1}{\tau_{Nd}}\right)P_4(t) \tag{S23}$$

$|5\rangle$
$$\frac{d}{dt}P_5(t) = W_{6\to5}P_6(t) + W_{4\to5}P_4(t) - \left(W_{5\to4} + W_{5\to6} + W_{5\to1}P_0(t)\right)P_5(t) \tag{S24}$$

$|6\rangle$
$$\frac{d}{dt}P_6(t) = W_{5\to6}P_5(t) + W_{7\to6}P_7(t) - (W_{6\to5} + W_{6\to7})P_6(t) \tag{S25}$$

$|7\rangle$
$$\frac{d}{dt}P_7(t) = W_{6\to7}P_6(t) + W_{8\to7}P_8(t) - \left(W_{7\to6} + W_{7\to8} + W_{7\to2}P_0(t)\right)P_7(t) \tag{S26}$$

$|8\rangle$
$$\frac{d}{dt}P_8(t) = W_{7\to8}P_7(t) + W_{9\to8}P_9(t) + W_{3\to8}P_3(t)P_{10}(t) - \left(W_{8\to7} + W_{8\to9} + W_{8\to3}P_0(t)\right)P_8(t) \tag{S27}$$

$|9\rangle$
$$\frac{d}{dt}P_9(t) = W_{8\to9}P_8(t) + \phi P_0(t) - W_{9\to8}P_9(t) \tag{S28}$$

$|10\rangle$
$$\frac{d}{dt}P_{10}(t) = \left(W_{8\to3}P_8(t) + W_{7\to2}P_7(t) + W_{5\to1}P_5(t)\right)P_0(t) - \left(\frac{1}{\tau_{Yb}} + W_{3\to8}P_3(t)\right)P_{10}(t) \tag{S29}$$

where $W_{N\to N+1}$ and $W_{N+1\to N}$ represent, respectively, the multiphonon absorption (Eq. S8) and decay (Eq. S5) rates between $|N\rangle$ and $|N+1\rangle$ levels. τ_{Nd} and τ_{Yb} are the decay lifetimes of the Nd^{III} $^4F_{3/2}$ and Yb^{III} $^2F_{5/2}$ emitting levels. ϕ is the pumping rate (Eq. S1) and $W_{N\to M}$ (with $M \neq N \pm 1$) are the forward (if $N > M$) and backward (if $N < M$) energy transfer rates according to the diagram in Figure 3D. For example, in Eq. S29, $W_{8\to3}$ ($|8\rangle \to |3\rangle$ in Figure 3D) is the energy transfer rate from Nd^{III} $^2H_{11/2} \to {}^4I_{15/2}$ to Yb^{III} $^2F_{7/2} \to {}^2F_{5/2}$ while $W_{3\to8}$ is the corresponding backward rate ($Yb^{III} \to Nd^{III}$).

The simulations employed here consider the following condition which guarantee the conservation of the populations for each center (Nd^{III} and Yb^{III}),[63]

$$\frac{1}{t_f}\sum_i \int_0^{t_f} P_i(t)dt = 1 \qquad \text{(S30)}$$

where t_f is the final time of the simulation and $P_i(t)$ is the population of ith state at the time t.

Figure S16 shows the population evolution as a function of the time of Nd^{III} $^4F_{3/2}$ and Yb^{III} $^2F_{5/2}$ emitting levels for sample **4** (11% of Yb^{III}) at 300 K. It can be noted that the Yb^{III} emitting level is more than 100 times higher than the Nd^{III} one and this is attributed to the involvement of the Nd^{III} $^4H_{11/2}$ as the main feeding channel to the Yb^{III} $^2F_{5/2}$ and the fast decay lifetime of the Nd^{III} $^4F_{3/2}$ level.

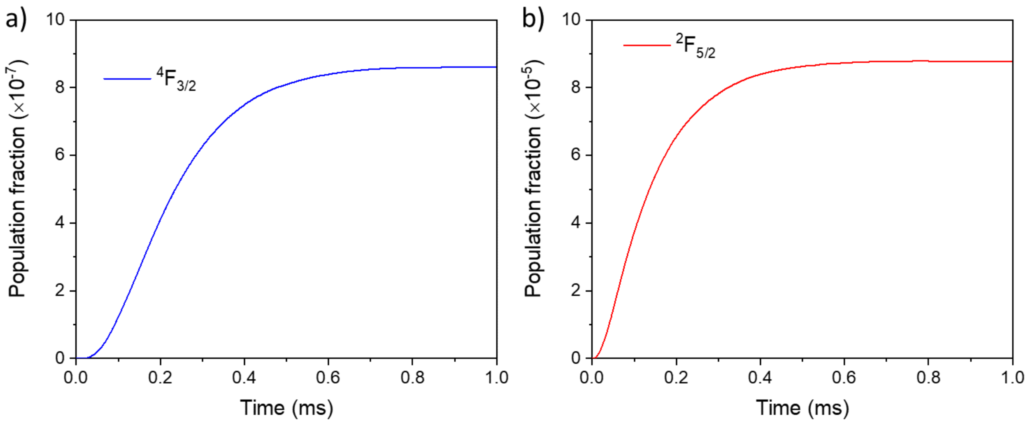

Figure S16. Time evolution of the populations for a) Nd^{III} $^4F_{3/2}$ and b) Yb^{III} $^2F_{5/2}$ levels for sample **4** (11% of Yb^{III}) at 300 K.

9.7. Radiative rates, intensities, and thermometric parameter

Once the theoretical intensity parameters Ω_λ were determined, it is possible to calculate the individual radiative rate $A_{J\to J'}$ (also known as spontaneous emission coefficients):

$$A_{J\to J'} = \frac{4e^2(\omega_{J\to J'})^3}{3\hbar c^3(2J+1)}\left[\frac{n_{ref}(n_{ref}{}^2+2)^2}{9}S_{ed} + n_{ref}{}^3 S_{md}\right] \tag{S31}$$

where,

$$S_{ed} = \sum_{\lambda=2,4,6} \Omega_\lambda \langle l^N\psi J\|U^{(\lambda)}\|l^N\psi' J'\rangle^2 \tag{S32}$$

$$S_{md} = \frac{\hbar}{4m_e^2c^2}\langle l^N\psi J\|L+2S\|l^N\psi' J'\rangle^2 \tag{S33}$$

are the electric and magnetic dipole strength, respectively. The squared matrix elements $\langle l^N\psi J\|U^{(\lambda)}\|l^N\psi' J'\rangle^2$ and $\langle l^N\psi J\|L+2S\|l^N\psi' J'\rangle$ (ΔJ = 0, ±1 with $J = J' = 0$ excluded) for Nd^{III} and Yb^{III} are presented in Table S12. The $\omega_{J\to J'}$ is the angular frequency of the transition $|l^N\psi J\rangle \to |l^N\psi' J'\rangle$ ($^5D_0 \to {}^7F_{J'}$ or $^5D_4 \to {}^7F_{J'}$), m_e is the electron mass, n_{ref} is the refractive index of the medium (considered here equal to 1.5). Thus, for the Nd^{III} $^4F_{3/2}\to{}^4I_{11/2}$ and Yb^{III} $^2F_{5/2}\to{}^2F_{7/2}$ emissions we obtain $A_{rad}(Nd) = 786$ s^{-1} and $A_{rad}(Yb) = 1028$ s^{-1}.

The intensity of Nd^{III} $^4F_{3/2}\to{}^4I_{11/2}$ and Yb^{III} $^2F_{5/2}\to{}^2F_{7/2}$ transitions can be calculated by:

$$I_{Ln} = A_{rad}P_E \tag{S34}$$

using the values of the emitting levels populations P_E (Nd^{III} $^4F_{3/2}$ and Yb^{III} $^2F_{5/2}$ in the steady-state regime) we can estimate the intensities I_{Nd} and I_{Yb} and, afterwards, the theoretical thermometric parameter $\Delta = \frac{I_{Yb}}{I_{Nd}}$. Thus,

$$\Delta = \frac{I_{Yb}}{I_{Nd}} = \frac{A_{rad}(Yb)}{A_{rad}(Nd)}\cdot\frac{P_{10}}{P_4} \tag{S35}$$

, as presented in Eq. 7 (see the main text).

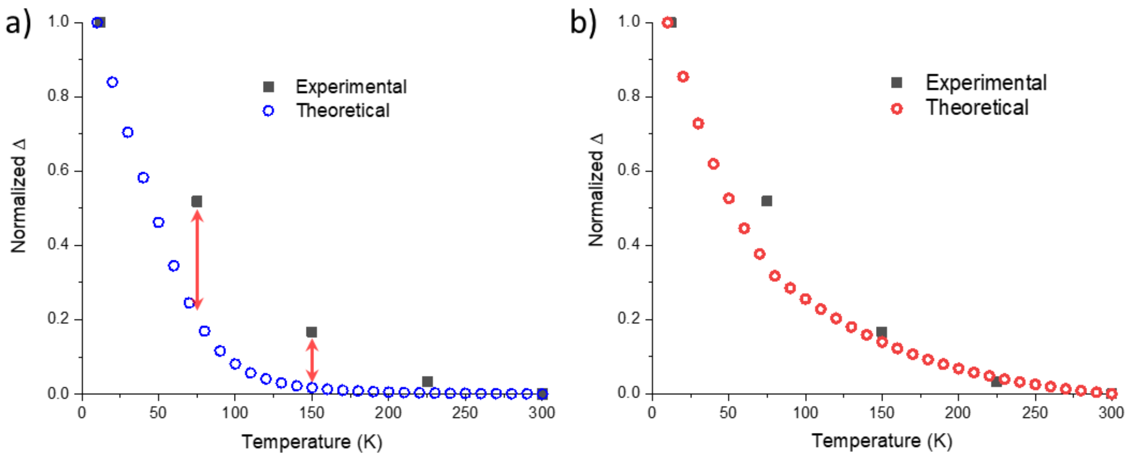

Figure S17. Normalized Δ when the theoretical curve (blue circles) is simulated with a) τ_{Nd} = 50 ns and $\tau_{Yb} \approx 7.1$ µs for any temperature. b) Comparison between theoretical and experimental data if both τ are longer for lower temperatures. The experimental points are represented by black squares. The red arrows are guides indicating the discrepancy between the theoretical results regarding the experimental ones.

10. Supplementary Text 1: An Estimate of Specific Surface Areas

We computed the Specific Surface Areas and other physico-chemical features of our samples from the structural parameters in our hands; they are synoptically collected in the Table S14.

Table S14. Physico-chemical properties of Nd(BTC)(H_2O)$_6$ and Yb(BTC).

**Sample**	**Nd(BTC)(H_2O)$_6$**	**Yb(BTC)**
Density, g·cm^{-3}	2.29	2.96
Coherent Domain Size, nm (*from XRD measurements*)	**83.2**	**26.5**
Coherent Domain Surface, nm^2, spherical approximation	21747	2206
Coherent Domain Surface, m^2, spherical approximation	2.17E−14	2.21E−15
Coherent Domain Volume, nm^3, spherical approximation	301556	9744
Coherent Domain Weight, g, spherical approximation	6.90E−16	2.8E−17
Specific Surface Area = Surface / Weight, m^2·g^{-1}	31.5	76.5
Voids percentage, Probe Radius 1.2 Å	0.0%	0.0%
Voids percentage, Probe Radius 0.5 Å	0.4%	0.5%

Note that the values in **bold**, determined by XRD methods, assume a monodisperse material with spherical particles. Also, these values are *underestimated* because the presence of microstrain (providing additional XRD peak broadening) and the possibility of grain agglomeration will definitely *decrease* the actual SSA.

Note also that porous materials possess much larger SSA values (in m^2·g^{-1}, 300-5000 for activated carbons, 1000 for zeolites – faujasite - and up to 10000 for MOFs). As anticipated, our materials are non-porous.

This is also manifested by the Solvent Accessible Volume, calculated by Mercury on the CCDC-deposited cif files, which becomes non-zero only with a probe radius of 0.5 Å, that is a moiety smaller than a single Hydrogen atom (Bohr radius: 0.529 Å; Van der Waals radius: 1.10 Å). Typical Probe radii are 1.2 Å and above, and, for such a case (and also down to 0.6 Å), the Solvent Accessible Volume, in both crystal phases, is 0.0.

Summarizing, these results clearly witness the absence of any structural (not intergrain) porosity of the investigated materials.

11. Supplementary Text 2: Thermal Stability in Air.

Thermal stability is important in N_2/O_2 if easily oxidizable components are present. Ln^{III} ions are already in a high oxidation state. BTC can indeed decarboxylate at high T in air. However, its thermal stability appears to be exceptionally high. Indeed, Zhang *et al.* [64] reported that solid trimesic acid is stable up to 380 °C. To support this evidence with experimental data, we performed a complete thermal characterization by Variable Temperature X-ray Diffraction (VT-XRD).

Experimental: Powders of $Nd(BTC)(H_2O)_6$ (a) and of Yb(BTC) (b) were deposited in the hollow of an aluminum sample holder positioned in a custom-made heating stage provided by Officina Elettrotecnica di Tenno. Data were collected in the significant 2θ range (8-36° for (a) and 12-37° for (b), $\Delta 2\theta = 0.02°$, t = 1 s step^{-1}) under isothermal conditions, starting from RT (25 °C), in 20 °C (a) or 50 °C (b) steps.

2D plots of the VT-XRD data are shown in Figure S18 (a and b panels, from RT – bottom – up to 145 °C for (a) and to nominally 475 °C for (b)). For (a), decomposition near 100° (and water loss) generates a fully amorphous material.

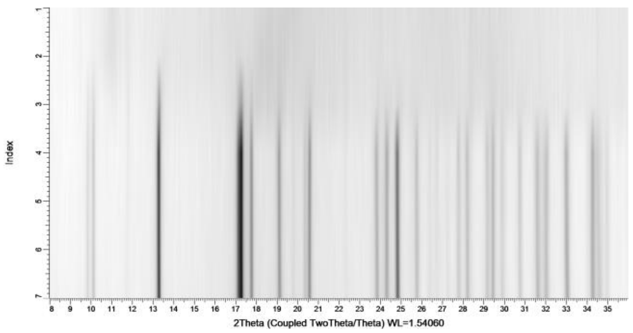

(a) $Nd(BTC)(H_2O)_6$ (RT to 145°C)

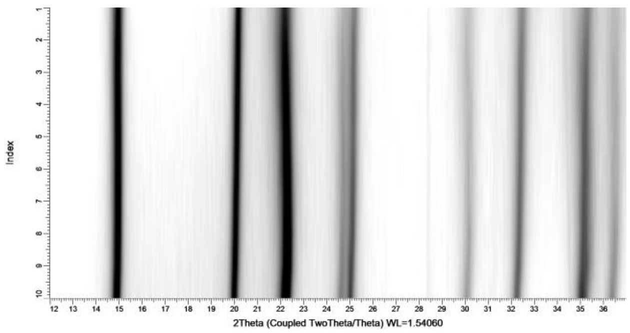

(b) Yb(BTC) (RT to 475°C)

Figure S18. VT-XRD plots for compounds $Nd(BTC)(H_2O)_6$ (a) and Yb(BTC) (b).

Data were numerically analyzed by the structureless Le Bail method implemented in TOPAS-R, which enabled the derivation of the T-dependent cell parameters and the linear thermal expansion coefficients (κ_x, values reported in Table S15). The thermal strain tensors for (a) and (b) (see Figure S19) were then computed using Ohashi's method and visualized by Kaminski's Wintensor program. Each parameter κ_x was assumed to vary with temperature according to:

$$\kappa_x(T, °C) = \kappa_a(25\ °C)(1 + (T - 25))$$

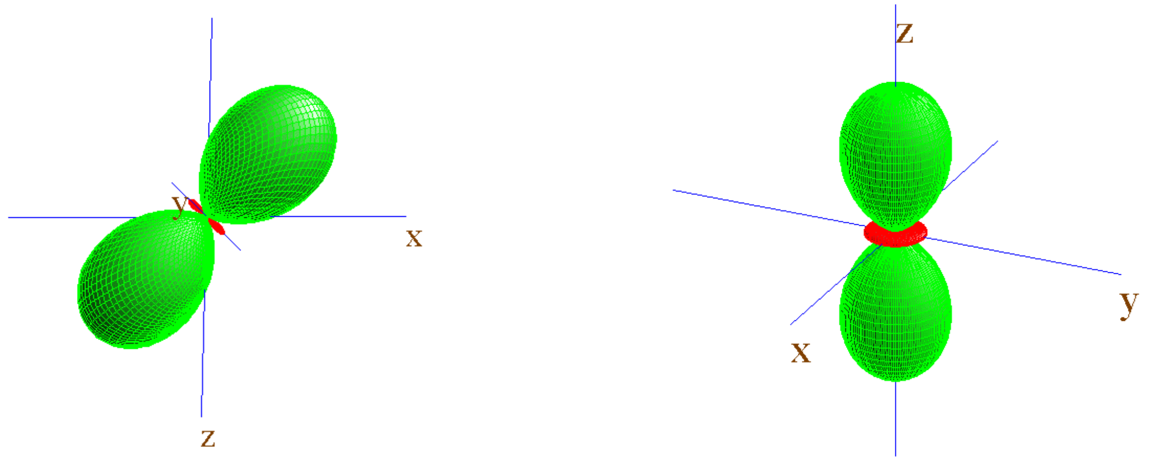

(a) (b)

Figure S19. The Strain tensors for compounds (a) $Nd(BTC)(H_2O)_6$ and (b) Yb(BTC) (not to scale). Crystal axis orientation: in (a), **a** coincides with **x**; **b** coincides with **y**; **z** is nearly oriented as the bisector of the [–x,0,z] quadrant. In (a), **a** coincides with **x**; **c** coincides with **z**; **b** is in the xy plane, 120° away from x.

Table S15. Linear thermal expansion coefficients for $Nd(BTC)(H_2O)_6$ and Yb(BTC).

**Sample**	**$Nd(BTC)(H_2O)_6$**	**Yb(BTC)**
κ_a, MK^{-1}	27	−10
κ_b, MK^{-1}	2	$= \kappa_a$
κ_c, MK^{-1}	14	21
κ_β, MK^{-1}	12	0
κ_V, MK^{-1}	28	41

12. Supplementary Text 3: Textural Properties.

According to the IUPAC classification of adsorption isotherms, the isotherms of both samples **1** and **6** are characteristic of nonporous or macroporous materials. The Brunauer–Emmett–Teller (BET) specific surface area (SSA) is 37 $m^2 \cdot g^{-1}$ for **1** and 20 $m^2 \cdot g^{-1}$ for **6**.

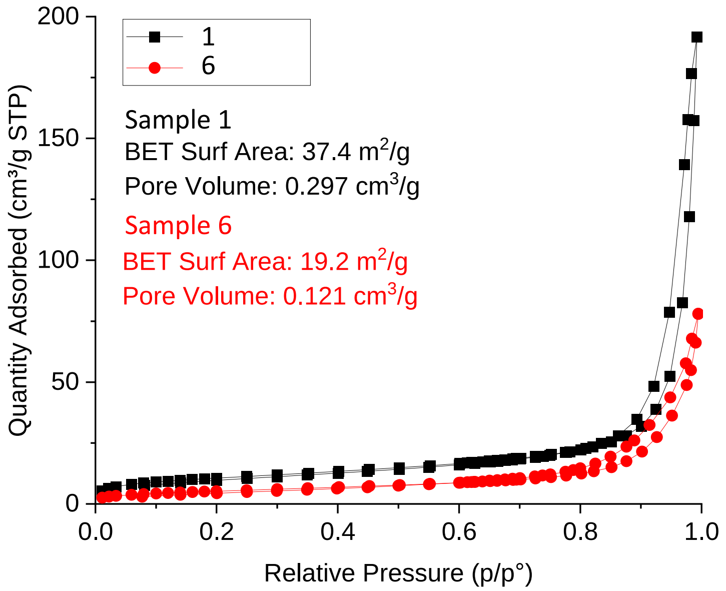

Figure S20. N_2 volumetric adsorption-desorption isotherms of compounds **1** and **6** measured at 77 K.

13. References

(1) TOPAS-R V. 3.0. TOPAS-R, V. 3.0. *Bruker AXS, Karlsruhe, Ger.* **2005**.

(2) Coelho, A. A. Indexing of Powder Diffraction Patterns by Iterative Use of Singular Value Decomposition. *J. Appl. Crystallogr.* **2003**, *36* (1), 86–95. https://doi.org/10.1107/S0021889802019878.

(3) Davies, K.; Bourne, S. A.; Oliver, C. L. Solvent- and Vapor-Mediated Solid-State Transformations in 1,3,5-Benzenetricarboxylate Metal − Organic Frameworks. *Cryst. Growth Des.* **2012**, *12*, 1999–2003.

(4) De Lill, D. T.; Cahill, C. L. An Unusually High Thermal Stability within a Novel Lanthanide 1,3,5-Cyclohexanetricarboxylate Framework: Synthesis, Structure, and Thermal Data. *Chem. Commun.* **2006**, No. 47, 4946–4948. https://doi.org/10.1039/b610012k.

(5) Cheary, R. W.; Coelho, A. Fundamental Parameters Approach to X-Ray Line-Profile Fitting. *J. Appl. Crystallogr.* **1992**, *25* (pt 2), 109–121. https://doi.org/10.1107/S0021889891010804.

(6) Orlova, A. V.; Kozhevnikova, V. Y.; Goloveshkin, A. S.; Lepnev, L. S.; Utochnikova, V. V. NIR Luminescence Thermometers Based on Yb–Nd Coordination Compounds for the 83–393 K Temperature Range. *Dalt. Trans.* **2022**, *51* (14), 5419–5425. https://doi.org/10.1039/D2DT00147K.

(7) Xiang, G.; Yang, M.; Liu, Z.; Wang, Y.; Jiang, S.; Zhou, X.; Li, L.; Ma, L.; Wang, X.; Zhang, J. Near-Infrared-to-Near-Infrared Optical Thermometer BaY_2O_4: Yb^{3+}/Nd^{3+} Assembled with Photothermal Conversion Performance. *Inorg. Chem.* **2022**, *61* (13), 5425–5432. https://doi.org/10.1021/acs.inorgchem.2c00432.

(8) Suo, H.; Zhao, X.; Zhang, Z.; Guo, C. Ultra-Sensitive Optical Nano-Thermometer $LaPO_4$: Yb^{3+}/Nd^{3+} Based on Thermo-Enhanced NIR-to-NIR Emissions. *Chem. Eng. J.* **2020**, *389*, 124506. https://doi.org/10.1016/j.cej.2020.124506.

(9) Gao, G.; Busko, D.; Kauffmann-Weiss, S.; Turshatov, A.; Howard, I. A.; Richards, B. S. Wide-Range Non-Contact Fluorescence Intensity Ratio Thermometer Based on Yb^{3+}/Nd^{3+} Co-Doped La_2O_3 Microcrystals Operating from 290 to 1230 K. *J. Mater. Chem. C* **2018**, *6* (15), 4163–4170. https://doi.org/10.1039/C8TC00782A.

(10) Xu, W.; Hu, Y.; Zheng, L.; Zhang, Z.; Cao, W.; Liu, H.; Wu, X. Enhanced NIR-NIR Luminescence from $CaWO_4$: Nd^{3+}/Yb^{3+} Phosphors by Li^+ Codoping for Thermometry and Optical Heating. *J. Lumin.* **2019**, *208*, 415–423. https://doi.org/10.1016/j.jlumin.2019.01.005.

(11) Chen, H.; Bai, G.; Yang, Q.; Hua, Y.; Xu, S.; Chen, L. Non-Contact Fluorescence Intensity Ratio Optical Thermometer Based on Yb^{3+}/Nd^{3+} Codoped $Bi_4Ti_3O_{12}$ Microcrystals. *J. Lumin.* **2020**, *221*, 117095. https://doi.org/10.1016/j.jlumin.2020.117095.

(12) Xu, W.; Qi, H.; Zheng, L.; Zhang, Z.; Cao, W. Multifunctional Nanoparticles Based on the Nd^{3+}/Yb^{3+} Codoped $NaYF_4$. *Opt. Lett.* **2015**, *40* (23), 5678. https://doi.org/10.1364/OL.40.005678.

(13) Gomez, G. E.; Marin, R.; Carneiro Neto, A. N.; Botas, A. M. P.; Ovens, J.; Kitos, A. A.; Bernini, M. C.; Carlos, L. D.; Soler-Illia, G. J. A. A.; Murugesu, M. Tunable Energy-Transfer Process in Heterometallic MOF Materials Based on 2,6-Naphthalenedicarboxylate: Solid-State Lighting and Near-Infrared Luminescence Thermometry. *Chem. Mater.* **2020**, *32* (17), 7458–7468. https://doi.org/10.1021/acs.chemmater.0c02480.

(14) Walsh, B. M.; McMahon, J. M.; Edwards, W. C.; Barnes, N. P.; Equall, R. W.; Hutcheson, R. L. Spectroscopic Characterization of Nd:Y_2O_3: Application toward a Differential Absorption Lidar System for Remote Sensing of Ozone. *J. Opt. Soc. Am. B* **2002**, *19* (12), 2893. https://doi.org/10.1364/JOSAB.19.002893.

(15) Körner, J.; Krüger, M.; Reiter, J.; Münzer, A.; Hein, J.; Kaluza, M. C. Temperature Dependent Spectroscopic Study of Yb^{3+}-Doped $KG(WO_4)_2$, $KY(WO_4)_2$, $YAlO_3$ and $YLiF_4$ for Laser Applications. *Opt. Mater. Express* **2020**, *10* (10), 2425. https://doi.org/10.1364/OME.398740.

(16) Judd, B. R. Optical Absorption Intensities of Rare-Earth Ions. *Phys. Rev.* **1962**, *127* (3), 750–761. https://doi.org/10.1103/PhysRev.127.750.

(17) Ofelt, G. S. Intensities of Crystal Spectra of Rare-Earth Ions. *J. Chem. Phys.* **1962**, *37* (3), 511–520. https://doi.org/10.1063/1.1701366.

(18) Jørgensen, C. K.; Judd, B. R. Hypersensitive Pseudoquadrupole Transitions in Lanthanides. *Mol. Phys.* **1964**, *8* (3), 281–290. https://doi.org/10.1080/00268976400100321.

(19) Wybourne, B. G. *Spectroscopic Properties of Rare Earths*; John Wiley & Sons: New York, 1965.

(20) Moura Jr., R. T.; Carneiro Neto, A. N.; Longo, R. L.; Malta, O. L. On the Calculation and Interpretation of Covalency in the Intensity Parameters of 4f-4f Transitions in Eu^{3+} Complexes Based on the Chemical Bond Overlap Polarizability. *J. Lumin.* **2016**, *170*, 420–430. https://doi.org/10.1016/j.jlumin.2015.08.016.

(21) Carneiro Neto, A. N.; Teotonio, E. E. S.; de Sá, G. F.; Brito, H. F.; Legendziewicz, J.; Carlos, L. D.; Felinto, M. C. F. C.; Gawryszewska, P.; Moura Jr., R. T.; Longo, R. L.; Faustino, W. M.; Malta, O. L. Modeling Intramolecular Energy Transfer in Lanthanide Chelates: A Critical Review and Recent Advances. In *Handbook on the Physics and Chemistry of Rare Earths, volume 56*; Bünzli, J.-C. G., Pecharsky, V. K., Eds.; Elsevier, 2019; pp 55–162. https://doi.org/10.1016/bs.hpcre.2019.08.001.

(22) Carneiro Neto, A. N.; Moura, R. T.; Shyichuk, A.; Paterlini, V.; Piccinelli, F.; Bettinelli, M.; Malta, O. L. Theoretical and Experimental Investigation of the $Tb^{3+} \rightarrow Eu^{3+}$ Energy Transfer Mechanisms in Cubic $A_3Tb_{0.90}Eu_{0.10}(PO_4)_3$ (A = Sr, Ba) Materials. *J. Phys. Chem. C* **2020**, *124* (18), 10105–10116.

https://doi.org/10.1021/acs.jpcc.0c00759.

(23) Malta, O. L. Mechanisms of Non-Radiative Energy Transfer Involving Lanthanide Ions Revisited. *J. Non. Cryst. Solids* **2008**, *354* (42–44), 4770–4776. https://doi.org/10.1016/j.jnoncrysol.2008.04.023.

(24) Carneiro Neto, A. N.; Moura Jr., R. T. Overlap Integrals and Excitation Energies Calculations in Trivalent Lanthanides 4f Orbitals in Pairs Ln-L (L = Ln, N, O, F, P, S, Cl, Se, Br, and I). *Chem. Phys. Lett.* **2020**, *757*, 137884. https://doi.org/10.1016/j.cplett.2020.137884.

(25) Malta, O. L. Theoretical Crystal-Field Parameters for the YOCl:Eu^{3+} System. A Simple Overlap Model. *Chem. Phys. Lett.* **1982**, *88* (3), 353–356. https://doi.org/10.1016/0009-2614(82)87103-0.

(26) Malta, O. L. A Simple Overlap Model in Lanthanide Crystal-Field Theory. *Chem. Phys. Lett.* **1982**, *87* (1), 27–29. https://doi.org/10.1016/0009-2614(82)83546-X.

(27) Carneiro Neto, A. N.; Moura, R. T.; Aguiar, E. C.; Santos, C. V; de Medeiros, M. A. F. L. B. Theoretical Study of Geometric and Spectroscopic Properties of Eu(III) Complexes with Ruhemann's Purple Ligands. *J. Lumin.* **2018**, *201* (83), 451–459. https://doi.org/10.1016/j.jlumin.2018.05.014.

(28) Carneiro, A. N.; Huskowska, E.; Gawryszewska, P.; Legendziewicz, J.; Malta, O. L. Modeling 4*f*–4*f* Intensity Parameters as a Function of Structural Distortions in Ln (2,2′-Bipyridine-1,1′-Dioxide)$_4$(ClO_4)$_3$ Complexes (Ln=Pr^{3+}, Nd^{3+}). *J. Lumin.* **2016**, *169* (169), 454–457. https://doi.org/10.1016/j.jlumin.2015.02.028.

(29) Moura Jr., R. T.; Carneiro Neto, A. N.; Aguiar, E. C.; Santos-Jr., C. V.; de Lima, E. M.; Faustino, W. M.; Teotonio, E. E. S.; Brito, H. F.; Felinto, M. C. F. C.; Ferreira, R. A. S.; Carlos, L. D.; Longo, R. L.; Malta, O. L. (INVITED) JOYSpectra: A Web Platform for Luminescence of Lanthanides. *Opt. Mater. X* **2021**, *11*, 100080. https://doi.org/10.1016/j.omx.2021.100080.

(30) Reisfeld, R.; Jørgensen, C. K. *Lasers and Excited States of Rare Earths*, 1st ed.; Springer-Verlag Berlin Heidelberg, 1977; Vol. I. https://doi.org/10.1007/978-3-642-66696-4.

(31) Moos, H. W. Spectroscopic Relaxation Processes of Rare Earth Ions in Crystals. *J. Lumin.* **1970**, *1–2* (C), 106–121. https://doi.org/10.1016/0022-2313(70)90027-X.

(32) Malkin, B. Z. Ion-Phonon Interactions. In *Spectroscopic Properties of Rare Earths in Optical Materials*; Hull, R., Parisi, J., Osgood, R. M., Warlimont, H., Liu, G., Jacquier, B., Eds.; Springer Berlin Heidelberg: Berlin, Heidelberg, 2005; pp 130–190. https://doi.org/10.1007/3-540-28209-2_3.

(33) Riseberg, L. A.; Weber, M. J. Relaxation Phenomena in Rare-Earth Luminescence. In *Progress in Optics*; 1977; Vol. 14, pp 89–159. https://doi.org/10.1016/S0079-6638(08)70251-8.

(34) Miyakawa, T.; Dexter, D. L. Phonon Sidebands, Multiphonon Relaxation of Excited States, and Phonon-Assisted Energy Transfer between Ions in Solids. *Phys. Rev. B* **1970**, *1* (7), 2961–2969. https://doi.org/10.1103/PhysRevB.1.2961.

(35) Yamada, N.; Shionoya, S.; Kushida, T. Phonon-Assisted Energy Transfer between Trivalent Rare Earth Ions. *J. Phys. Soc. Japan* **1972**, *32* (6), 1577–1586. https://doi.org/10.1143/JPSJ.32.1577.

(36) Fonger, W. H.; Struck, C. W. Unified Model of Energy Transfer for Arbitrary Franck-Condon Offset and Temperature. *J. Lumin.* **1978**, *17* (3), 241–261. https://doi.org/10.1016/0022-2313(78)90059-5.

(37) Auzel, F.; De Sá, G. F.; de Azevedo, W. M. An Example of Concentration Sensitive Electron-Phonon Coupling in $\{(C_4H_9)_4N\}_3$ $Eu_xY_{1-x}(NCS)_6$ and a New Hypothesis for Self-Quenching. *J. Lumin.* **1980**, *21* (2), 187–192. https://doi.org/10.1016/0022-2313(80)90020-4.

(38) Riseberg, L. A.; Gandrud, W. B.; Moos, H. W. Multiphonon Relaxation of Near-Infrared Excited States of $LaCl_3$:Dy^{3+}. *Phys. Rev.* **1967**, *159* (2), 262–266. https://doi.org/10.1103/PhysRev.159.262.

(39) Luo, Z.; Huang, Y. *Physics of Solid-State Laser Materials*; Springer Series in Materials Science; Springer Singapore: Singapore, 2020; Vol. 289. https://doi.org/10.1007/978-981-32-9668-8.

(40) Vinogradova, E.; Dolgov, L.; Konyushkin, V. A.; Orlovskaya, E. O.; Vagapova, E. A.; Treshchalov, A.; Peet, V.; Hizhnyakov, V.; Orlovskii, Y. V. Fluorescence of Nd^{3+} Optical Centers Close to Cubic Symmetry in a Calcium Fluoride Crystal Co-Doped with Na^+. *J. Lumin.* **2021**, *234* (February), 117988. https://doi.org/10.1016/j.jlumin.2021.117988.

(41) Tofield, B. C.; Weber, H. P. Efficient Phonon-Assisted Long-Lifetime Nd^{3+} Fluorescence in $Cs_2NaNdCl_6$. *Phys. Rev. B* **1974**, *10* (11), 4560–4567. https://doi.org/10.1103/PhysRevB.10.4560.

(42) Zou, K.; Guo, H.; Lu, M.; Li, W.; Hou, C.; Wei, W.; He, J.; Peng, B.; Xiangli, B. Broad-Spectrum and Long-Lifetime Emissions of Nd^{3+} Ions in Lead Fluorosilicate Glass. *Opt. Express* **2009**, *17* (12), 10001. https://doi.org/10.1364/oe.17.010001.

(43) Filho, J. C.; Messias, D. N.; Pilla, V.; Silva, A. C.; Dantas, N. O.; Andrade, A. A. Temperature-Dependence on the Lifetime of Nd^{3+}-Doped Phosphate Glass. *J. Lumin.* **2020**, *219* (July 2019), 116901. https://doi.org/10.1016/j.jlumin.2019.116901.

(44) Padlyak, B.; Ryba-Romanowski, W.; Lisiecki, R. Optical Spectroscopy and Local Structure of the Nd^{3+} Luminescence Centres in Glasses of the CaO-Ga_2O_3-GeO_2 System. *Opt. Appl.* **2008**, *38* (1), 189–202.

(45) Kushida, T. Energy Transfer and Cooperative Optical Transitions in Rare-Earth Doped Inorganic Materials. I. Transition Probability Calculation. *J. Phys. Soc. Japan* **1973**, *34* (5), 1318–1326.

https://doi.org/10.1143/JPSJ.34.1318.

(46) te Velde, G.; Bickelhaupt, F. M.; Baerends, E. J.; Fonseca Guerra, C.; van Gisbergen, S. J. A.; Snijders, J. G.; Ziegler, T. Chemistry with ADF. *J. Comput. Chem.* **2001**, *22* (9), 931–967. https://doi.org/10.1002/jcc.1056.

(47) Lee, C.; Yang, W.; Parr, R. G. Development of the Colle-Salvetti Correlation-Energy Formula into a Functional of the Electron Density. *Phys. Rev. B* **1988**, *37* (2), 785–789. https://doi.org/10.1103/PhysRevB.37.785.

(48) Perdew, J. P. Density-Functional Approximation for the Correlation Energy of the Inhomogeneous Electron Gas. *Phys. Rev. B* **1986**, *33* (12), 8822–8824. https://doi.org/10.1103/PhysRevB.33.8822.

(49) Van Lenthe, E.; Baerends, E. J. Optimized Slater-Type Basis Sets for the Elements 1-118. *J. Comput. Chem.* **2003**, *24* (9), 1142–1156. https://doi.org/10.1002/jcc.10255.

(50) Van Lenthe, E.; Snijders, J. G.; Baerends, E. J. The Zero-Order Regular Approximation for Relativistic Effects: The Effect of Spin-Orbit Coupling in Closed Shell Molecules. *J. Chem. Phys.* **1996**, *105* (15), 6505–6516. https://doi.org/10.1063/1.472460.

(51) Van Lenthe, E.; Baerends, E. J.; Snijders, J. G. Relativistic Total Energy Using Regular Approximations. *J. Chem. Phys.* **1994**, *101* (11), 9783–9792. https://doi.org/10.1063/1.467943.

(52) Van Lenthe, E. Geometry Optimizations in the Zero Order Regular Approximation for Relativistic Effects. *J. Chem. Phys.* **1999**, *110* (18), 8943–8953. https://doi.org/10.1063/1.478813.

(53) Dexter, D. L. A Theory of Sensitized Luminescence in Solids. *J. Chem. Phys.* **1953**, *21* (5), 836–850. https://doi.org/10.1063/1.1699044.

(54) Tanner, P. A.; Chua, M.; Reid, M. F. Energy Transfer by Magnetic Dipole—Magnetic Dipole Interaction. *Chem. Phys. Lett.* **1993**, *209* (5–6), 539–546. https://doi.org/10.1016/0009-2614(93)80130-H.

(55) Chua, M.; Tanner, P. A.; Reid, M. F. Energy Transfer by Electric Dipole-Magnetic Dipole Interaction in Cubic Crystals. *Solid State Commun.* **1994**, *90* (9), 581–583. https://doi.org/10.1016/0038-1098(94)90125-2.

(56) Tanner, P. A.; Chua, M.; Reid, M. F. Energy Transfer between Lanthanide Ions in Elpasolite Lattices. *J. Alloys Compd.* **1995**, *225* (1–2), 20–23. https://doi.org/10.1016/0925-8388(94)07112-8.

(57) Chua, M.; Tanner, P. A.; Reid, M. F. Energy Transfer between Lanthanide Ions in Elpasolite Lattices: Electric Quadrupole-Electric Quadrupole Interaction. *J. Lumin.* **1994**, *58* (1–6), 356–360. https://doi.org/10.1016/0022-2313(94)90436-7.

(58) Rajnak, K. Configuration-Interaction Effects on the "Free-Ion" Energy Levels of Nd^{3+} and Er^{3+}. *J. Chem. Phys.*

1965, *43* (3), 847–855. https://doi.org/10.1063/1.1696857.

(59) Carnall, W. T.; Crosswhite, H.; Crosswhite, H. M. *Energy Level Structure and Transition Probabilities in the Spectra of the Trivalent Lanthanides in LaF_3*; Argonne, IL, United States, 1978. https://doi.org/10.2172/6417825.

(60) Edvardsson, S.; Klintenberg, M. Role of the Electrostatic Model in Calculating Rare-Earth Crystal-Field Parameters. *J. Alloys Compd.* **1998**, *275–277*, 230–233. https://doi.org/10.1016/S0925-8388(98)00309-0.

(61) Malta, O. L.; Brito, H. F.; Menezes, J. F. S.; Silva, F. R. G. e; Alves, S.; Farias, F. S.; de Andrade, A. V. M. Spectroscopic Properties of a New Light-Converting Device Eu(Thenoyltrifluoroacetonate)$_3$ 2(Dibenzyl Sulfoxide). A Theoretical Analysis Based on Structural Data Obtained from a Sparkle Model. *J. Lumin.* **1997**, *75* (3), 255–268. https://doi.org/10.1016/S0022-2313(97)00107-5.

(62) Anthony Stone. Wigner coefficient calculator http://www-stone.ch.cam.ac.uk/wigner.shtml (accessed 2023 -09 -06).

(63) Blois, L.; Carneiro Neto, A. N.; Malta, O. L.; Brito, H. F. The Role of the Eu^{3+} $^7F_1$ Level in the Direct Sensitization of the $^5D_0$ Emitting Level through Intramolecular Energy Transfer. *J. Lumin.* **2022**, 118862. https://doi.org/10.1016/j.jlumin.2022.118862.

(64) Zhang, D.; Xu, Q. Solubility of 1,3,5-Benzenetricarboxylic Acid in Different Solvents. *J. Chem. Eng. Data* **2016**, *61* (2), 1003–1006. https://doi.org/10.1021/acs.jced.5b00870.
